# A Model for Direction Sensing in *Dictyostelium discoideum*: Ras Activity and Symmetry Breaking Driven by a G_βγ_-Mediated, G_α2_-Ric8 -- Dependent Signal Transduction Network

**DOI:** 10.1371/journal.pcbi.1004900

**Published:** 2016-05-06

**Authors:** Yougan Cheng, Hans Othmer

**Affiliations:** School of Mathematics, University of Minnesota, Minneapolis, Minnesota, United States of America; Northeastern University, UNITED STATES

## Abstract

Chemotaxis is a dynamic cellular process, comprised of direction sensing, polarization and locomotion, that leads to the directed movement of eukaryotic cells along extracellular gradients. As a primary step in the response of an individual cell to a spatial stimulus, direction sensing has attracted numerous theoretical treatments aimed at explaining experimental observations in a variety of cell types. Here we propose a new model of direction sensing based on experiments using *Dictyostelium discoideum* (Dicty). The model is built around a reaction-diffusion-translocation system that involves three main component processes: a signal detection step based on G-protein-coupled receptors (GPCR) for cyclic AMP (cAMP), a transduction step based on a heterotrimetic G protein G_*α*_2_*βγ*_, and an activation step of a monomeric G-protein Ras. The model can predict the experimentally-observed response of cells treated with latrunculin A, which removes feedback from downstream processes, under a variety of stimulus protocols. We show that ${\text{G}}_{\alpha_{2}\beta \gamma }$ cycling modulated by Ric8, a nonreceptor guanine exchange factor for ${\text{G}}_{\alpha_{2}}$ in Dicty, drives multiple phases of Ras activation and leads to direction sensing and signal amplification in cAMP gradients. The model predicts that both ${\text{G}}_{\alpha_{2}}$ and G_*βγ*_ are essential for direction sensing, in that membrane-localized ${\text{G}}_{\alpha_{2}}^{*}$, the activated GTP-bearing form of ${\text{G}}_{\alpha_{2}}$, leads to asymmetrical recruitment of RasGEF and Ric8, while globally-diffusing G_*βγ*_ mediates their activation. We show that the predicted response at the level of Ras activation encodes sufficient ‘memory’ to eliminate the ‘back-of-the wave’ problem, and the effects of diffusion and cell shape on direction sensing are also investigated. In contrast with existing LEGI models of chemotaxis, the results do not require a disparity between the diffusion coefficients of the Ras activator GEF and the Ras inhibitor GAP. Since the signal pathways we study are highly conserved between Dicty and mammalian leukocytes, the model can serve as a generic one for direction sensing.

## Author Summary

Many eukaryotic cells, including *Dictyostelium discoideum* (Dicty), neutrophils and other cells of the immune system, can detect and reliably orient themselves in chemoattractant gradients. In Dicty, signal detection and transduction involves a G-protein-coupled receptor (GPCR) through which extracellular cAMP signals are transduced into Ras activation via an intermediate heterotrimeric G-protein (${\text{G}}_{\alpha_{2}\beta \gamma }$). Ras activation is the first *polarized* response to cAMP gradients in Dicty. Recent work has revealed mutiple new characteristics of Ras activation in Dicty, thereby providing new insights into direction sensing mechanisms and pointing to the need for new models of chemotaxis. Here we propose a novel reaction-diffusion model of Ras activation based on three major components: one involving the GPCR, one centered on ${\text{G}}_{\alpha_{2}\beta \gamma }$, and one involving the monomeric G protein Ras. In contrast to existing local excitation, global inhibition (LEGI) models of direction sensing, in which a fast-responding but slowly-diffusing activator and a slow-acting rapidly diffusing inhibitor set up an internal gradient of activity, our model is based on equal diffusion coefficients for all cytosolic species, and the unbalanced local sequestration of some species leads to gradient sensing and amplification. We show that Ric8-modulated ${\text{G}}_{\alpha_{2}\beta \gamma }$ cycling between the cytosol and membrane can account for many of the observed responses in Dicty, including imperfect adaptation, multiple phases of Ras activity in a cAMP gradient, rectified directional sensing, and a solution to the back-of-the-wave problem.

## Introduction

Many eukaryotic cells can detect both the magnitude and direction of extracellular signals using receptors embedded in the cell membrane. When the signal is spatially nonuniform they may respond by directed migration either up or down the gradient of the signal, a process called taxis. When the extracellular signal is an adhesion factor attached to the substrate or extracellular matrix, the response is haptotaxis [1], and when it is a diffusible molecule the process is called chemotaxis. Chemotaxis plays important and diverse roles in different organisms, including mediation of cell-cell communication [2], in organizing and re-organizing tissue during development and wound healing [3–5], in trafficking in the immune system [6], and in cancer metastasis [7].

Chemotaxis can be conceptually divided into three interdependent processes: direction sensing, polarization, and locomotion [8, 9]. In the absence of an external stimulus, cells can extend random pseudopodia and ‘diffuse’ locally, which is referred to as random motility [10]. Direction sensing refers to the molecular mechanism that detects the gradient and generates an internal amplified response, providing an internal compass for the cell [11]. Polarization involves the establishment of an asymmetric shape with a well-defined anterior and posterior, a semi-stable state that allows a cell to move in the same direction without an external stimulus. These three processes are linked through interconnected networks that govern (i) receptor-mediated transduction of an extracellular signal into a primary intracellular signal, (ii) translation of the primary signal into pathway-specific signals for one or more signalling pathways, and (iii) the actin cytoskeleton and auxiliary proteins that determine polarity of the cell. A single extracellular signal may activate numerous pathways, but our focus herein is on the first pathway, which involves transduction of an extracellular cAMP signal via a GPCR, and one specific pathway of the second type, the Ras pathway, which is involved in activating the appropriate downstream networks that govern chemotactic locomotion.

Dicty is an amoeboid eukaryotic cell that utilizes chemotaxis during various stages of its life cycle. In the vegetative phase, it locates a food source by migrating toward folic acid secreted by bacteria or yeast. When the food supply is depleted Dicty undergoes a transformation from the vegetative to the aggregation phase, in which cells sense and migrate toward locally-secreted 3’-5’ cyclic adenosine monophosphate (cAMP), which serves as a messenger for control of chemotaxis and other processes [9, 12]. Dicty has served as an excellent model for studying the interconnected signalling pathways governing chemotaxis due to its genetic and biochemical tractability [13–15]. The major components of the network topology for chemotaxis have been identified by analyzing the effects of gene knockouts and the response of cells to various spatio-temporal signalling protocols [8, 16, 17].

The first step of the chemotactic process involves signal transduction by GPCR’s that activate G-proteins, which is described in detail in the following section. Activated G-proteins can in turn activate numerous pathways, and the pathway we analyze here involves Ras, which is a monomeric G protein that functions as a molecular switch that activates downstream effectors such as PI3K in its activated GTP-bound state. Activation of Ras is the earliest measurable polarized signalling event downstream of G protein activation [14, 18]. A major question from both the experimental and the theoretical viewpoints is how the cell transduces a shallow spatial gradient of extracellular cAMP into a steeper internal gradient of activated Ras. Recent experiments show that Ras activity exhibits multiple temporal phases in cAMP gradients [19]. The first phase is transient activation of Ras that is essentially uniform over the entire cell boundary. In the second phase, symmetry is broken and Ras is reactivated exclusively at the up-gradient side of the cell. The third phase is confinement, in which the crescent of activated Ras localizes further to the region exposed to the highest cAMP. Other recent observations that are not incorporated in existing models are as follows. Firstly, the Ras symmetry breaking does not depend on the presence of the actin cytoskeleton—treatment of cells with latrunculin A (LatA), which leads to depolymerization of the network—does not destroy the symmetry-breaking [19]. Secondly, it was found that when two brief stimuli are applied to the same cell, the response to the second stimulus depends on the interval between the stimuli, which indicates that there is a refractory period [20]. Other experiments show that the adaptation of Ras activation is slightly imperfect, and Ras activity is suppressed when the chemoattractant concentration is decreasing in time, a phenomenon called rectification [21]. Finally, it was reported that there is a persistent memory of Ras activation, even when the cells are treated with LatA [22].

These new results are difficult to interpret in the framework of existing models, a number of which have been proposed [11, 20, 22–29]. Most current models are based on an activator and inhibitor mechanism called LEGI—local excitation, global inhibition—to explain both direction sensing and adaptation when the chemoattractant level is held constant [30]. While these models shed some light on direction sensing, their usefulness is limited due to the oversimplification of the signal transduction network—as will be elaborated later. In particular, none of the existing models incorporates sufficient mechanistic detail to satisfactorily explain the spectrum of observations described above, which provides the rationale for a more comprehensive model that enables us to test hypotheses and make predictions concerning the expected behavior of the signal transduction pathways.

The key components in the model we develop herein are the G-protein ${\text{G}}_{\alpha_{2}\beta \gamma }$, RasGEF and RasGAP, which control rapid excitation and slower adaptation of Ras, and Ric8, a guanine nucleotide exchange factor that activates the ${\text{G}}_{\alpha_{2}}$-component of ${\text{G}}_{\alpha_{2}\beta \gamma }$ [31]. The model is developed for LatA-treated cells so as to remove the feedback effect from the actin cytoskeleton on Ras, and we show that it can replicate many of the observed characteristics of Ras activation in Dicty. It is known that activated Ras activates PI3K, which stimulates further downstream steps that affect actin polymerization, but we can restrict attention to the Ras dynamics and its upstream effectors because there is no known direct feedback to Ras from downstream steps between Ras and the actin cytoskeleton. We show that G_*β*γ_ mediates adaptation of Ras activity in a uniform stimulus and transient activation in a gradient. It is also shown that *G*_*α*2_ contributes to the imperfect adaptation in a uniform stimulus, and that it is an essential element for front-to-back symmetry breaking in a gradient, highlighting the important roles of *G*_*α*2_ and ${\text{G}}_{\alpha_{2}\beta \gamma }$ cycling between the bound and dissociated states. We also show that Ric8 contributes to the amplification of Ras activity by regulating *G*_*α*2_ dynamics: the reactivation of G_*α*2_ by Ric8 induces further asymmetry in ${\text{G}}_{\alpha_{2}\beta \gamma }$ dissociation, which in turn amplifies the Ras activity. Finally, we investigated the effects of diffusion and cell shapes on direction sensing, and the potential role of Ric8 in the establishment of persistent Ras activation, which provides a solution to the back-of-the-wave problem.

### Signal transduction pathways

**Fig 1:**
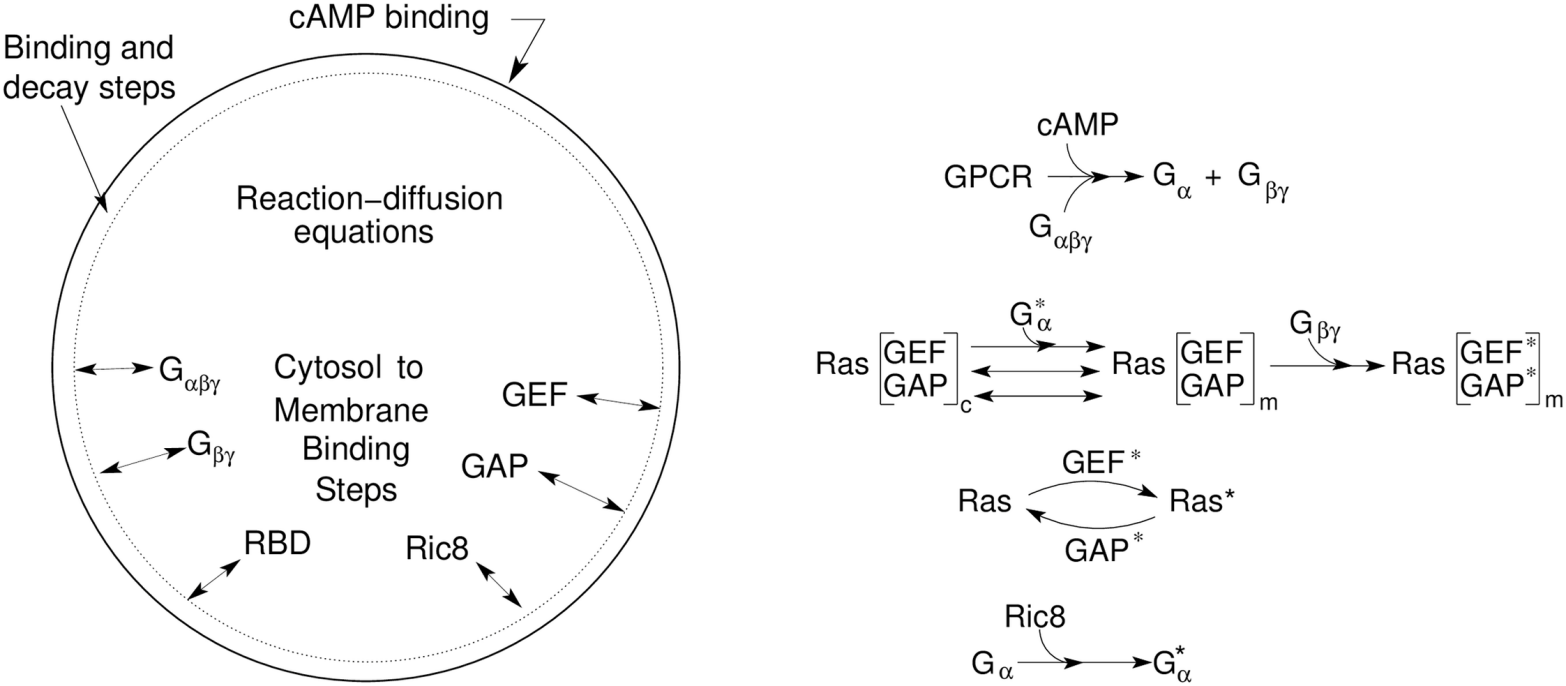
A schematic of the major processes in the model (left) and the primary steps in the network (right).

In light of the restriction to LatA-treated cells, the backbone of the chemotactic pathway activated in response to changes in extracellular cAMP is Δ cAMP →Δ GPCR occupation →Δ G_*αβγ*_ activation →Δ Ras activity. We describe this pathway in terms of three modules: the GPCR surface receptors cAR1-4, ${\text{G}}_{\alpha_{2}\beta \gamma }$ and Ras, as illustrated in Fig 1.

#### The GPCR surface receptor

The first step in Dicty chemotaxis is binding of cAMP to the G-protein coupled receptors (GPCRs) cAR1-4. The four receptor types, which have different affinities, are expressed sequentially throughout the developmental transition from a unicellular to a multicellular organism. Switching of receptor subtypes enable Dicty to response to changing chemoattractant concentrations in a wide range and hence program morphogenesis appropriately [32–34]. Lateral diffusion of the receptors has been suggested by observing green fluorescent protein (GFP)-tagged receptors in Dicty. The diffusion coefficient measured from the movements of individual receptors is about 2.7 ±1.1 × 10^−10^ *cm*^2^ *s*^−1^ [35], which is small at the scale of the cell size, but which could be locally significant on the scale of structures such as blebs.

It is well established in mammalian cells that ligand-induced phosphorylation of GPCRs leads to recruitment of arrestin family proteins, which uncouple receptors from downstream G proteins [36, 37]. cAR1 is phosphorylated at multiple cytoplasmic residues upon chemoattractant stimulation [38, 39], which is correlated to agonist-induced loss of ligand binding [40]. The functional consequence of receptor phosphorylation for chemotaxis has not been fully addressed, but it is known that receptor phosphorylation is not essential for chemotaxis or termination of G-protein-mediated responses [41], and since there is no evidence that receptor phosphorylation affects Ras we do not include it.

#### The G protein module

The G proteins function as transducers of extracellular cAMP signals for gradient sensing, since studies show that localized responses such as Ras activation occur upstream of PI3-kinase activity and downstream of G protein activity [18]. There are 11 G_*α*_ subunits and a single G_*β*_ and G_*γ*_ subunit in Dicty [42]. This single G_*βγ*_ subunit is essential for chemotactic signal transduction since $g_{\beta }^{-}$ cells do not show any Ras activation [19] and do not chemotact [14]. The primary *G*_*α*_ subunit in chemotaxis is ${\text{G}}_{\alpha_{2}}$, since $g_{\alpha_{2}}^{-}$ cells lack an essential component of the response to cAMP, as described later [19, 31].

Ligand binding to the GPCR catalyzes the exchange of GTP for GDP on the G_*α*_ subunit, causing the dissociation of activated ${\text{G}}_{\alpha }^{*}$ subunits and G_*βγ*_ subunits. Hydrolysis of GTP in ${\text{G}}_{\alpha }^{*}$ induces reassociation, which reduces active G-protein subunits when the chemoattractant is removed [8, 30]. By monitoring fluorescence resonance energy transfer (FRET) between the *α* and *β* subunits, the membrane dynamics of the heterotrimer prior to and after simulation in Dicty has been visualized [43, 44], and it has been shown that G protein activation reaches a persistent dose-dependent steady-state level during continuous stimulation, *i.e.,* no adaptation occurs at this level [25, 43].

These and other studies show that ${\text{G}}_{\alpha_{2}\beta \gamma }$ and G_*βγ*_ subunits cycle between the cytosol and the plasma membrane, while the activated G_*α*_ probably remains membrane-bound [17, 45]. Moreover, although asymmetric distributions of G_*βγ*_ subunits are observed in highly polarized Dicty, in LatA-treated cells G_*β*γ_ is uniformly distributed along the plasma membrane and within the cytosol in the presence of a cAMP gradient [46], which further suggests that G_*βγ*_ is also cycling between the membrane and the cytosol. Finally, it is reported that Dicty ‘resistant to inhibitors of cholinesterase 8’ (Ric8) is a nonreceptor GEF for ${\text{G}}_{\alpha_{2}}$, which converts $G_{\alpha_{2}}$ GDP into the activated $G_{\alpha_{2}}$-GTP form [31]. The regulation of Ric8 activity is currently not clear, but its role as a GEF probably involves binding of ${\text{G}}_{\alpha_{2}}$ to Ric8 [31].

#### Ras GTPases

Ras belongs to the family of small G proteins that function as molecular switches to control a wide variety of important cellular functions. In Dicty, there are 5 characterized isoforms: RasS, RasD, RasB, RasC, and RasG encoded by 14 Ras family genes [8]. RasC and RasG proteins appear to be particularly important for chemotaxis, of which RasG is the key Ras protein in the regulation of cAMP-mediated chemotaxis [19].

In the chemotactic backbone the Ras module provides a link between G proteins and downstream pathways. Ras proteins exist in an inactive GDP-bound state and an active GTP-bound state, and conversion between these is regulated by RasGEFs and GTPase activating proteins (RasGAPs). RasGEFs catalyze the exchange of GDP for GTP, thereby activating Ras, whereas RasGAPs stimulate the GTPase activity, converting the protein into the inactive GDP-bound form. Regulation of Ras conversion by GEF and GAP includes protein-protein or protein-lipid interactions, binding of second messengers, and post-translational modifications that induce one or more of the following changes: translocation to a specific compartment of the cell, release from autoinhibition, and the induction of allosteric changes in the catalytic domain [47]. Several methods have been developed to detect Ras protein and small GTPase activation [48], but the dynamics of Ras are usually monitored by the translocation of a tagged Ras-binding domain (RBD) peptide. The RBD of Raf1 only binds to the activated Ras-GTP, which enables localized visualization of Ras activity. The response of activated Ras in Dicty shows near-perfect adaptation, although some deviation from perfect adaptation can be observed [21].

The full set of reactions and translocation steps are given in Table 1, wherein reactions and translocations are labeled as $R_{\text{s}}$ and $J_{\text{s}}$, respectively, and the corresponding rate laws, which are derived by assuming mass-action kinetics for all steps, are denoted by r_*s*_ and *j*_*s*_, respectively. In reality the translocation of a substance between the cytosol and the membrane takes place within a layer near the membrane, but we treat this as a surface reaction. Moreover, we assume that complex formation is always fast and that a negligible amount of the factors is in the complex form, so that the conversion rate of the substrate is proportional to the product of regulator and substrate densities (see section Reaction rates in S1 Text), unless otherwise indicated. To eliminate the effects of intrinsic polarity and investigate the system dynamics without feedback from the cytoskeleton, we assume that the cells are pretreated with LatA, in which case they lose polarity and become rounded and immobile.

**Table 1:** Kinetics and rates of the reactions.

Label and Description	Kinetics	Rate	Reference
① ligand binding	$R_{1}:cAMP+R\underset{{\text{k}}_{1}^{-}}{\overset{{\text{k}}_{1}^{+}}{⇌}}R^{*}$	${\text{k}}_{1}^{+}$,${\text{k}}_{1}^{-}$	[35, 43]
② ${\text{G}}_{\alpha_{2}\beta \gamma }$ cycling	$J_{1}:G_{\alpha \beta \gamma ,m}\underset{{\text{h}}_{2}}{\overset{{\text{h}}_{1}}{⇌}}G_{\alpha \beta \gamma ,c}$	h_1_, h_2_	[17, 45]
③ ${\text{G}}_{\alpha_{2}\beta \gamma }$ dissociation	$R_{2}:G_{\alpha \beta \gamma ,m}+R^{*}\overset{{\text{k}}_{2}}{\to }G_{\alpha }^{*}+G_{\beta \gamma ,m}+R^{*}$	k_2_	[8, 30]
④ G_*βγ*_ cycling	$J_{2}:G_{\beta \gamma ,m}\underset{{\text{h}}_{4}}{\overset{{\text{h}}_{3}}{⇌}}G_{\beta \gamma ,c}$	h_3_, h_4_	[17, 45]
⑤ GTPase of ${\text{G}}_{\alpha }^{*}$	$R_{3}:G_{\alpha }^{*}\overset{{\text{k}}_{3}}{\to }G_{\alpha }$	k_3_	[8, 30]
⑥ Ric8 cycling	$J_{3}:Ric8_{m}\underset{{\text{h}}_{6}}{\overset{{\text{h}}_{5}}{⇌}}Ric8_{c}$	h_5_, h_6_	[31]
⑦ Promoted Ric8 cycling	$J_{4}:Ric8_{c}+G_{\alpha }^{*}\overset{{\text{h}}_{7}}{\to }Ric8_{m}+G_{\alpha }^{*}$	h_7_	Assumed
⑧ Ric8 activation	$R_{4}:Ric8_{m}+G_{\beta \gamma ,m}\overset{{\text{k}}_{4}}{\to }Ric8^{*}+G_{\beta \gamma ,m}$	k_4_	Assumed
⑨ G_*α*_ reactivation	$R_{5}:Ric8^{*}+G_{\alpha }\overset{{\text{k}}_{5}}{\to }Ric8^{*}+G_{\alpha }^{*}$	k_5_	[31]
⑩ Ric8 inactivation	$R_{6}:Ric8^{*}\overset{{\text{k}}_{6}}{\to }Ric8_{m}$	k_6_	Assumed
⑪ ${\text{G}}_{\alpha_{2}\beta \gamma }$ reassociation	$R_{7}:G_{\alpha }+G_{\beta \gamma ,m}\overset{{\text{k}}_{7}}{\to }G_{\alpha \beta \gamma ,m}$	k_7_	[8, 30]
⑫ RasGEF cycling	$J_{5}:RasGEF_{m}\underset{{\text{h}}_{9}}{\overset{{\text{h}}_{8}}{⇌}}RasGEF_{c}$	h_8_, h_9_	[49]
⑬ Promoted RasGEF cycling	$J_{6}:RasGEF_{c}+G_{\alpha }^{*}\overset{{\text{h}}_{10}}{\to }RasGEF_{m}+G_{\alpha }^{*}$	h_10_	[50–53]
⑭ RasGAP cycling	$J_{7}:RasGAP_{m}\underset{{\text{h}}_{12}}{\overset{{\text{h}}_{11}}{⇌}}RasGAP_{c}$	h_11_, h_12_	[47]
⑮ RasGEF activation	$R_{8}:RasGEF_{m}+G_{\beta \gamma ,m}\overset{{\text{k}}_{8}}{\to }RasGEF^{*}+G_{\beta \gamma ,m}$	k_8_	[19, 28]
⑯ RasGEF inactivation	$R_{9}:RasGEF^{*}\overset{{\text{k}}_{9}}{\to }RasGEF_{m}$	k_9_	[19, 28]
⑰ RasGAP activation	$R_{10}:RasGAP_{m}+G_{\beta \gamma ,m}\overset{{\text{k}}_{10}}{\to }RasGAP^{*}+G_{\beta \gamma ,m}$	k_10_	[19, 28]
⑱ RasGAP inactivation	$R_{11}:RasGAP^{*}\overset{{\text{k}}_{11}}{\to }RasGAP_{m}$	k_11_	[19, 28]
⑲ Ras activation	$R_{12}:RasGEF^{*}+Ras\overset{{\text{k}}_{12}}{\to }RasGEF^{*}+Ras^{*}$	k_12_	[19, 28]
⑳ Ras inactivation	$R_{13}:RasGAP^{*}+Ras^{*}\overset{{\text{k}}_{13}}{\to }RasGAP^{*}+Ras$	k_13_	[19, 28]
㉑ Spontaneous Ras activation	$R_{14}:Ras\overset{{\text{k}}_{14}}{\to }Ras^{*}$	k_14_	[19, 28]
㉒ Spontaneous Ras inactivation	$R_{15}:Ras^{*}\overset{{\text{k}}_{15}}{\to }Ras$	k_15_	[19, 28]
㉓ RBD cycling	$J_{8}:RBD_{m}\underset{{\text{h}}_{14}}{\overset{{\text{h}}_{13}}{⇌}}RBD_{c}$	h_13_, h_14_	[19, 28]
㉔ Promoted RBD cycling	$J_{9}:RBD_{c}+Ras^{*}\overset{{\text{h}}_{15}}{\to }RBD_{m}+Ras^{*}$	h_15_	[19, 28]

The applicable conservation conditions on the various species are implicit in the evolution equations, which are given in detail in the Materials and Methods section. For simplicity, we model a cell as a 3D sphere centered at the origin, of radius 5*μm* [46]. The initial condition for the system is the steady state in a very small concentration (0.001*pM*) of cAMP in the extracellular space. The system is solved numerically using a finite element discretization in space and backward differentiation for the time stepping, implemented in the COMSOL multiphysics package. In the following sections, we exhibit the cell response under various stimulation protocols, and for notational simplicity, we use *G*_*α*_ in place of *G*_*α*2_ when necessary. Some of the results that will be discussed are as follows.

- Under uniform stimuli –
  - The transient response
  - Imperfect adaptation
  - The response of $g_{\alpha_{2}}^{-}$ and *ric*8^−^ cells.
- Under graded stumuli –
  - The origin of the biphasic Ras activation and the necessity of ‘activator’ diffusion
  - How the magnitude of gradient amplification depends on the cAMP amplitude and gradient
  - The response of $g_{\alpha_{2}}^{-}$ and *ric*8^−^ cells in a gradient.
  - The ‘back-of-the-wave’ problem.

Remarks.

- The affinities of four receptors in Dicty have been measured in various conditions [33] and receptors have the ability of switching their affinity between high affinity and low affinity [34]. To avoid modeling all four receptors, we model the binding with an averaged binding affinity and dissociation rate
- The intrinsic guanosine triphosphatase (GTPase) activity of ${\text{G}}_{\alpha }^{*}$ hydrolyses the bound GTP on the plasma membrane, whose rate can vary depending on the influence of regulator of G protein signalling (RGS) proteins [54, 55]
- The regulation of Ric8 activity is still not clear. We assume a translocation-activation mechanism here: Ric8 translocation can be promoted by ${\text{G}}_{\alpha }^{*}$ (The scenarios of G_*α*_ promotion and no promotion (g*α*-null) are also investigated); Ric8 is activated by G_*βγ*, *m*_ (The scenario of translocation-only is investigated, in which case *Ric*8_*m*_ converts G_*α*_ directly into ${\text{G}}_{\alpha }^{*}$). The simulations suggest that this activation is not essential to induce symmetry breaking
- An inactivation is introduced to balance the Ric8 activation step. In the translocation-only scenario, this step is eliminated

## Results

### The response under a uniform stimulus

#### *G*_*α*_ dynamics

**Fig 2:**
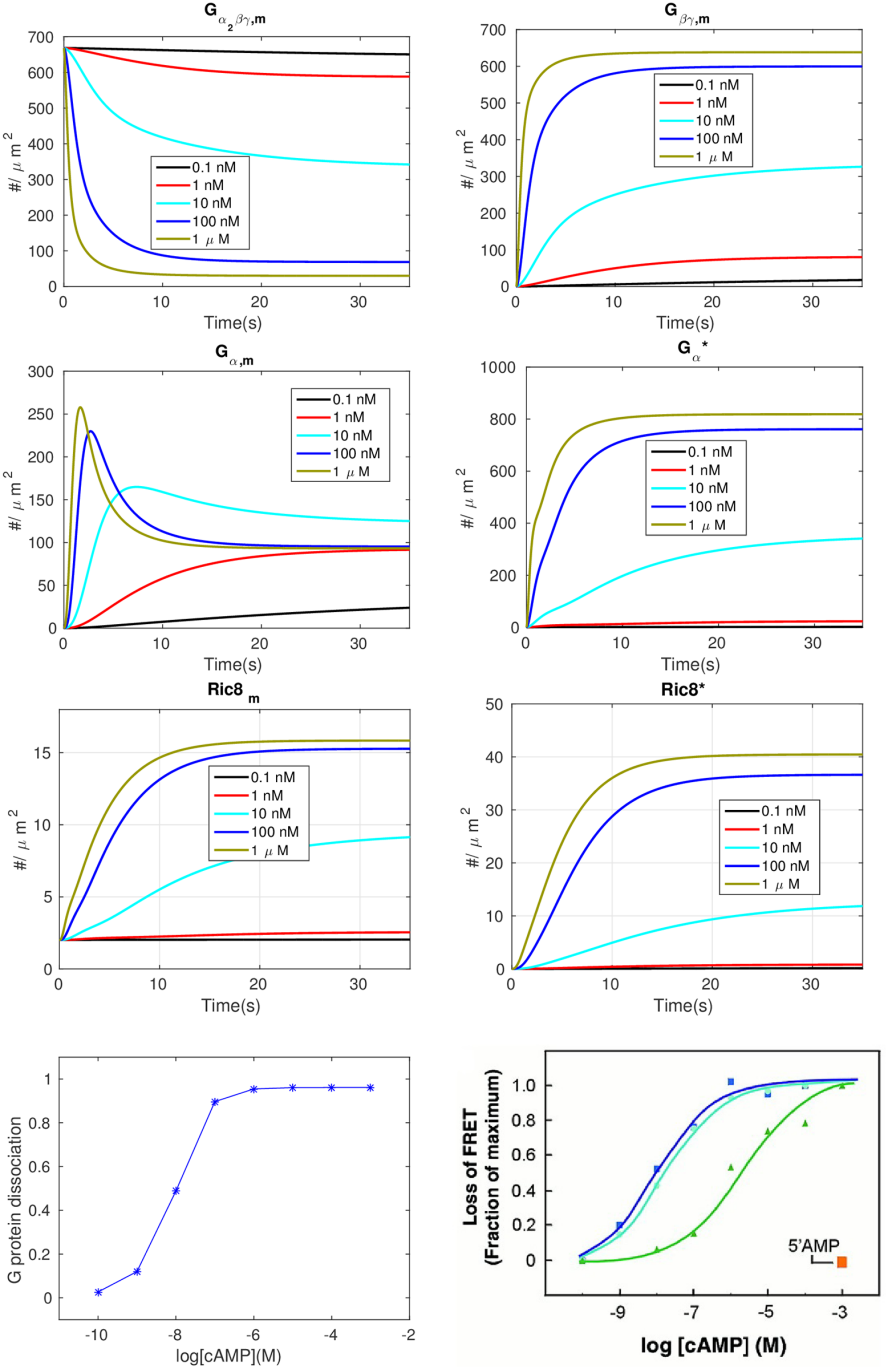
The time course of various components under different levels of uniform stimuli. *First row*—${\text{G}}_{\alpha_{2}\beta \gamma }$ and G_*β*γ_: *Second row*—*G*_α_ and $G_{\alpha }^{*}$; *Third row*:—Ric8 and *Ric*8*. *Fourth row*—The dose dependent dissociation at steady state. Left: model prediction; *Right*: Dose-response curves for cAMP (dark blue), 2’-dcAMP(light blue), and 8-Br cAMP (green), and 5’ AMP (orange) from [43] with permission.

As previously noted, ${\text{G}}_{\alpha_{2}\beta \gamma }$ dissociates rapidly upon addition of chemoattractant and ${\text{G}}_{\alpha }^{*}$ and G_*β*γ_ reach a dose-dependent steady-state level during continuous stimulation, even though downstream responses subside [43]. The computed dose-dependent time evolutions of ${\text{G}}_{\alpha_{2}\beta \gamma }$ and G_*β*γ_ are shown in the first row of Fig 2. Under a spatially-uniform stimulus the concentration of ${\text{G}}_{\alpha_{2}\beta \gamma }$ decreases due to dissociation induced by cAMP-bound cAR, the concentration of G_*β*γ_ subunits increases, and the steady state level of each is dose-dependent. The time to reach a steady state level decreases as the cAMP increases, and at 1*μM* cAMP the dissociation is stabilized within 5 seconds of activation, which is consistent with results in [43].

The dynamics of the *G*_*α*_ subunits are shown in the second row of Fig 2. As shown in the right panel, *G*_*α*_ is activated in a dose-dependent persistent manner similar to G_*β*γ_, but $G_{\alpha }^{*}$ reaches steady state more slowly than G_*β*γ_ and the steady state concentration is higher at a given cAMP stimulus, because both forms of *G*_*α*_ remain membrane-bound. Surprisingly, the simulation shows that *G*_*α*_ exhibits a biphasic response when the cAMP concentration is above a certain threshold. When the cAMP concentration is lower than 1 nM the *G*_*α*_ concentration increases to the steady state monotonically, but if the cAMP concentration is greater than 10 nM the *G*_*α*_ concentration shows an initial overshoot and then decreases to the steady state, which illustrates the kinetic diversity of G protein signalling [56]. Furthermore, unlike the response of G_*β*γ_ and $G_{\alpha }^{*}$, for which a higher concentration of cAMP produces a higher steady state levels of subunits, for *G*_*α*_ there is an optimal cAMP concentration at which the steady state level of *G*_*α*_ is maximized.

In light of our assumption that Ric8 is localized on the membrane by $G_{\alpha }^{*}$ and activated by G_*β*γ_, it follows that the model predicts that Ric8 activation is also nonadaptative, as demonstrated in the third row of Fig 2. In the fourth row of Fig 2 we show the comparison of dose-dependent ${\text{G}}_{\alpha_{2}\beta \gamma }$ dissociation between the observations in [43] and our model prediction. One sees that the predictions matches the experimental data and both show that dissociation of ${\text{G}}_{\alpha_{2}\beta \gamma }$ is saturated at 1 *μM* cAMP.

#### Imperfect adaptation at the level of Ras

**Fig 3:**
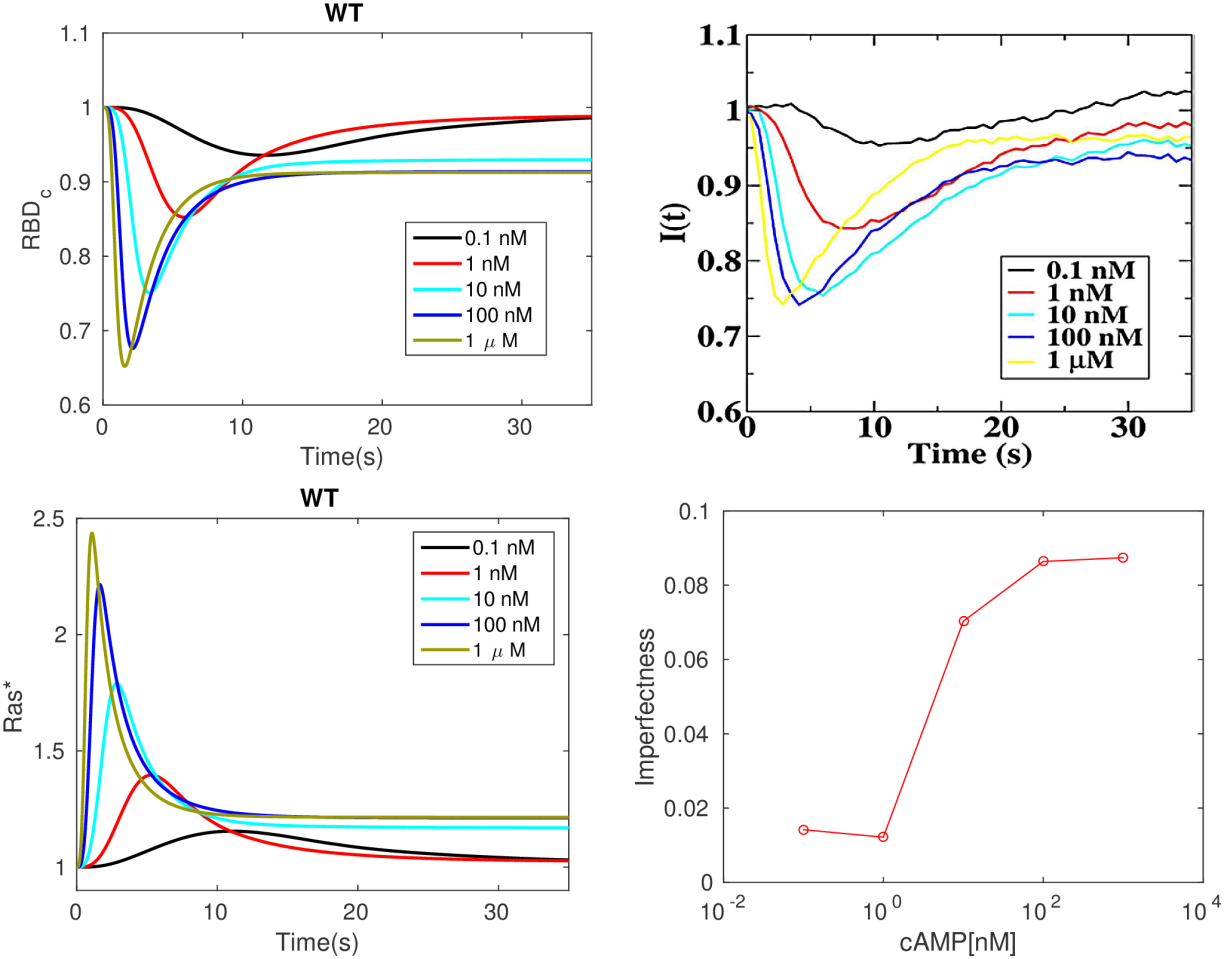
Transient Ras activation and imperfection of Ras adaptation. *Top*: Uniform stimulation causes a transient decrease in the average cytosolic concentration of RBD. WT signifies a wild type cell. *Left*: simulation; *Right*: experimental measurements from [28]. *Bottom*: transient Ras activation and imperfection of Ras activation, computed as the relative difference between the steady state Ras level under stimulus and without.

It is suggested in [28] that adaptation of Ras activity is due to incoherent feedforward control via activation and inactivation of Ras by RasGEF and RasGAP, resp. Ras activation is monitored via membrane localization of RBD, which diffuses freely in the cytosol and is localized at the membrane by binding to active Ras. The comparison between the experimental results for LatA-treated cells and the model predictions are shown in the top row of Fig 3. One sees that the model captures several basic aspects seen in the observed Ras activation.

- After an increase in cAMP, RBD rapidly translocates to the membrane and binds to Ras*—whose dynamics are shown in bottom left of Fig 3—reaching a maximum in a few seconds. This is followed by a more gradual return to the cytosol, where RBD returns to approximately its basal level.
- The maximum response increases with increasing concentrations and saturates at about 1 *μM* cAMP.
- The time to the peak of the Ras* response decreases with increasing cAMP concentration.

While perfect adaptation has been confirmed in bacterial gradient sensing [57], the experimental evidence in eukaryotes is mixed and sometimes suggests that only partial adaptation takes place [58–60]. Although it was claimed that the adaptation is near-perfect in Dicty [28], the experimental results in the top right panel of Fig 3 show that it is not. Imperfect activation is also reported in [21], and the degree of imperfection is quantified at various cAMP stimulus levels there. The model also predicts imperfect adaptation, as shown in the top left panel of Fig 3, and the deviation from perfect adaptation is shown in the bottom right panel of that figure. Both the simulations and experimental measurements show that the deviation from perfect adaptation increases with the level of stimulation and saturates at about 100 nM, and in both cases the relative deviation from perfect adaptation does not exceed 0.1.

It is suggested in [28] that the local activator and global inhibitor of a LEGI model are RasGEF and RasGAP, respectively, and that only RasGAP diffuses in the cytosol. Our model differs from this at the level of Ras activation by incorporating a diffusion-translocation-activation mechanism for both RasGEF and RasGAP. In other words, RasGEF and RasGAP are both globally supplied through diffusion—with the same diffusion coefficients—while only localization of RasGEF is increased by the locally constrained $G_{\alpha }^{*}$, resulting in stronger persistent RasGEF activation. Consequently, RasGAP activation cannot offset this, even under spatially-uniform stimuli, thereby inducing imperfect adaptation (see section Imperfect adaptation in S1 text for analysis).

#### Refractoriness induced by subtle temporal regulation of RasGEF and RasGAP activation

**Fig 4:**
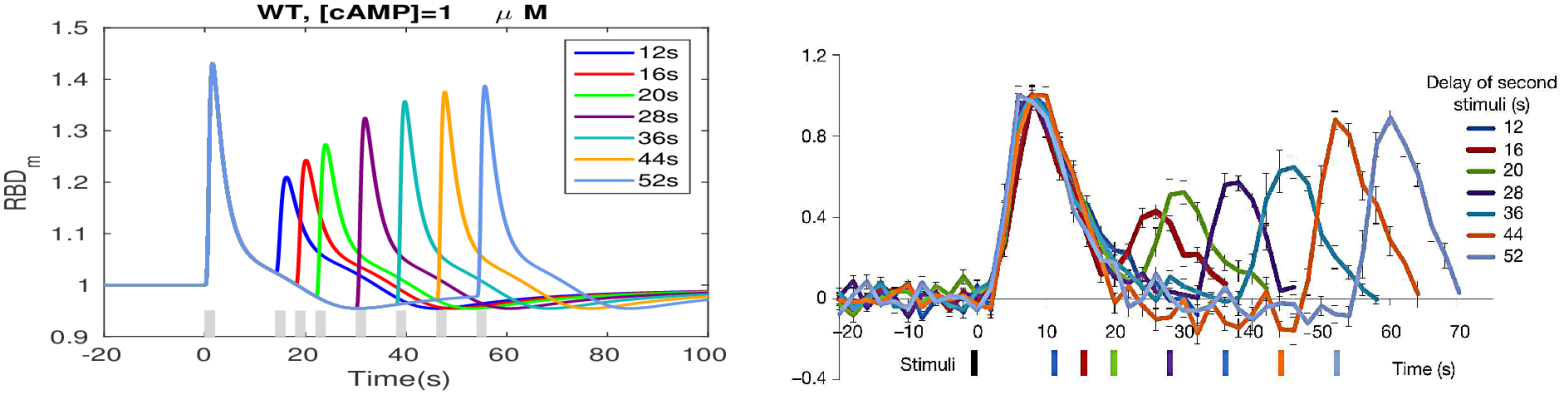
Refractoriness under uniform stimuli. *Left*: Simulation. The gray bar indicates the duration of the stimulus; *Right*: experimental results from [20]. The black bar indicates the first stimulus. The other bars are color-coded to show the delay. All values are normalized to the peak of the first response.

Refractoriness, which is a characteristic of excitable systems, has been reported for Dicty in [20]. When two brief large stimuli are applied to the same cell, the response to the second stimulus depends on the interval between it and the first, as shown in Fig 4 (right), which suggests the existence of a refractory period. We repeated this experiment computationally by applying 1 *μM* cAMP stimuli for 2 sec (The model exhibits a maximal response to this short saturating stimuli, see Fig. A in S1 Text) separated by increasing intervals. As shown in Fig 4 (left), refractoriness is observed and the decrease in the second response decreases as the separation time increases, consistent with the experimental observations. Moreover, the peak response with a 52s delay is still weaker than the first response, both in simulation and experimental measurements, probably due to the fact that Ras does not adapt perfectly.

**Fig 5:**
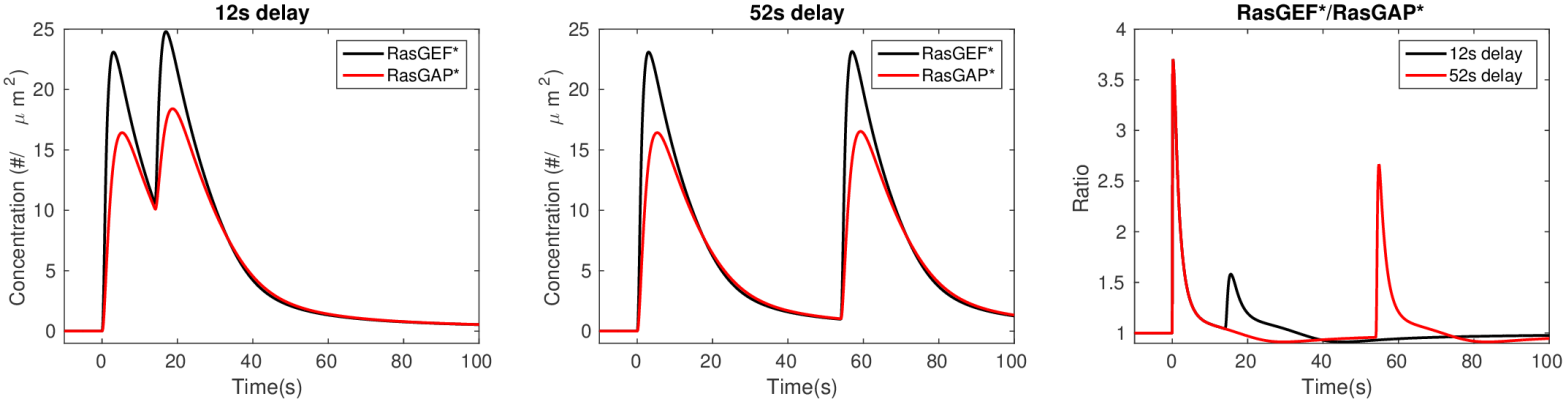
The time courses of RasGEF* and RasGAP* for a 12s delay (left), a 52s delay (center) and RasGEF* /RasGAP* ratio for both time intervals (right). Note that the ratio reaches a peak before the two factors reach their peaks.

As to the refractory period, note that under large stimuli large fractions of RasGEF and RasGAP are activated, and when the duration between the stimuli is too short, neither RasGEF nor RasGAP can return to prestimulus levels, as shown by comparison of the left and center panels of Fig 5. As a result, the peak ratio of activated RasGEF and RasGAP decreases for short inter-stimulus intervals as compared with long intervals, as shown in the right panel of Fig 5. Note that the ratio for a 12 sec interval in Fig 5 differs from the corresponding RBD ratio in Fig 4 because there is a basal, unstimulated translocation of RBD to the membrane. This indicates that refractoriness is stimulus level dependent. The refractory periods for non-saturating cAMP stimuli are reported in S1 Text (see Fig. B in S1 Text).

#### $g_{\alpha_{2}}$-null and ric8-null cells

In wild type cells, most of the G_*βγ*_ comes from $G_{\alpha_{2}\beta \gamma }$ under cAMP stimulation, but G_*βγ*_ can also be released from other G proteins [19]. In the absence of quantitative data on amounts and affinities of different G proteins, we simulated the wild type cells assuming that all G proteins dissociate and reassociate at the same rate, and that ${\text{G}}_{\alpha_{2}^{*}}$ and G_*βγ*_ are produced in a 1-1 ratio. However, some Ras response is still observed in in $g_{\alpha_{2}}$-null cells, and to simulate these we assume that G_*βγ*_ is released from $G_{\alpha_{x}\beta \gamma }$ and that the total amount of *G*_*βγ*_ is the same as in WT cells. Of course the total amount of $G_{\alpha_{x}\beta \gamma }$ should be less than in WT cells if other forms are not overexpressed in ${\text{G}}_{\alpha_{2}}$-null cells, and therefore we also did simulations in which the total amount of G_*βγ*_ was reduced 90% in *g*_*α*_2__-null cells compared to WT levels. In that case the peak activation of Ras is slightly weaker, but the overall Ras activity does not change much because it is controlled by the ratio of RasGEF to RasGAP.

**Fig 6:**
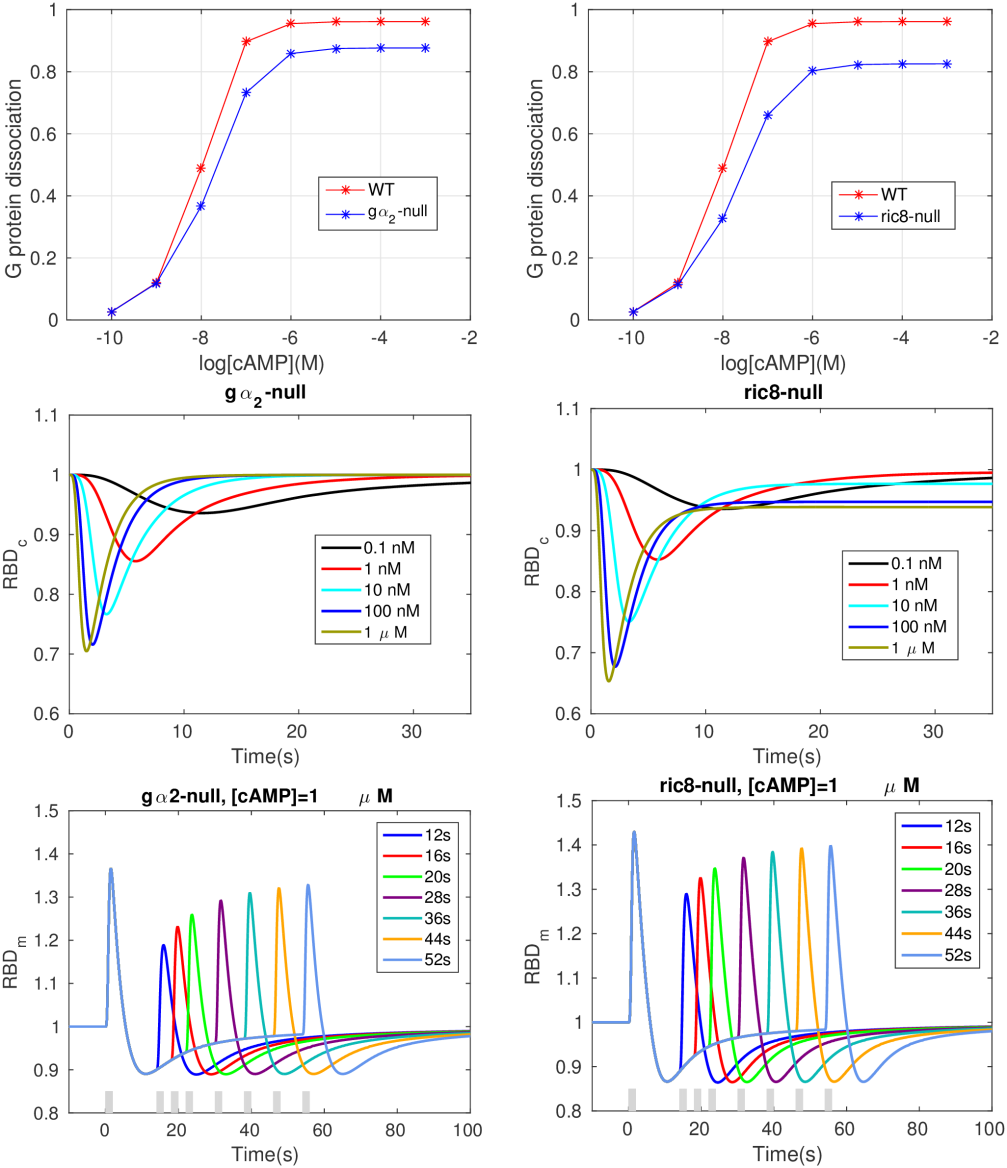
Various cell responses in the simulated $g_{\alpha_{2}}$-null cell and ric8-null cells. *Top*: Dose dependent dissociation at steady state. *Middle*: Time course of RBD dynamics. *Bottom*: refractoriness.

More precisely, we assume that when cAMP binds to a receptor, $G_{\alpha_{x}\beta \gamma }$ dissociates at the same rate as in WT cells, and that Ric8 regulates ${\text{G}}_{\alpha_{x}}^{*}$ hydrolysis through spontaneous membrane localization and G_*βγ*_-mediated activation. ${\text{G}}_{\alpha_{x}}^{*}$ and ${\text{G}}_{\alpha_{x}}$ only affect G protein cycling and no other components in the network. Specifically, ⑦ and ⑬ in Table 1 are disabled in $g_{\alpha_{2}}$-null cells and ⑥–⑩ are disabled in ric8-null cells. As shown in the first row of Fig 6, ${\text{G}}_{\alpha_{2}\beta \gamma }$ dissociation decreases in both $g_{\alpha_{2}}$-null cell and ric8-null cells. Note that since Ric8 translocation is not enhanced in $g_{\alpha_{2}}$-null cells, $G_{\alpha_{2}}$ is reactivated at a lower rate $G_{\alpha_{2}}$ in wild type cells. Consequently, $G_{\alpha_{x}\beta \gamma }$ cycling dynamics is altered and $G_{\alpha_{x}\beta \gamma }$ dissociation decreases. Similarly, *ric*8-null cells also show decreased ${\text{G}}_{\alpha_{2}\beta \gamma }$ dissociation because there is no Ric8 binding to G_*β*γ_.

The RBD responses are shown in the second row of Fig 6. Adaptation is perfect for any physiologically-reasonable cAMP stimulus in $g_{\alpha_{2}}$-null cells, and the rate of Ras activation is initially the same as in WT cells, but the RBD response is less pronounced (*cf.* Fig 3). RasGEF activation is weaker in $g_{\alpha_{2}}$-null cells due to the absence of ${\text{G}}_{\alpha_{2}}^{*}$-promoted RasGEF recruitment, and the incoherent feedforward circuit in the model guarantees that the activation of RasGEF and RasGAP are perfectly balanced. Hence perfect adaptation occurs and the maximum response is reduced compared to that in WT cells, which agrees with the results in [31]. For ric8-null cells, one sees that *ric*8-null cells still exhibit imperfect adaptation, since $G_{\alpha_{2}}^{*}$-promoted RasGEF translocation still occurs, but the imperfectness is reduced due to the fact that there is no Ric8 available to reactivate $G_{\alpha_{2}}$. Simulations show that *ric*8-null cells with a reduced $G_{\alpha_{2}}^{*}$-GTP hydrolysis rate approximately resemble the WT behaviors (not shown).

The bottom row shows that the refractory response is still observable in both mutant cells, but the dependence on the time interval is less sensitive compared with WT cells. For $g_{\alpha_{2}}$-null cells, the change in the RBD response is less than 10% (from ∼ 1.11 to ∼ 1.2) when the interval ranges from 12 to 52 seconds, compared with a 20% change (from ∼ 1.2 to ∼ 1.4) in WT cells (Fig 4). There is less than a 10% difference in maximum response between a 12s interval and a 52s interval (right panel, from ∼ 1.3 to ∼ 1.38) for ric8-null cells.

### The response under a graded stimulus

Next we investigate how cells respond to a linear cAMP gradient along the x-axis, which we define as follows.

$$C\left(x,y,z\right)=\frac{\Delta C}{10}\cdot \left(x-x_{r}\right)+C_{r}$$

where *C*(*x*, *y*, *z*) is the cAMP concentration on the membrane at $\left(x,y,z\right)\in S_{5}^{2}$ (a sphere of radius 5), Δ*C* ≡ *C*_*f*_ − *C*_*r*_, and subscripts *f* and *r* denote the points (5,0,0) (the ‘front’) and (-5,0,0) (the ‘rear’).

#### Biphasic Ras activation in LatA-treated cells

It was shown in [19] that spatially-localized stimuli lead to three phases of Ras activation. In the first, which is transient, Ras isactivated on the entire membrane, and this phase requires G_*β*γ_ and exists in $g_{\alpha_{2}}$-null cells. The second phase is symmetry breaking, in that Ras is only activated at the side of the cell facing the higher cAMP concentration, and this phase requires $G_{\alpha_{2}}$. The third phase is confinement, wherein the crescent of activated Ras at the front half of the cell localizes to a small area around the high point of the gradient. The first two phases are observed in LatA-treated cells, but the third phase requires actin polymerization. Since the model is based on LatA- pretreated cells, we only test whether it exhibits the first two phases of Ras activation.

**Fig 7:**
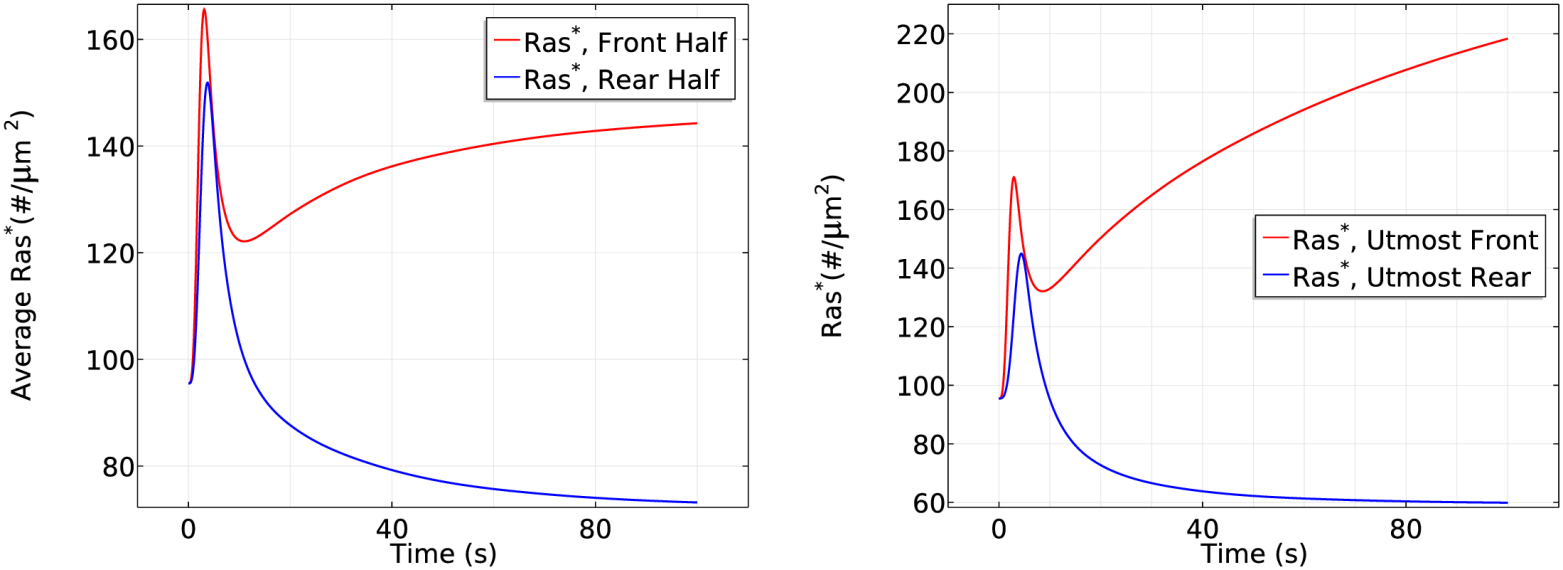
The time course of average Ras* activity in a cAMP gradient defined by *C*_*f*_ = 10 nM and *C*_*r*_ = 1nM (left) and the Ras* activity in the same gradient at *x*_*f*_ and *x*_*r*_ (right).

Fig 7 (left) shows that the initial response is transient activation of Ras on the entire boundary, which is completed in ~10 s, followed by a pronounced asymmetric activation pattern. Here and hereafter we display the average of various species at the front and rear halves of a cell because this is how experimental results are reported. In the second phase Ras is reactivated exclusively at the front half of the cell, where the peak Ras* activation is roughly twice that at the rear, which reflects the difference in receptor occupancy and G protein activation. Thus symmetry breaking occurs in this phase, which is stabilized at around *t* = 100 s. The biphasic behavior in a cAMP gradient is even more pronounced in a time plot of Ras* at the antipodal points of the gradient, as shown in the right panel of Fig 7.

The critical components that give rise to the biphasic response are several globally diffusing molecules (${\text{G}}_{\alpha_{2}\beta \gamma }$, G_*β*γ_, *Ric*8, *RasGEF* and *RasGAP*) and localized ${\text{G}}_{\alpha_{2}}^{*}$. The sequence of events following application of the graded stimulus is as follows.

**Fig 8:**
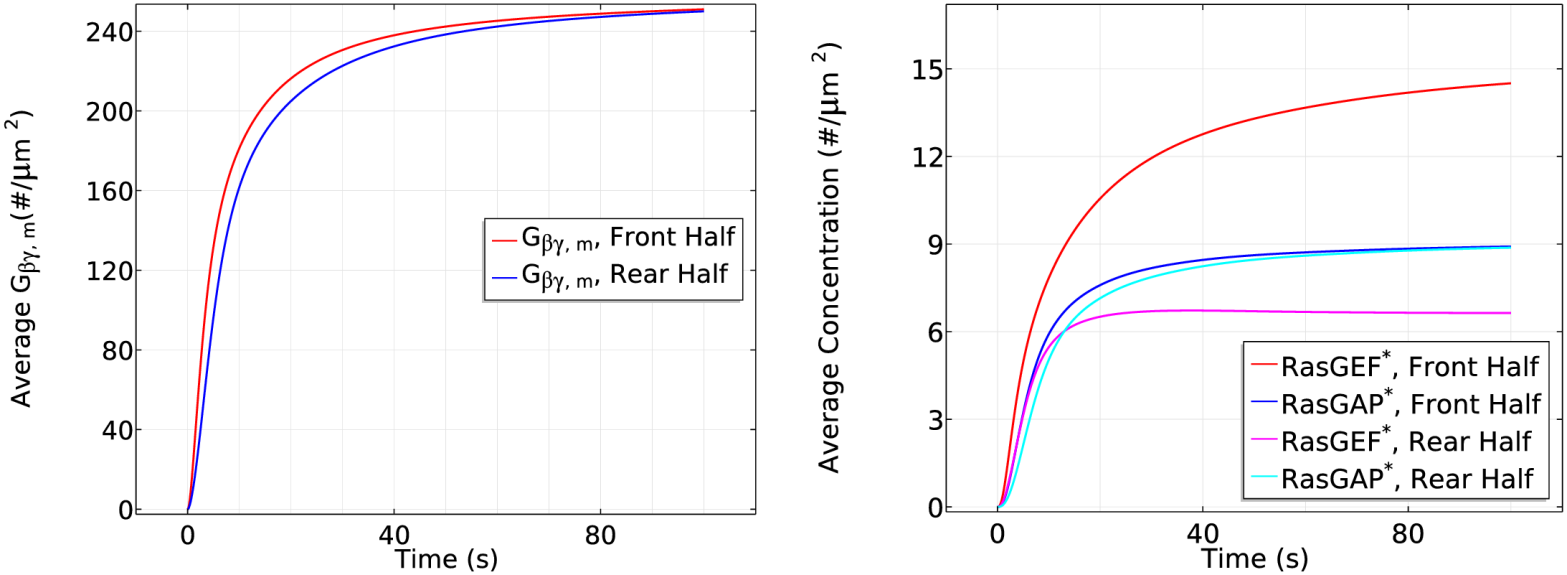
The time course of membrane G_*β*γ_ (left) and RasGEF* and RasGAP* (right) at the front and rear halves of the cell in the cAMP gradient used in Fig 7.

**Fig 9:**
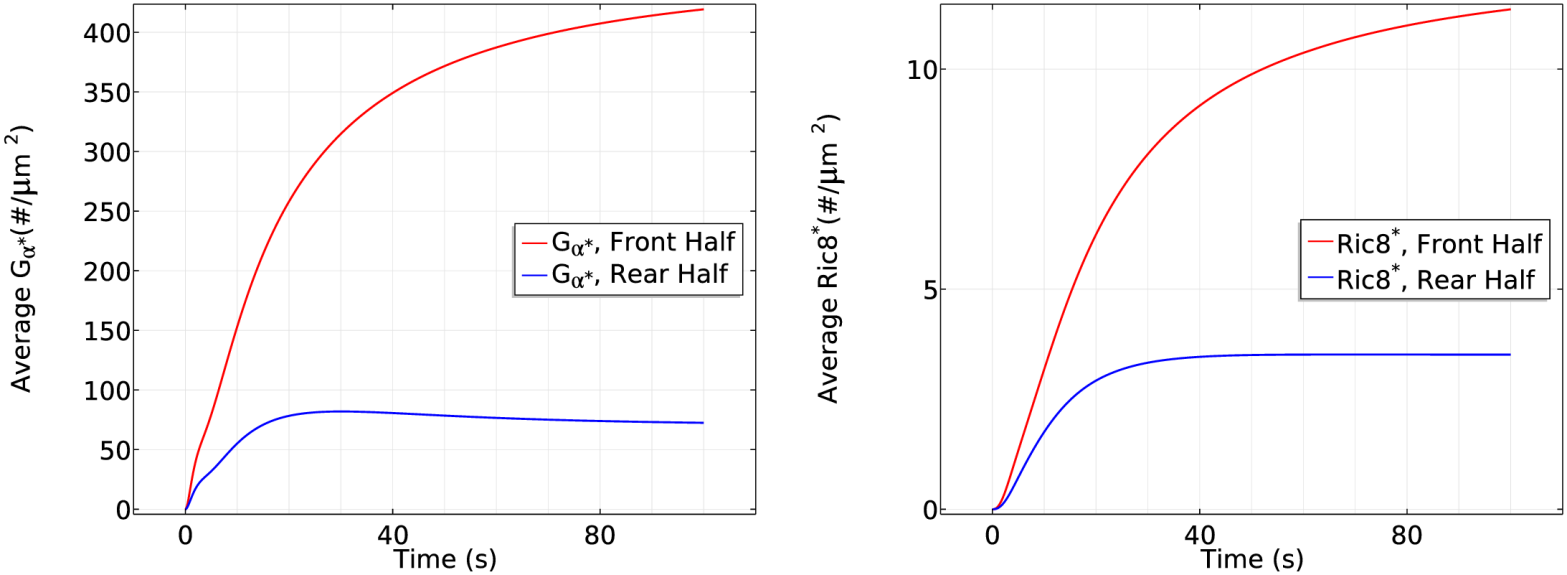
The time course of membrane $G_{\alpha }^{*}$ (left) and *Ric*8* (right) at the front half and rear half in the cAMP gradient used in Fig 7.

1. ${\text{G}}_{\alpha_{2}\beta \gamma }$ dissociation is higher at the front, resulting in more G_*βγ*_ there initially (Fig 8 (left)), but G_*β*γ_ can diffuse in the cytosol, which reduces the spatial difference. A similar difference applies to $G_{\alpha_{2}}^{*}$, but it remains membrane-bound.
2. G_*βγ*_ activates RasGEF faster than RasGAP every where (Fig 8 (right) for 0 < *t* ≤ 10*s*) which favors the activation of Ras. Because the dissociation of G_*αβγ*_ is higher at the front, Ras* increases faster there and induces a higher maximum.
3. RasGAP* activation increases on a slower time scale, resulting in a decrease of Ras* everywhere. However, the localization of $G_{\alpha_{2}}^{*}$ at the membrane enhances translocation of RasGEF from the cytosol to the membrane, and this is higher at the front than at the rear (Fig 9 (left)). This leads to higher RasGEF activation at the front (Fig 8 (right)), which offsets the Ras deactivation due to RasGAP*, and reactivation of Ras occurs.
4. At the same time, the nonuniform distribution of $G_{\alpha_{2}}^{*}$ on the membrane induces a nonuniform localization of Ric8. Although diffusion of G_*β*γ_ tends to equalize Ric8 activation, this is offset by the difference in the distribution of $G_{\alpha_{2}}^{*}$ (Fig 9 (right)). Consequently, $G_{\alpha_{2}}$ is reactivated at the front of the cell, which further promotes RasGEF localization at the front. Moreover, the asymmetrical $G_{\alpha_{2}}$ reactivation generates an asymmetrical ${\text{G}}_{\alpha_{2}\beta \gamma }$ reassociation profile—less reassociation at the front and more at the rear. As a result, diffusion of *G*_*αβγ*_ that re-associated at the rear provides a source of *G*_*αβγ*_ needed at the front, which further contributes to symmetry breaking.
5. Note that the cAMP gradient introduces a larger sink of G_*αβγ*_ and a larger G_*βγ*_ concentration at the front initially, but the diffusion of G_*αβγ*_ guarantees the continuous supply at the membrane as long as saturation is not reached. Moreover, the distribution of G_*βγ*_ is essentially uniform on the membrane and within the cytosol (Fig 8 (left)) after ~100*s*, as was reported in [46]. This eventually leads to a uniform distribution of RasGAP* at the entire cell boundary, but RasGEF* is higher at the front due to the asymmetrical recruitment of RasGEF from the cytosol. Ras activity at the rear of the cell decreases below the prestimulus level because the RasGAP* activity offsets the RasGEF* activity there.

In summary, the fast time scale of G_*β*γ_-mediated RasGEF and RasGAP activation induces the first transient Ras activation on the entire membrane, while the slow time scale of overall equilibration (redistributions due to diffusion and membrane localization) induces the delayed secondary response that produces the symmetry breaking.

#### The effects of diffusion

The results in the previous section suggest that diffusion plays an important role in inducing the biphasic response. To investigate this, we do simulations in which the diffusion coefficients of G_*β*γ_, RasGEF/GAP, ${\text{G}}_{\alpha_{2}\beta \gamma }$, and Ric8, all of which are present in the cytosol and diffuse, are individually set to 0.003*μm*^2^/*s* (10^−5^ of the normal value) and compare the Ras response with that in WT cells.

**Fig 10:**
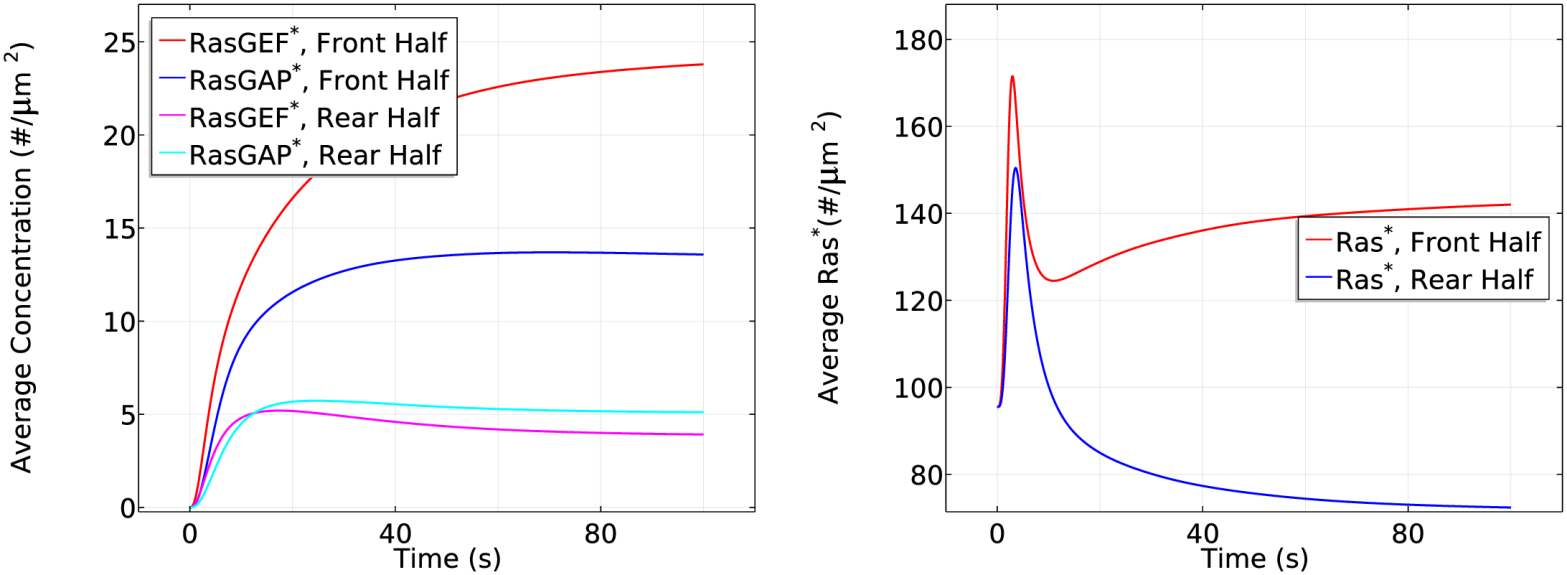
The time course of RasGEF* and RasGAP* activity (left) and Ras* activity (right) at the front and rear halves in the absence of apparent G_*βγ*_ diffusion in the same gradient as previously used.

**Fig 11:**
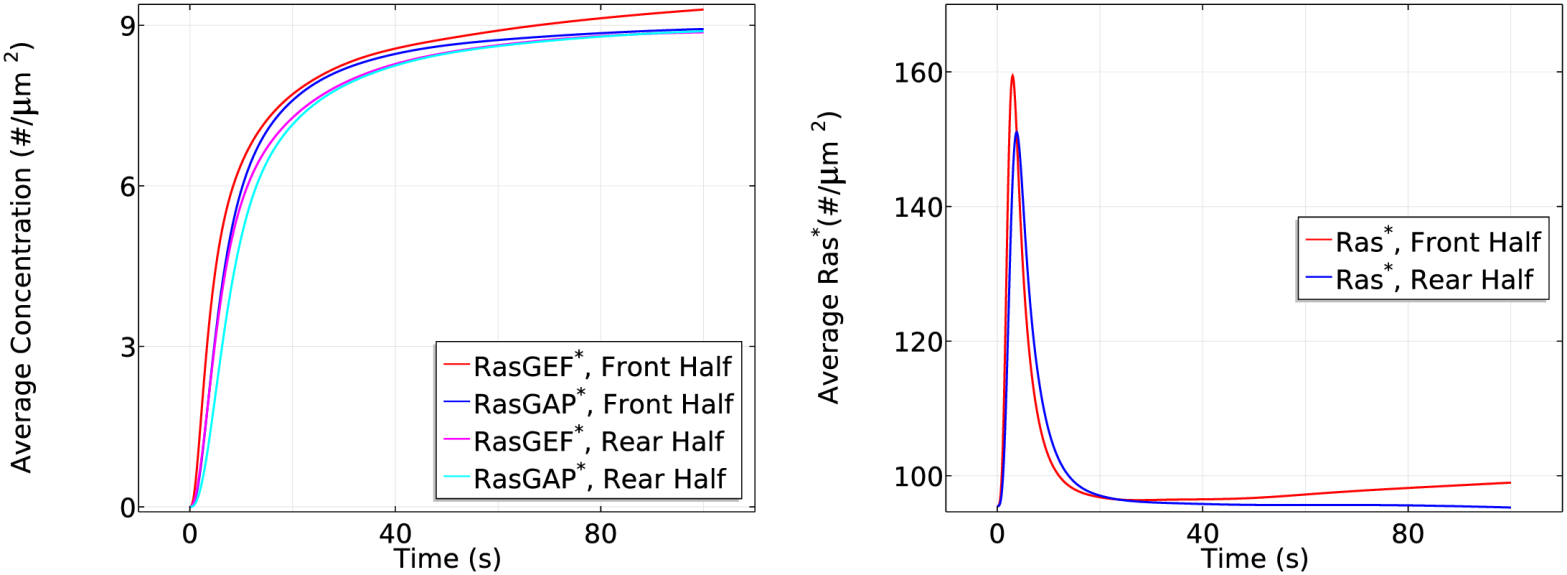
The time course of average RasGEF* and RasGAP* activity (left) and Ras* activity (right) at the front and rear halves in the absence of apparent RasGEF diffusion in the same gradient as previously used.

**Fig 12:**
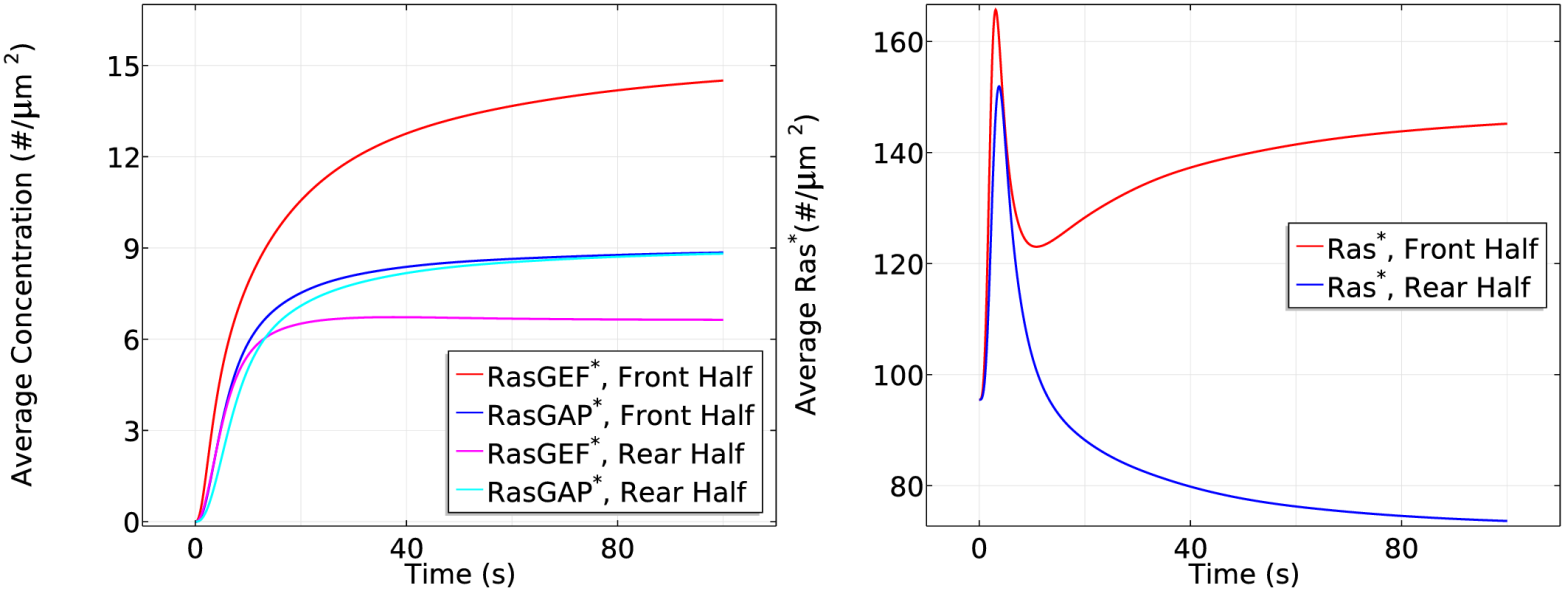
The time course of average RasGEF* and RasGAP* activity (left) and Ras* activity (right) at the front and rear halves in the absence of apparent RasGAP diffusion in the same gradient as previously used.

- Slow G_*βγ*_ diffusion
  In the absence of apparent G_*β*γ_ diffusion after dissociation, localized G_*β*γ_ leads to highly polarized activation of RasGEF (Fig 10 (left)). Correspondingly, in the transient activation phase the peak value of Ras* at the rear half is the same as in WT cells, but the peak at the front half increases from ∼165 #/*μm*^2^ to ∼172 #*μm*^2^ (cf. the right panel of Fig 10 and the left panel of Fig 7). Moreover, RasGAP activity is polarized (cf. the left panels of Figs 10 and 8), causing a stronger Ras* deactivation at the front. Hence we observe a slightly reduced steady state response (~140 #/*μm*^2^ v.s. ~143 #/*μm*^2^) in the front half during the symmetry breaking phase of Ras activation. It is not surprising that the reduced G_*β*γ_ diffusion still captures the biphasic behavior in the sense that $G_{\alpha_{2}}^{*}$ is still polarized and its downstream pathways are minimally affected. Although RasGAP* varies along the cell perimeter, it is counterbalanced by a stronger polarized RasGEF* (Note that both RasGEF* and RasGAP* at the front in the left panel of Fig 10 are much larger than the ones in the right panel of Fig 8).
- Slow RasGEF diffusion: the necessity of ‘activator’ diffusion
  The supply of RasGEF is localized on the membrane when RasGEF diffuses slowly, since $G_{\alpha_{2}}^{*}$ can only attract very limited RasGEF from the cytosol very close to the membrane. Moreover, diffusion of G_*β*γ_ ensures an almost uniform RasGAP and RasGEF activity at the front and the rear at steady state. Consequently, we observe that both the front and rear half of the cell adapts to the cAMP gradient and there is no Ras reactivation at the front due to limited availability of RasGEF, as shown in Fig 11. The front settles down at a slightly higher level of Ras* comparing to the rear due to a slightly stronger RasGEFactivity.
- Slow RasGAP diffusion: ‘inhibitor’ diffusion is not necessary
  Although the supply of RasGAP is also primarily restricted to the membrane when RasGAP diffuses slowly, there is enough RasGAP on the membrane due to the relatively small mean cAMP concentration (5.5 nM) in the gradient used. As a result, the biphasic behavior is not affected, as shown in Fig 12.
  In LEGI based models, the global diffusion of inhibitor is essential for inducing symmetry breaking. It is proposed [28] that the inhibitor might be RasGAP, but our model predicts that the diffusion of RasGAP is not a key component as long as there is sufficient amount of RasGAP on the membrane. Instead, a diffusible activator RasGEF becomes essential to induce symmetry breaking. In a LEGI scheme, the diffusion of inhibitor creates a uniform inhibitor distribution and the gradient induced nonuniform activator activity generates the symmetry breaking. In our model, the incoherent activation of both activator (RasGEF) and inhibitor (RasGAP) are induced through diffusing G_*β*γ_. Hence the Ras activity induced by G_*β*γ_ alone is balanced along the cell. In other words, the cell can not develop a sensitive gradient sensing from a diffusing G_*β*γ_. Alternatively, $G_{\alpha_{2}}^{*}$ facilitated pathways are the critical elements.
  We also tested scenarios in which both ${\text{G}}_{\alpha_{2}\beta \gamma }$ and Ric8 diffuse slowly, and when both G_*βγ*_ and RasGEF diffuse slowly (see Fig. E–Fig. G in S1 Text). In summary, various Ras activity patterns can be realized by controlling only the diffusion rates, thus revealing a potential role for diffusion in explaining the observed diverse sensitivities of genetically identical Dicty species in response to cAMP [61].

#### The dependence of Ras activation on the magnitude of the gradient and the mean concentration

**Fig 13:**
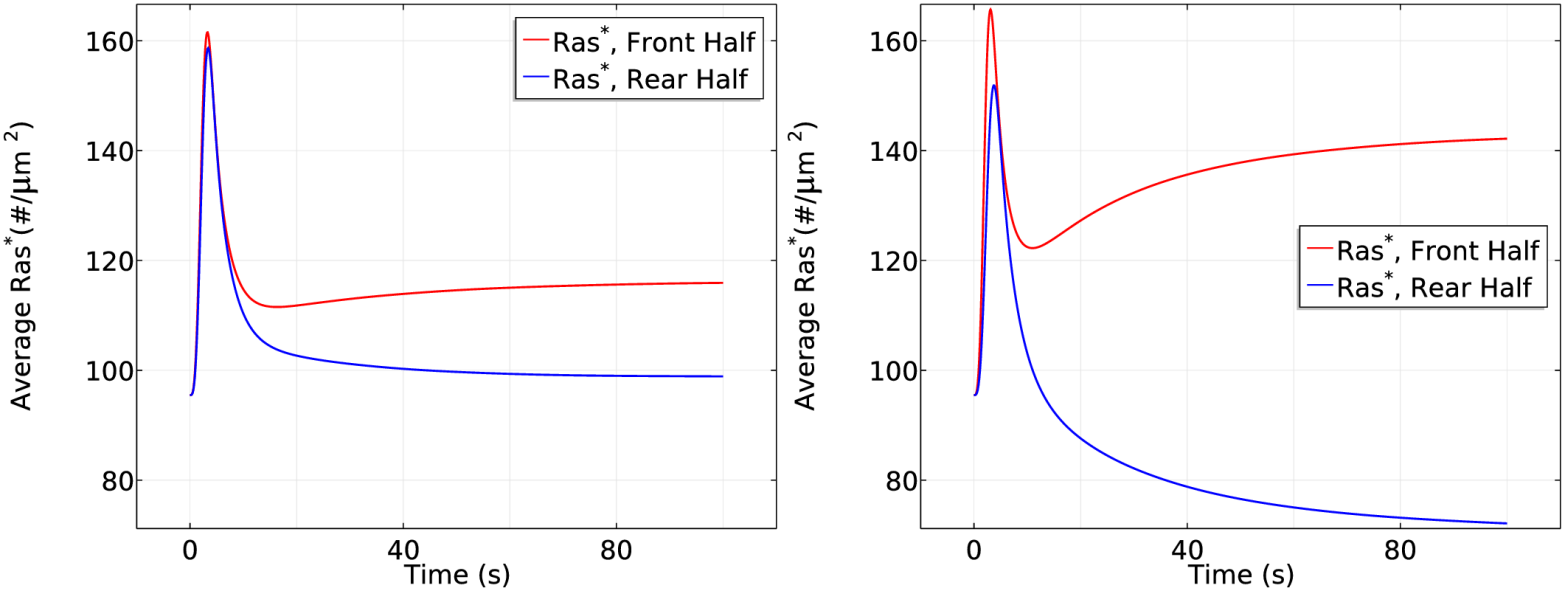
The dependence of Ras activation on the magnitude of the gradient. *Left*: The time course of average Ras* at the front and rear halves using *c*_*f*_ = 6.5 nM and *c*_*r*_ = 4.5 nM. *Right*: The time course using *c*_*f*_ = 10 nM and *c*_*r*_ = 1 nM.

To determine how the front-to-back gradient affects the activation of Ras, we stimulate the cell using two gradients: a shallow one with *c*_*f*_ = 6.5 nM and *c*_*r*_ = 4.5 nM, and the previously-used gradient with *c*_*f*_ = 10 nM and *c*_*r*_ = 1nM, both at the same mean cAMP concentration of 5.5 nM. The cell responses are shown in Fig 13. Ras activation is qualitatively similar in both a shallow gradient and a steep gradient, but smaller in magnitude in both phases for a shallow gradient. This is not surprising, since a steep gradient produces more G_*βγ*_ locally, which accounts for the slightly higher initial response, and a steeper $G_{\alpha }^{*}$ gradient that initiates the second phase. Note that the front-rear difference in a steep gradient is around 70 #/*μm*^2^ while the front-rear difference in shallow gradient is around 17#/*μm*^2^, giving a ratio of ∼4, which is roughly the ratio of the front-rear difference between the steep gradient (9 nM across the cell) and the shallow gradient (2 nM across the cell). Our model predicts results similar to those reported in [19], where gradient-dependent activation of Ras is observed.

**Fig 14:**
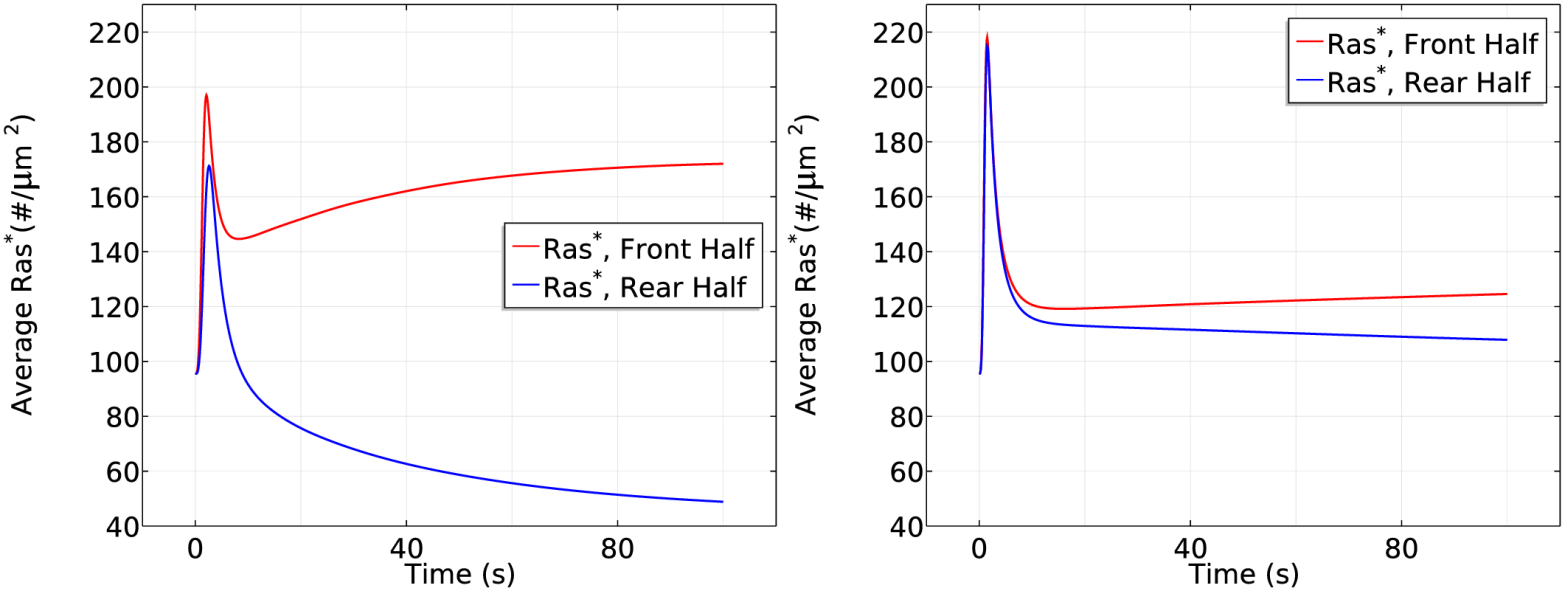
The dependence of Ras activation on the mean concentration. *Left*: The time course of Ras* at the front and rear halves using *c*_*f*_ = 50 nM and *c*_*r*_ = 0 nM. *Right*: The time course using *c*_*f*_ = 175 nM and *c*_*r*_ = 125 nM.

Next we test whether the cell responds differently in the same large gradient (5*nM*/*μm*) with different mean concentrations. As shown in Fig 14, in a steep gradient at a mean concentration of 25 nM, the front and back halves respond differently in the first phase of Ras activation—the front half reaches a maximum of 200#/*μm*^2^ while the rear half only reaches a maximum of 170#/*μm*^2^. Ras is reactivated at the front when the average Ras* drops to 150#/*μm*^2^ and symmetry breaking is well established after 100 seconds of cAMP stimulation, resulting in a 3.5 fold difference (120#/*μm*^2^) between the front half and rear halves. Surprisingly, we observe different response when the cell is exposed to the steep gradient at a higher mean concentration of 150 nM. In the first phase of Ras activation, the front and the rear responses almost exactly the same—both increase to a maximum of ∼220#/*μm*^2^—which is followed by a decrease to ∼120#/*μm*^2^. Then Ras is slowly reactivates at the front and the front-rear difference reaches less than 20#/*μm*^2^ after 100 seconds of stimulation.

**Fig 15:**
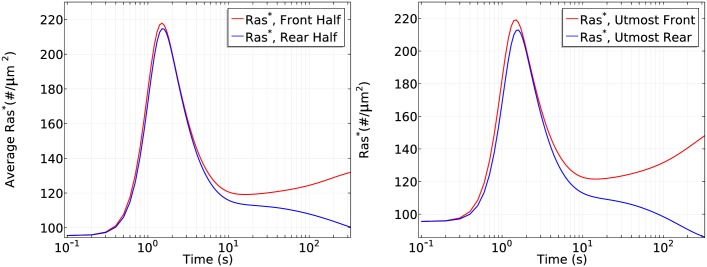
It takes longer to develop significant symmetry breaking for a higher mean concentration. *Left*: The log scale time course of average Ras* at the front and rear halves using *c*_*f*_ = 175 nM and *c*_*r*_ = 125 nM. *Right*: The Ras* activity in the same gradient at *x*_*f*_ and *x*_*r*_.

It is tempting to say that symmetry breaking is strongly reduced when the mean concentration increases to a saturation level, but strong symmetry breaking appears and the steady state difference between front and rear halves reaches approximate 1.3 fold if we observe the cell for a longer time, as shown in Fig 15. This shows that a higher mean concentration induces a more ‘uniform’ initial transient activation followed by much slower symmetry breaking.

**Fig 16:**
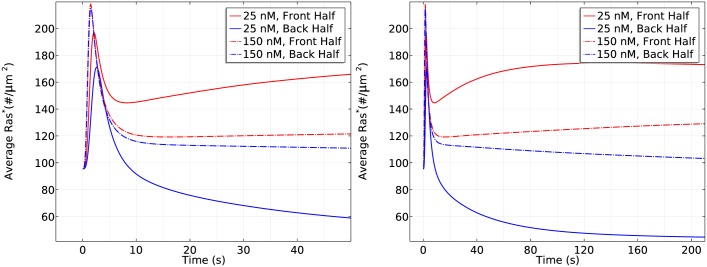
Mean concentration dependence of Ras activation. The solid lines correspond to a 5 *nM* = *μm* gradient with mean concentration 25 nM and the dash-dot lines correspond to a 5 *nM* = *μm* gradient with mean concentration 150 nM. *Left*: 0–50s. *Right*: 0–200s.

To demonstrate the mean concentration dependence of Ras activation more clearly, we plot the Ras activation patterns in the two gradients for the first 50 seconds and the first 200 seconds separately in Fig 16.

From the left panel of Fig 16, one sees that the reactivation of Ras starts at around 6 seconds at a mean concentration 25*nM* and the front-back difference is well established at *t* = 50s. In contrast, Ras is barely reactivated at the front when the mean concentration is 150 *nM* in the first 50 seconds, but Ras is gradually reactivated at the front and the front-back difference becomes significant at *t* = 200s.

#### No symmetry breaking in *g*_*α*2_-null cells

**Fig 17:**
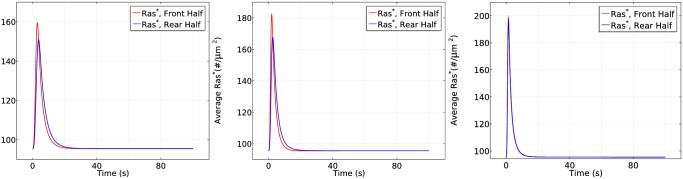
The time course of average Ras* activity in *g*_α2_-null cells under various gradients. *Left*: The time course of Ras* at the front and rear halves using *c*_*f*_ = 10 nM and *c*_*r*_ = 1 nM in *g*α2-null cells. *Center*: The Ras* activity using *c*_*f*_ = 50 nM and *c*_*r*_ = 0 nM in *g*α2-null cells. *Right*: The Ras* activity using *c*_*f*_ = 175 nM and *c*_*r*_ = 125 nM in *g*α2-null cells.

It is reported [19] that in *g*_*α*2_-null cells, the cAMP gradient induces a short transient uniform Ras activation but the specific upgradient Ras reactivation never occurs. We test our model for *g*_*α*2_-null cells by blocking the $G_{\alpha_{2}}^{*}$ promoted RasGEF and Ric8 localization, and the simulation results are illustrated in Fig 17 for different gradients and same gradient with different mean concentrations. In all three gradients we tested, *gα*2-null cells only exhibit the initial transient activation of Ras in consistent with the experimental findings. The cell settles down at the same level of Ras* at both the front and rear of the cell, suggesting the failure of direction sensing. Both the experimental measurements and computational simulation reveal the essential role of $G_{\alpha_{2}}^{*}$ in generation of direction sensing.

#### No direction sensing when ric8-null cells are exposed to a shallow gradient or a steep gradient with high mean concentration

**Fig 18:**
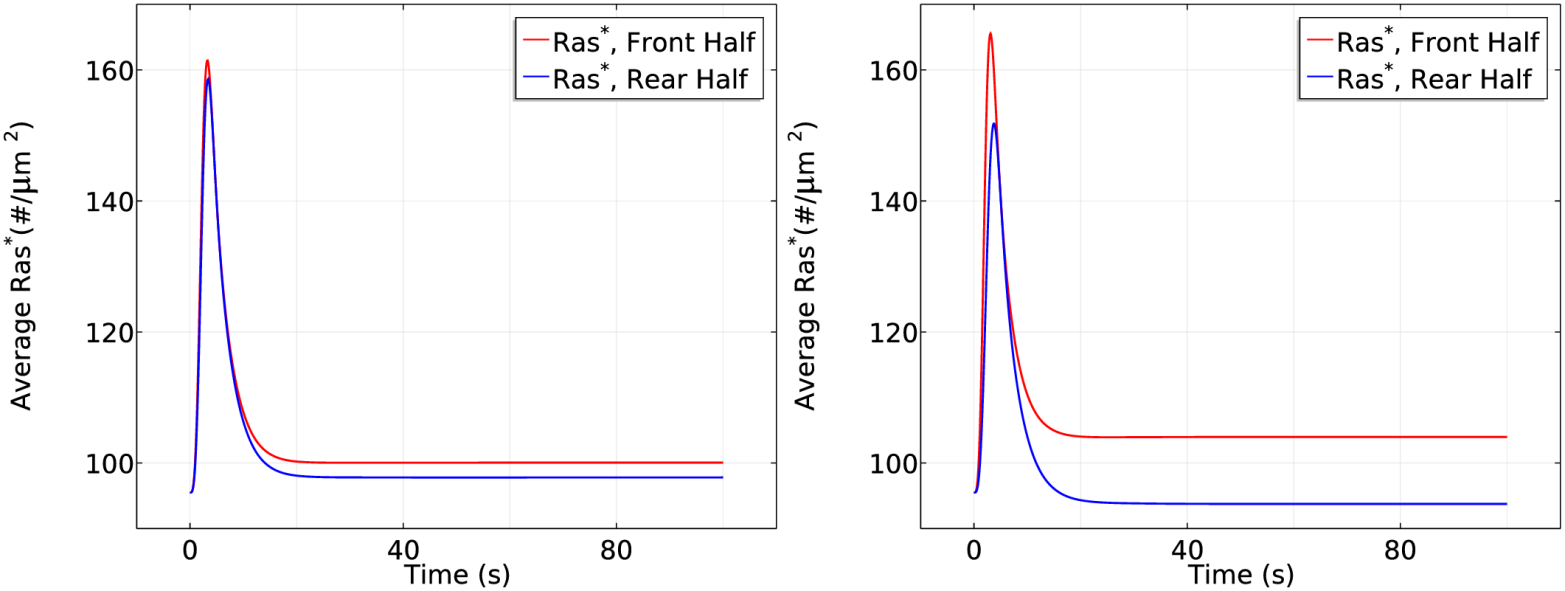
The time course of average Ras* activity in ric8-null cells under non-steep gradients. The time course of average Ras* at the front and rear halves in ric8-null cells. *Left*: The gradient set by using *c*_*f*_ = 6.5 nM and *c*_*r*_ = 4.5 nM. *Right*: The gradient set by using *c*_*f*_ = 10 nM and *c*_*r*_ = 1 nM.

Recall that ric8-null cells have a decreased ${\text{G}}_{\alpha_{2}\beta \gamma }$ dissociation at the steady state compared with WT cells in uniform stimulus, and here we test whether ric8-null cells are able to sense directions effectively in a cAMP gradient. Ras activation is illustrated in Fig 18 when ric8-nulls are exposed to gradients of the same mean concentrations with different steepness. Comparing with the plot in the left panel of Fig 13, the average front-rear difference is reduced 8 fold for the shallow gradient (from ∼ 15#/*μm*^2^ in WT cells to ∼2#/*μm*^2^). Consistent with experimental findings [22], the almost identical Ras* activity at the front and rear suggests failure of direction sensing when ric8-null cells are exposed to a shallow gradient. The plot in the right panel suggests that the cell is still able to sense direction when the gradient is large enough, but the biphasic responses disappear.

**Fig 19:**
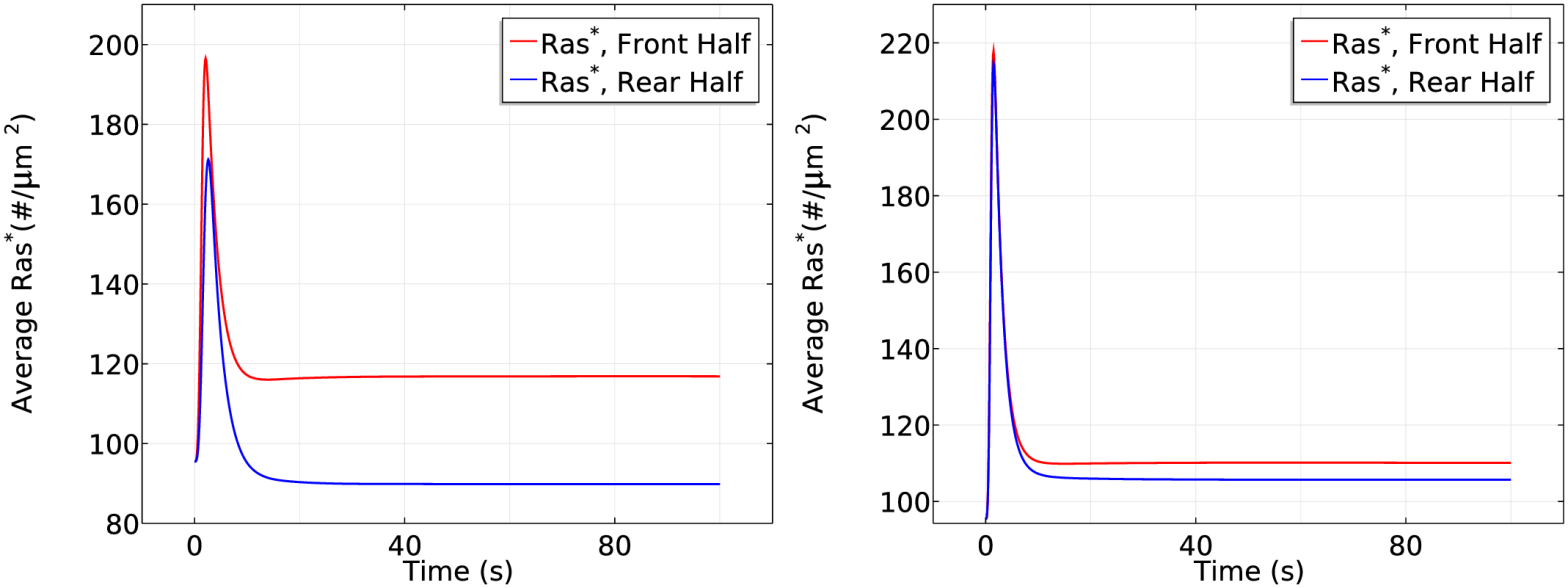
The time course of average Ras* activity in ric8-null cells under steep gradients. The time course of average Ras* at the front and rear halves in ric8-null cells in steep gradients. *Left*: The gradient set by using *c*_*f*_ = 50 nM and *c*_*r*_ = 0 nM. *Right*: The gradient set by using *c*_*f*_ = 175 nM and *c*_*r*_ = 125 nM.

It has been shown that ric8-null cells migrate with an efficiency similar to that of wild-type cells when cells are exposed to a steep gradient of cAMP (>10*nM*/*μm*) [22]. We tested our model with a gradient of 5*nM*/*μm* with different mean concentrations, and the results are shown in Fig 19. As shown in the left figure, ric8-null cells still sense direction by creating an asymmetrical distribution of Ras*. However, the asymmetry is strongly reduced comparing to WT cells (left panel of Fig 14). Moreover, ric8-null cells do not exhibit a biphasic response. Instead, the front and rear half of the cell settle at different levels after initial transient activation. Surprisingly, when the mean concentration is elevated to 150 nM, ric8-null cells lose the ability to sense direction, as shown in the right panel of Fig 19 (front rear difference is less that 5#/*μm*^2^). Hence our model predicts that Ric8 is essential for chemotaxis in both shallow gradients of cAMP and steep gradients with high mean concentration. In the range of cAMP gradients where ric8-null cells can sense direction, our model predicts that there is no biphasic Ras activation and little amplification.

### A solution to the back-of-the-wave problem

**Fig 20:**
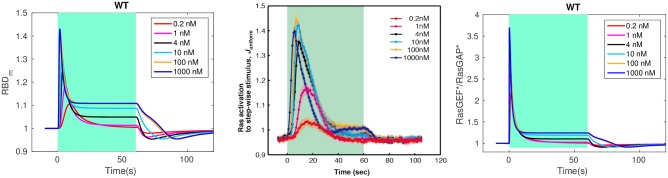
Rectification in WT cells. *Left*: The time course of membrane RBD under uniform stimuli of various concentrations. *Center*: experimental measurements extracted from [21]. *Right*: The time course of the ratio of RasGEF* to RasGAP*.

In the context of Dicty aggregation, the ‘back-of-the-wave’ problem refers to the fact that cells do not turn to follow the cAMP gradient after the wave has passed, despite the fact that the spatial gradient reverses as the wave passes over a cell [15, 62]. This requires some level of persistence of ‘orientation’ of a cell, but there is as yet no agreed-upon mechanistic solution for this problem, since polarization and other factors may play a role [63]. Under uniform stimuli, cells are said to show rectification if there is an asymmetry in the amplitude and evolution of the response to a step increase in cAMP compared with the response following removal of the stimulus [21]. To test whether the proposed network exhibits rectification in this sense, we apply a uniform stimulus of various concentrations for 60 seconds and then remove it, as was done experimentally in fully aggregation-competent cells [21]. Fig 20 (left and center) show the simulation and the experimental results, resp. In both cases the concentration of cAMP is increased from 0 M to the concentrations indicated for 60 seconds (green shaded area), followed by a decrease to 0 M, and in both cases one sees a much larger and faster change in RBD following application of the stimulus than on removal. We also applied the same stimuli as used above to *g*_*α*_-null cells and ric8-null cells. Results given in the Supporting Information show that Ric8 plays a significant role in the rectification, as will also be seen later in the traveling wave analysis.

Some insight into this behavior can be gained from simple models of excitation and adaptation, such as the cartoon description defined by the system of equations

$$\frac{dy_{1}}{dt}=\frac{S\left(t\right)-\left(y_{1}+y_{2}\right)}{t_{e}},\;\;\;\;\frac{dy_{2}}{dt}=\frac{S\left(t\right)-y_{2}}{t_{a}}.$$ (1)

Here *S*(*t*) represents the signal and the magnitudes of *t*_*e*_ and *t*_*a*_ reflect the time scale for excitation and adaptation, resp., and one see that *y*_1_ adapts perfectly to a constant stimulus whereas *y*_2_ compensates for the stimulus. However, the temporal responses to increasing and decreasing stimuli are symmetric, and therefore such a simple model cannot explain the observed response. Nakajima et al. [21] suggest that a single-layered incoherent feedforward circuit with zero-order ultrasensitivity [64] is necessary to generate rectification, but our model does not include an ultrasensitive circuit. Instead, rectification is induced solely by the balanced regulation of RasGEF and RasGAP activity. The ratio of RasGEF* to RasGAP* increases 2–4 fold very rapidly in response to a step increase in the cAMP concentration, but when the stimulus is removed this ratio does not drop significantly, as shown in the right panel of Fig 20. Thus Ras activation persists because the ratio equilibrates rapidly while the absolute levels of the factors decrease more slowly.

**Fig 21:**
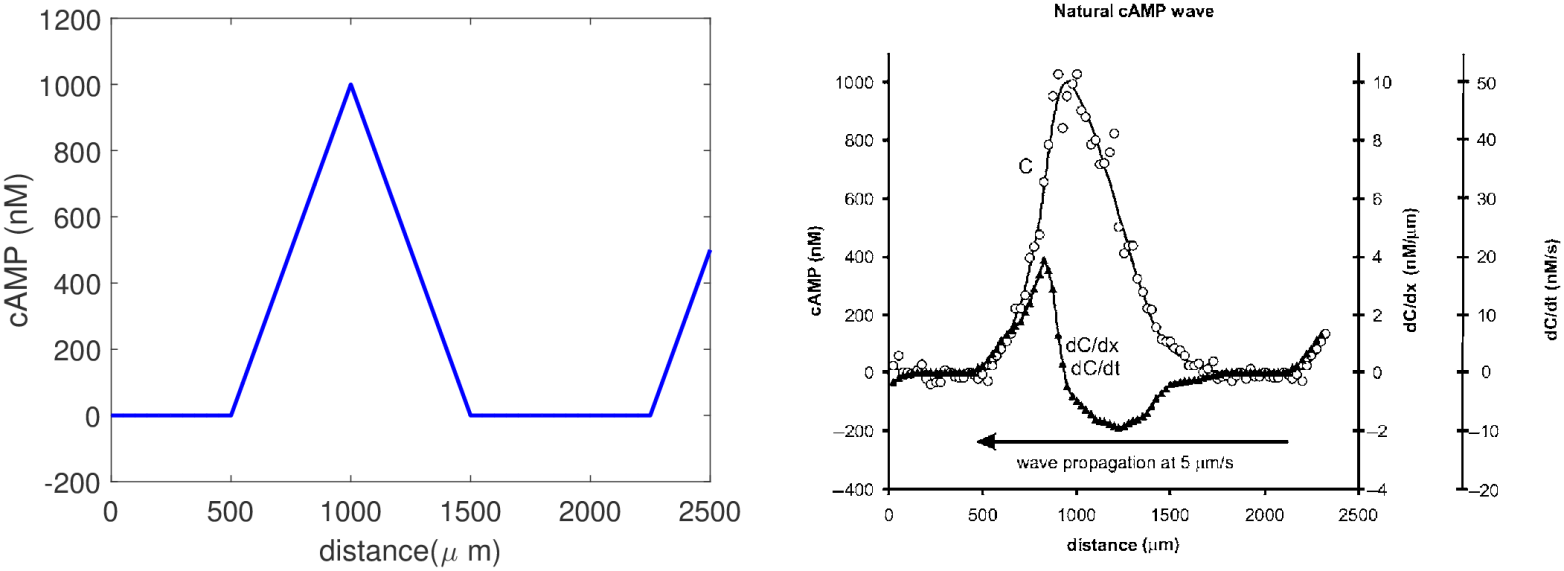
The simulated cAMP wave and a natural cAMP wave. *Left*: The triangle wave. *Right*: A natural wave—from [65] with permission.

To study how cells would respond in wave-like spatially-graded stimuli, we first generate a simple trianglular wave that approximates a natural cAMP wave. Let *W*(*x*, *y*, *z*, *t*) denote the cAMP concentration at (*x*, *y*, *z*) of the cell at time *t*, and specify it as

$$W\left(x,y,z,t\right)=\left\{\begin{array}{cc} 0, & 0+350k\leq t\leq \frac{x+5}{v}+350k\\ 10(t-\frac{x+5}{v}-350k), & \frac{x+5}{v}+350k<t\leq \frac{x+5}{v}+100+350k\\ -10(t-\frac{x+5}{v}-350k)+2000, & \frac{x+5}{v}+100+350k<t\leq \frac{x+5}{v}+200+350k\\ 0, & \frac{x+5}{v}+200+350k<t\leq 350\left(1+k\right) \end{array}\right.,$$

where *v* is the wave speed and −5 ≤ *x*, *y*, *z* ≤ 5, *k* = 0, 1, ⋯. This wave resembles a natural wave when we choose the natural wave speed *v* = 5*μm*/*s*, as shown in Fig 21. The wave length is 1000*μ*m, and at the natural speed any point on a cell is subject to an increasing stimulus for 100 sec on the upstroke of the wave and a decreasing stimulus for 100 sec on the downstroke.

**Fig 22:**
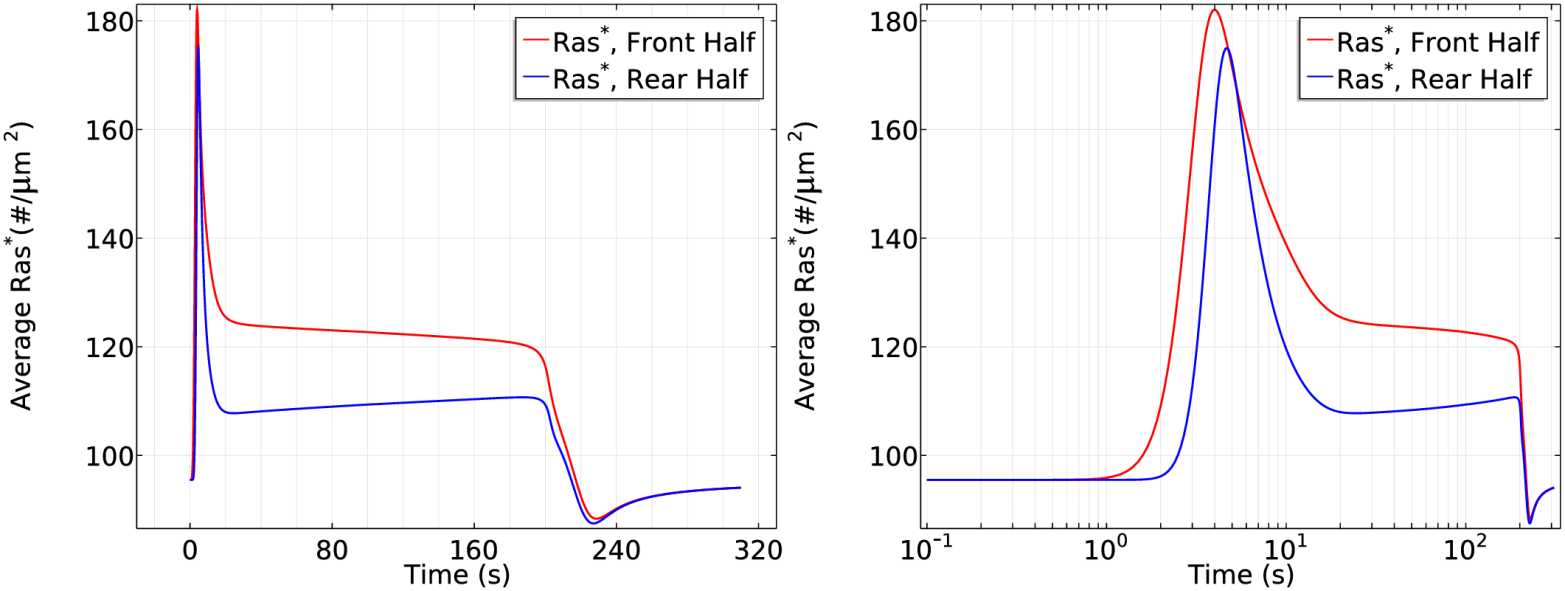
The time course of average Ras* activity in a triangle wave at normal wave speed *v* = 5*μm*/*s*.

**Fig 23:**
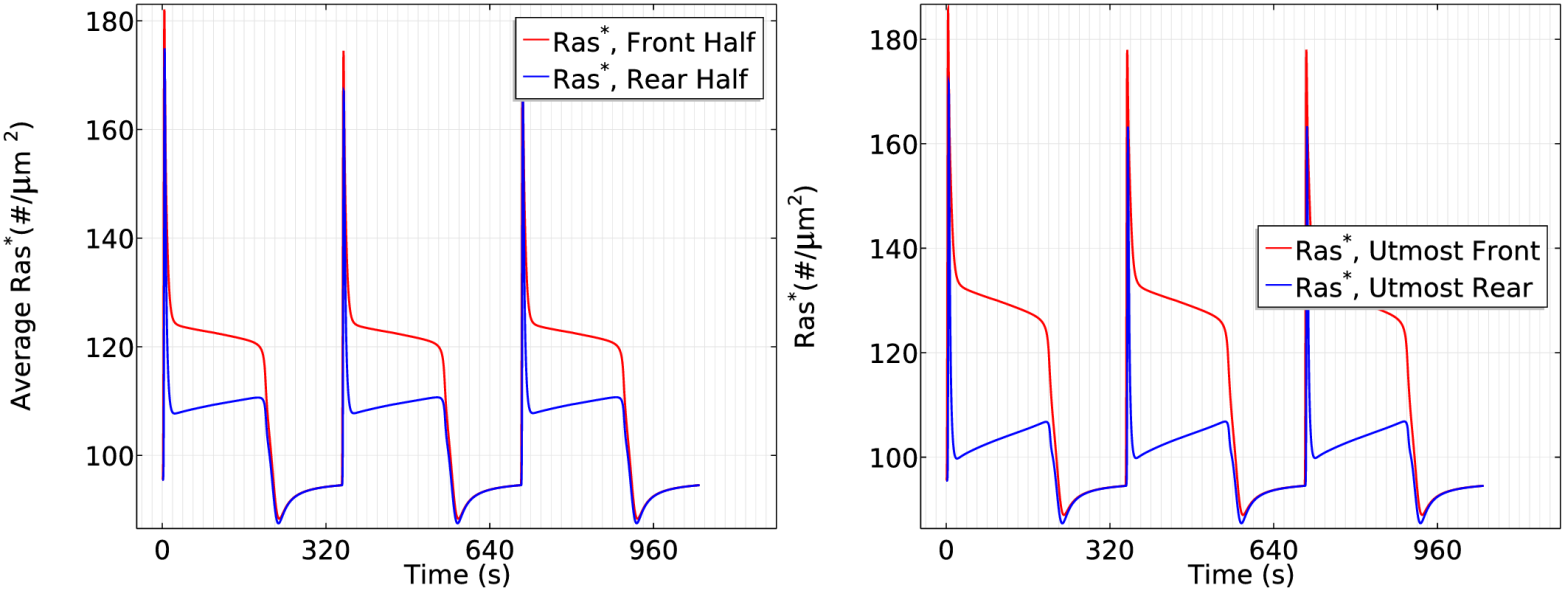
Cell responses to consecutive waves. *Left*: The time course of the front and rear halves when three waves pass the WT cell at *v* = 5*μm*/*s*. *Right*: The time course of Ras* activity at the antipodal points.

As shown in Fig 22, Ras is activated everywhere as the wave passes, but Ras activation is delayed about 1 sec in the rear half (Fig 22 -right) for a wave traveling at the natural wave speed. Ras activation is higher at the front of the cell than at the rear throughout passage of the wave, thereby providing persistent directionality in Ras activation and the potential for persistent orientation as the wave passes. It should be emphasized that we are simulating the rounded LatA-treated cells that have no intrinsic polarity, which suggests that polarity is not necessary for the persistence of direction sensing at the natural wave speed, even at the level of Ras activity. By comparing Figs 20 and 22, one sees a similar pattern in Ras activation. In fact, due to the rectification characteristic observed in uniform stimuli, Ras* activity does not drop significantly in a wave, and therefore the front is able to maintain a higher Ras*. To determine whether the cell is able to respond after the first wave passes, we applied the same wave for three periods, and one sees in Fig 23 that the cell responses are almost identical for three successive passages of a wave.

**Fig 24:**
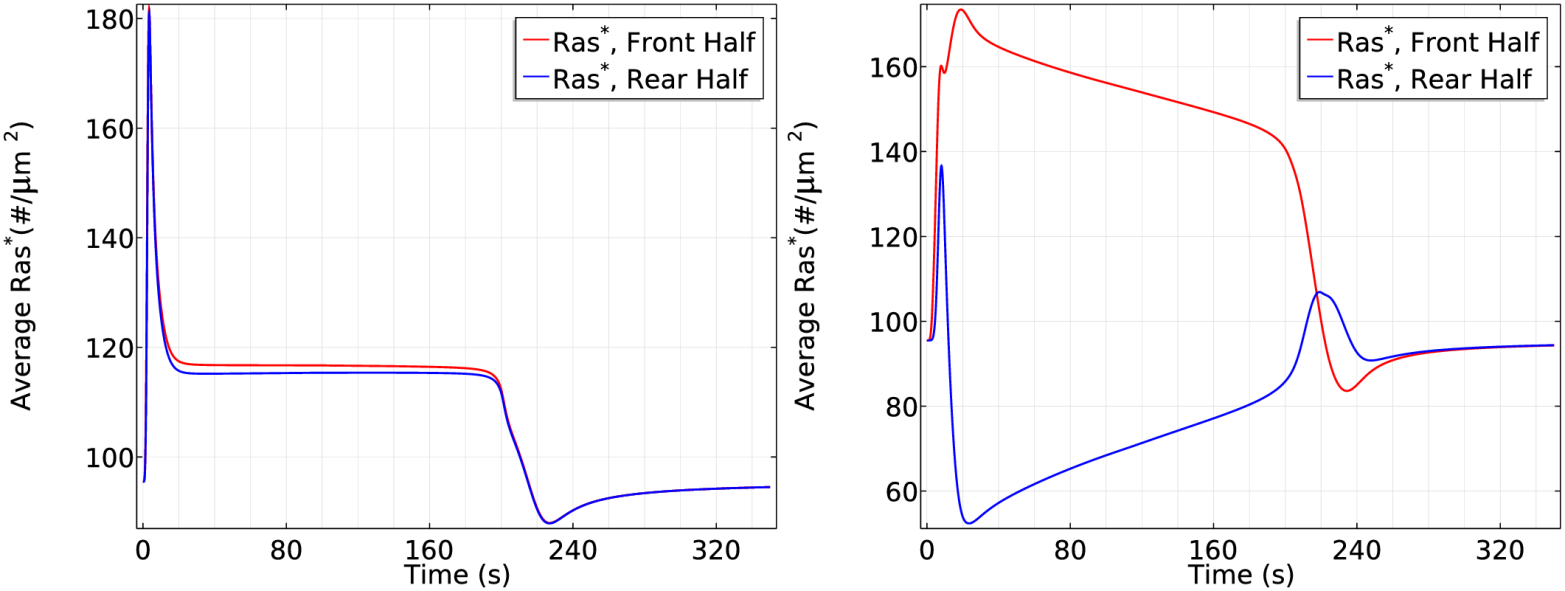
The time course of Ras activation at the front and rear halves for a wave speed *v* = 50*μm*/*s* (left), and *v* = 0.5*μm*/*s* (right).

It is also known that wave speeds affect the spatial pattern of Ras activity over a cell [21], in that Ras is activated uniformly for a fast wave, and activated at both the wavefront and waveback for slow waves. To test the effects of the wave speed, we apply a fast wave (50*μm*/*s*) and a slow wave (0.5*μm*/*s*) to the rounded LatA-treated cells. The results are shown in Fig 24. At a wave speed of 50*μm*/*s*, Ras activation is uniform along the cell periphery, as is observed in the experiments, but at 0.5*μm*/*s* we see a significant Ras reactivation at the rear of the cell and the Ras* distribution reverses at the back of the wave.

**Fig 25:**
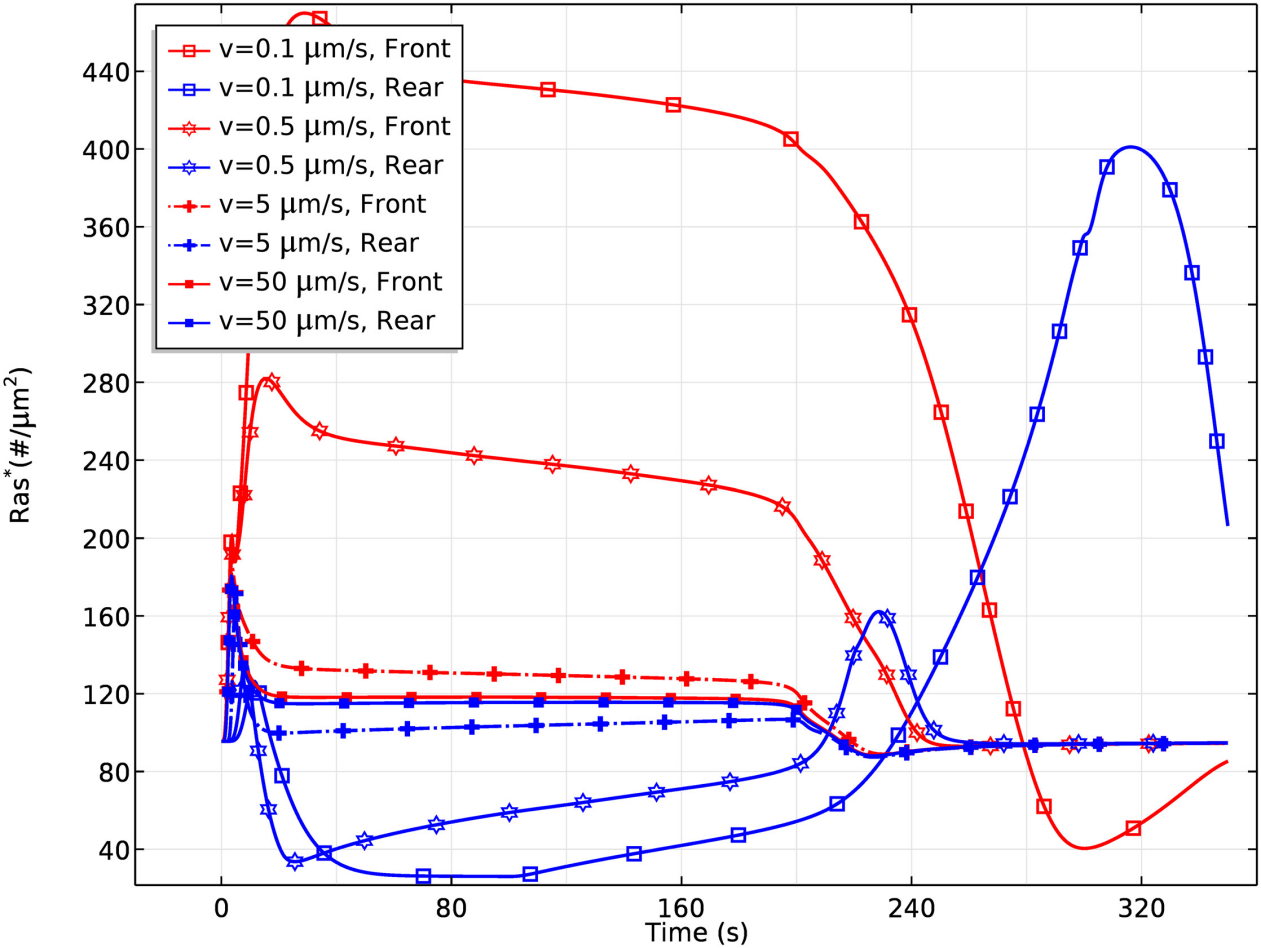
The time course of Ras activation at the extreme front and rear points for different wave speeds.

In order to demonstrate the effect of wave speed on rectification more clearly, we plot the time course of Ras activation at the front-most and rear-most points of the cell in Fig 25. At a wave speed of 0.1*μm*/*s*, Ras is reactivated at the rear of the cell when the back of the wave passes over the rear. As the wave speed increases, the reactivation at the rear becomes weaker, and at the normal wave speed of 5*μm*/*s* persistent directionality is well-preserved. Of course, when a fast wave passes over the cell, Ras activation is almost spatially uniform.

**Fig 26:**
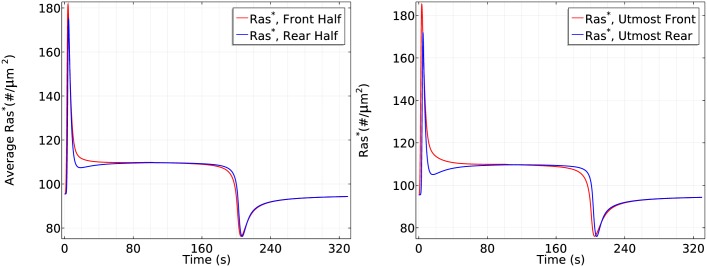
Cell responses to the triangle wave in ric8-null cells. *Left*: Time course of the front and rear half when the triangle wave passes the ric8-null cell at *v* = 5*μm*/*s*. *Right*: Time course of the point Ras* activity.

As was pointed out earlier, Ric8 plays an essential role in rectification under uniform stimuli, and to further emphasize that the back of the wave problem is closely connected with the disparity in the response to increasing *vs.* decreasing stimuli, we applied the same wave used previously to a ric8-null cell. The Ras* activity is shown in Fig 26, where one sees that the persistence of directional information is essentially lost. It is not surprising to see that Ras* at the front becomes smaller than the rear, which indicates a reversal in the Ras* distribution, further reinforcing the importance of the asymmetric response to increasing *vs* decreasing stimuli in solving the back of the wave problem.

### The trade-off between persistence of directionality and the ability to reorient

**Fig 27:**
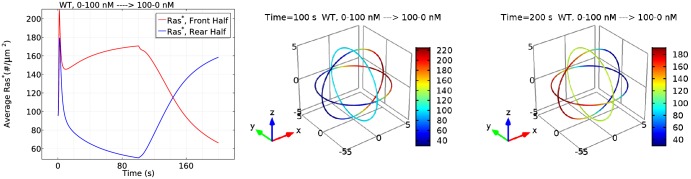
The response to gradient reversal. A linear gradient of 10*nM* = *μm* with mid point 50 nM (0–100 nM) is applied at t = 0 s and reversed at t = 100 s. The time course of average Ras* at the front and rear halves of WT cells (left) and the spatial profile of Ras* on three great circles on the sphere at t = 100 s (center) and at t = 200 s (right).

**Fig 28:**
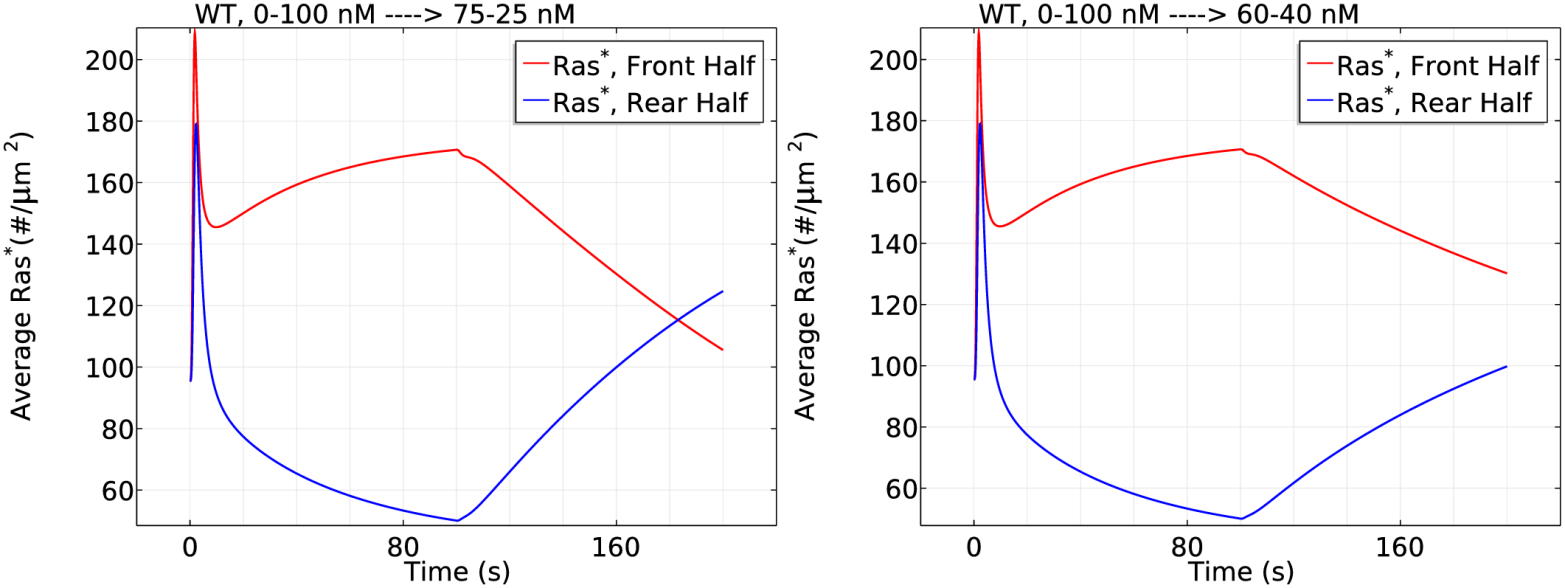
The response when the reversed gradient is shallower. *Left*: 75–25 nM after 100 seconds. *Right*: 60–40 nM after 100 seconds.

Clearly there is a trade-off between the persistence of directionality in Ras activation and the ability of cells to respond to new gradients. To investigate whether the Ric8-induced rectification has an adverse effect on reorientation in response to a reversed gradient, we subject cells in a 0–100 nM gradient to reversals to increasingly weaker gradients. In each case we keep the mean concentration experienced by the cell fixed to eliminate the mean concentration effect (see. Fig 14). For an equally strong reverse gradient (100–0 nM), the directional persistence of Ras* is reversed within 100 seconds of gradient reversal, as shown in Fig 27. The spatial profile also indicates that Ras* distribution is strongly reversed after switching to equally strong reversed gradients, (Fig 27 –center and right). It is observed in Dicty that all cells (20/20) reversed their direction of migration under this protocol [22]. For intermediate gradients (75–25 nM), Ras* is slightly reversed (Fig 28 –left) in the same time window (0–200 s). The spatial plot of Ras* indicates spatial oscillations along the cell periphery at almost the end of the time window *t* = 180 s, (see Fig. M in S1 Text) suggesting spatio-temporal complexity in Ras* redistribution. Consistently, experiments show that a fraction of the cells (5/17) did not reverse their migration direction. For weak gradients (60–40 nM) a difference in Ras activation is still maintained at the end of the time window (*t* = 200 s) (Fig 28 (right)), consistent with the observation that that all cells continued moving in their original direction in this case [22]. These simulations suggest that Ric8-induced rectification does not harm cells’ reorientation in response to large amplitude reversals of the gradient, but it delays the reorientation in a weak reversed gradient.

### Variants of the model

#### Robustness of the G_*α*_-G_*βγ*_-Ric8 triangle

In the current signal transduction mechanism Ric8 cycles between a cytosolic pool and the membrane, where it is activated by G_*βγ*_ and it in turn reactivates G_*α*_. There is some evidence in other systems that Ric8 may not require an activation step on the membrane [66, 67], and here we investigate the robustness of the G_*α*_-G_*βγ*_-Ric8 triangle by considering other possibilities. For convenience in comparing schemes, we call the current translocation-activationmechanism *Mode 1*, and consider two alternative schemes.

- *Mode 2*: Translocation-only mechanism. Reaction ⑧ and ⑩ in Table 1 are eliminated. ⑨ is modified so that *Ric*8_*m*_ reactivates G_*α*_ directly.
- *Mode 3*: Alternative translocation-only mechanism. We remove the activation steps as in Mode 2, and G_*α*_ is assumed to be the membrane recruitment promoter in reaction ⑦.

The simulations demonstrate that *Mode 2* still captures the basic characteristics of Ras activation, very similar to the results for *Mode 1*, except that the magnitudes are slightly changed (see Fig. I and Fig. J in S1 Text). This suggests that G_*βγ*_ activation (Reaction ⑧ in Table 1) is not an essential step.

As for *Mode 3*, it is shown that the cell is still able to sense direction and exhibit biphasic responses under various cAMP gradients (see Fig. K and Fig. L in S1 Text). They differ from the results in *Mode 1* and *Mode 2* in that the point Ras activity equilibrates more rapidly and the magnitudes of the front-back differences are smaller.

These results demonstrate the robustness of the the G_*α*_-G_*βγ*_-Ric8 triangle in the signal transduction pathways, providing flexibility in modeling this triangle.

#### Amplification at the level of Ras

It has been reported that the gradient of active Ras across the cell is substantial in an imposed cAMP gradient [68]. Recent quantitative analysis also suggests that amplification may occur at the level of Ras [19]. We test the magnitude of amplification by calculating the amplification factor [69, 70]

$$\sigma =\frac{\left(Ras_{f}^{*}-Ras_{r}^{*}\right)/Ras_{m}^{*}}{\left(cAMP_{f}-cAMP_{r}\right)/cAMP_{m}},$$

where *X*_*m*_ is the mean value of *X*. *X*_*f*_ and *X*_*r*_ are the concentrations of *C* at the point on the cell surface exposed to the highest and lowest concentration of stimulus, respectively. If *σ* > 1, Ras* the signal is amplified.

The amplification factors are summarized in Table 2. As one sees in the table, the signal is amplified at the level of Ras in both Mode 1 and Mode 2, but the signal amplification indices for Mode 3 are smaller than 1, which indicates that the signal is not amplified.

**Table 2:** Amplification factors under various modes and gradients.

	1–10 nM	0–50 nM	125–175 nM
Mode 1	1.3	1.7	2.7
Mode 2	1.6	2.0	1.6
Mode 3	0.6	0.7	0.7

There are two sources of amplification in the proposed network. Firstly, the higher concentration of $G_{\alpha_{2}}^{*}$ on the membrane at the front of the cell induces a higher localization and activation of Ric8, which reactivates $G_{\alpha_{2}}$ and further promotes RasGEF localization at the front. Secondly, faster ${\text{G}}_{\alpha_{2}\beta \gamma }$ reassociation at the back due to higher $G_{\alpha_{2}}^{*}$ hydrolysis induces a faster ${\text{G}}_{\alpha_{2}\beta \gamma }$ cycling, providing more ${\text{G}}_{\alpha_{2}\beta \gamma }$ at the back. As a result, the faster reassociated *G*_*αβγ*_ at the back can provides a source of *G*_*αβγ*_ needed at the front by diffusion, which creates an imbalanced sequestration of *G*_*αβγ*_ between the front and the back. These two positive feedback loops are built into Mode 1 and Mode 2, but not into Mode 3.

In models based on LEGI, the local-excitation, global-inhibition mechanism provides no signal amplification—other mechanisms have to be added [71, 72]. In one speculative mechanism for amplification an ultrasensitive transfer function is incorporated [21, 73], but such mechanisms are very sensitive to parameter choices [73]. LEGI and ultrasensitivity are not bulit into our proposed network, and the positive feedback loops are responsible for the signal amplification at the level of Ras activation. Of course additional amplification can result at the level of PIP_3_ [61], which is activated by Ras, as will be shown in a model under investigation.

#### The effect of cell shape

Heretofore we have assumed that the cell is pretreated with LatA, hence the cell is spherical with radius *r* = 5*μm*. To investigate how cell shape may alter the Ras* dynamics, we construct an ellipsoid with the same volume as that of the standard cell. By assuming that the ellipsoid is prolate, we have

a=10μm,b=c=3.5μm.

**Fig 29:**
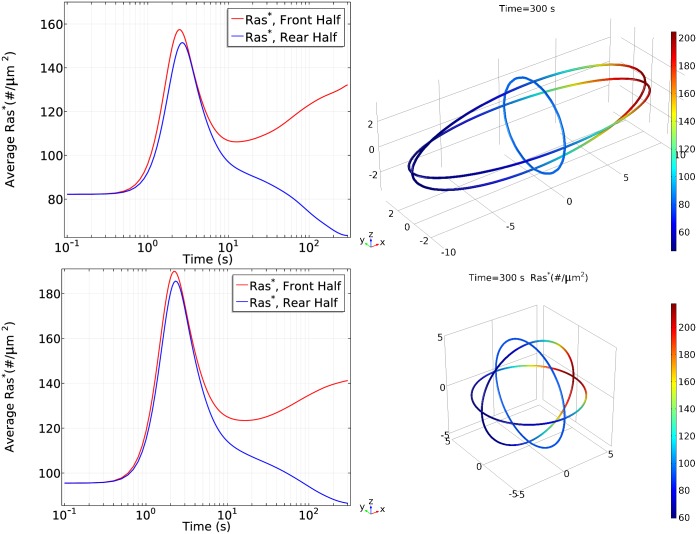
Effects of cell shapes. *Top*: Ras activity for a ellipsoidal cell. *Left*: Average Ras* at front and rear half of WT cells; *Right*: The spatial profile of Ras* at t = 300*s*; *Bottom*: Ras activity for a sphere cell. *Left*: Average Ras* at front and rear half of WT cells; *Right*: The spatial profile of Ras* at t = 300*s*.

To test the effect of this shape change, we applied a cAMP gradient of 1000*pM*/*μm* with a 25 nM midpoint, and the resulting responses are shown in Fig 29. The basic characteristics of Ras activation are still maintained for an ellipsoidal cell: the cell first experiences a transient activation both at the front and rear; then Ras is reactivation at the front and a clear symmetry breaking emerges.

Fig 29 illustrates how cell shapes affect Ras activity. On one hand, the density of molecules is reduced when the cell is changed from a sphere to an ellipsoid with the same volume. Hence we see that the peak of first phase for an ellipsoid is smaller than for a sphere due to lower availabilities of molecules, although the endpoint cAMP sensed by a cell is increased from a 10 nM difference (20–30 nM) to a 20 nM (15–35 nM) difference. On the other hand, although the point Ras* at the frontal point for a ellipsoid cell is higher than a sphere cell (see right panels of Fig 29), the average Ras* at the front half of the ellipsoid cell is still smaller than for the sphere cell, suggesting that the larger gradident does not compensate for the smaller molecular densities.

## Discussion

Chemotaxis is a dynamic spatio-temporal process that involves direction sensing, polarization, and cell movement, and direction sensing is the first essential step in this process, becuase it defines the cell’s compass. A growing body of evidence suggests that Ras is an ideal candidate within the chemotactic signalling cascade to play an essential role in direction sensing [31, 68]. In this article, we developed a novel modular model of direction sensing at the level of Ras activation. The model incorporates biochemical interactions in Dicty and captures many aspects of its response. The model consists of the cAMP receptor, the G-protein ${\text{G}}_{\alpha_{2}\beta \gamma }$, and a Ras GTPase module in which both adaptation and amplification occur. Utilizing a rounded cell pretreated by LatA as was done in experiments, we investigated Ras activation patterns in various cAMP stimuli. Simulations of this model give insights into how the signal transduction network determines Ras activation characteristics in wild type cells, how an altered network in mutant cells changes Ras activation, and how the spatial profile and persistence of Ras activation can lead to directional persistence.

We proposed an experimentally-based kinetic model of ${\text{G}}_{\alpha_{2}\beta \gamma }$ signaling in which the intact ${\text{G}}_{\alpha_{2}\beta \gamma }$ and the G_*β*γ_ subunit can cycle between the membrane and the cytosol, while the $G_{\alpha_{2}}$ subunit remains membrane-bound. Moreover,$G_{\alpha_{2}}$ can be reactivated by the only known (to date) GEF for $G_{\alpha_{2}}$, Ric8. The regulation of Ric8 is not well-defined, but we assume that it is also cycles between the cytosol and the membrane, and that its recruitment to the membrane is promoted by $G_{\alpha_{2}}^{*}$. The model replicates the persistent *G*_*αβγ*_ dissociation in the presence of cAMP, and also demonstrates that G_*β*γ_ and $G_{\alpha_{2}}^{*}$ are produced in a dose-dependent manner. Interestingly, the model reveals that $G_{\alpha_{2}}$ exhibits dose-dependent kinetic diversities. The variety of $G_{\alpha_{2}}$ dynamics revealed here may have important implications in direction sensing because in neutrophils $G_{\alpha_{2}}$-GDP accumulates at the leading edge and is involved in regulating directionality [74], although it has not been demonstrated that Ric8 is involved there.

Adaptation of Ras activity is controlled by a balance between RasGEF and RasGAP, both of which can cycle between the membrane and the cytosol. This component of the network involves incoherent feed-forward, and becuase both can cycle betweenmembrane and cytosol, can give rise to spatial asymmetry in Ras activation. Both RasGEF and RasGAP are activated at the membrane by free G_*β*γ_, but the translocation of RasGEF from the cytosol is enhanced by $G_{\alpha_{2}}^{*}$. The proposed translocation-activation topology is able to capture the dose-dependent Ras activation and various patterns such rectification and refractoriness under uniform stimuli. It also predicts that imperfect adaptation is inevitable in wild type cells due to the asymmetrical translocation of RasGEF. Takeda *et al.* [28] proposed an incoherent feedforward activation model to explain adaptation of Ras activity in which RasGEF is assumed to be confined to the membrane and RasGAP diffuses in the cytosol. In our model, both RasGEF and RasGAP can diffuse in the cytosol at equal rates, and both can be recruited to the membrane and activated by G_*β*γ_.

Direction sensing, biphasic Ras activation and signal amplification are achieved by complex interactions between the modules. The incoherent-feedforward-activation by globally-diffusing G_*β*γ_ contributes to a transient activation along the entire cell perimeter. The activation at the front of the cell (facing the higher cAMP concentration) is initially faster and stronger due to the cAMP gradient, but it provides no symmetry breaking or signal amplification since diffusion eliminates the initial G_*β*γ_ concentration gradient. This means that G_*βγ*_ does not reflect the external stimulus gradient and provides no basis for direction sensing in LatA-treated cells, although it is essential for RasGEF and RasGAP activation. It is the Ric8 regulated, membrane-bound $G_{\alpha_{2}}^{*}$ that determines the symmetry breaking and signal amplification. $G_{\alpha_{2}}^{*}$ creates an asymmetrical recruitment of RasGEF in a cAMP gradient, which in turn induces asymmetrical RasGEF activation, providing a basis for symmetry breaking. More importantly, Ric8 recruitment to the membrane is elevated by $G_{\alpha_{2}}^{*}$, while activated Ric8 reactivates $G_{\alpha_{2}}$, forming a positive feedback loop. In addition, faster ${\text{G}}_{\alpha_{2}\beta \gamma }$ reassociation at the back of the cell due to less reactivation of $G_{\alpha_{2}}$ there induces faster ${\text{G}}_{\alpha_{2}\beta \gamma }$ cycling. Since ${\text{G}}_{\alpha_{2}\beta \gamma }$ diffuses in the cytosol, this provides a potential redistribution of ${\text{G}}_{\alpha_{2}\beta \gamma }$ from the back to the front, which in turn results in more $G_{\alpha_{2}}^{*}$ at the front, thereby forming another positive feedback loop. These two positive feedback loops generate the symmetry breaking and signal amplification of Ras activation in a cAMP gradient.

We also studied cell responses to *g*_*α*2_ and ric8 mutations extensively. It is predicted in numerical simulations that in the presence of uniform stimuli, adaptation of Ras activity is perfect and the maximum cytosolic RBD depletion is reduced in *g*_*α*2_-null cells. In a cAMP gradient, *g*_*α*2_-null cells fail to sense directions and there is only an initial transient Ras activation. Adaptation of Ras activity is still imperfect in ric8-null cells, but the magnitude of imperfectness is reduced as compared with wild type cells. Moreover, simulations suggest that ric8-null cells fail to sense direction when they are exposed to a shallow gradient or a steep gradient with high mean concentration, highlight the importance of Ric8 in regulating Ras activation.

In contrast to LEGI-type models, the global diffusing G_*βγ*_ does not act as an inhibitor directly in our model—instead, it induces both activation and inhibition by activating RasGEF and RasGAP respectively. G_*βγ*_ also serves as a ‘global’ activator for the pool of RasGEF* and as a ‘global’ inhibitor by creating a uniform inhibition pool of RasGAP*. Asymmetry in their localization at the membrane arises from the fact that membrane-bound ${\text{G}}_{\alpha_{2}}^{*}$ recruits RasGEF from the cytosol, thereby creating an asymmetrical pool of RasGEF*. Hence, our model can be regarded as a local-global transitions of both excitation and inhibition with a delayed local sequestrations of excitation model, in the sense that initially both activation and inhibition go through a local-global transition due to diffusion of G_*β*γ_ while a delayed localized translocation by $G_{\alpha_{2}}^{*}$ contributes to a local excitation. Direction sensing is results from the G_*β*γ_- mediated,$G_{\alpha_{2}}$-Ric8 dependent signal transduction network.

Although the model is based on cAMP induced Ras activation in Dicty, GPCR-mediated Ras activation is highly conserved between Dicty and mammalian leukocytes [8]. GEF translocation via interaction with an upstream GTP-bound G protein is a principle conserved in evolution [47] and G_*α*_’s role in GPCR-mediated signalling has been emphasized in other systems [50, 75] and in drug discovery [76]. Therefore, our model could serve as a generic framework for GPCR mediated Ras activation in other systems and suggest new experiments in those systems.

## Materials and Methods

### The evolution equations for the reaction-diffusion model

We first formulate the reaction-diffusion system of signal transduction in general terms and then list the specific equations for the model.

Consider a bounded three dimensional domain Ω ⊂ *R*^3^ representing a cell, and denote ∂Ω as the plasma membrane. Then the reaction diffusion equation for a cytosolic species A is

$$\frac{\partial C}{\partial t}=\nabla \cdot \left(D\nabla C\right)+\sum_{i}s^{i}{\text{r}}^{i},$$ (2)

in which *C* = *C*(*t*, *x*) represents the concentration of A at time *t* at *x* ∈ Ω and *D* is the diffusion coefficient of A. The summation is a reaction term indicating *A* participates in cytosolic reactions which either depletes it or produces it. The *i*th reaction produces *s*^*i*^ molecules of A, or consumes −*s*^*i*^ > 0 molecules of A with a reaction rate r^*i*^ = r^*i*^(*t*, *x*). In the signal transduction network considered in this article, *s*^*i*^ = 0, 1.

The boundary conditions involve reactions on the boundary and binding and release of molecules at the membrane. We assume that the volume density *C* (the concentration in the cytosol) for A has the units *μM* and that the surface density (the concentration on the membrane), *C*_*m*_, has the units #/*μm*^2^. We also assume that the binding reactions at the membrane take place within a layer of thickness *δ*(*nm*) at the membrane. Then the net flux to the boundary, which can be positive or negative, can be written as

$$-\vec{n}\cdot D\nabla C=-D\frac{\partial C}{\partial n}=k^{+}\cdot \delta \cdot C-k^{-}\cdot C_{m}\equiv j^{+}-j^{-},$$ (3)

where $\vec{n}$ is the exterior unit normal to ∂Ω, *k*^±^ are the on and off rate of binding to the membrane, and *κ* = 602 relates the units of volume density and surface density scaled by Avogadro’s constant.

For the membrane form of species A we have the translocation-reaction-diffusion equation,

$$\frac{\partial C_{m}}{\partial t}=\nabla \cdot \left(D_{m}\nabla C\right)+\kappa \left(j^{+}-j^{-}\right)+\sum s_{m}^{i}{\text{r}}_{m}^{i},$$ (4)

where *C*_*m*_ = *C*_*m*_(*t*, *x*) denotes the concentration on the membrane and *D*_*m*_ is the surface diffusion coefficient [77, 78]. The first term represents the diffusion on the membrane, which we ignore throughout, and the second represents transolcation between cytosol and membrane, which could be absent if A is confined on the membrane, such as *Ras*, Ras*.

There may also be conservation laws for certain substances. If the substances are confined to the membrane we write

$$\int_{\partial \Omega }\sum_{i=1}^{n}A_{n}dS=A^{tot},$$ (5)

where *A*_*i*_s are the concentrations of different forms and *A*^*tot*^ represents the total amount in the cell. If the substances are present both in the cytosol and on the membrane, we write

$$\int_{\Omega }\sum_{i=1}^{k}A_{i}^{c}dx+\int_{\partial \Omega }\sum_{j=1}^{n}A_{j}^{m}dS=A^{tot},$$ (6)

where *A*_*i*_s are the concentrations of different forms in the cytosol and $A_{i}^{m}s$ are the concentrations of different forms on the membrane.

We are now ready to assemble the system of equations that constitute the full kinetic model in a given geometry Ω. We have to account for 6 cytosolic species in the system *G*_*αβγ*_, *c*, *G*_*βγ*, *c*_, *RasGEF*_*c*_, *RasGAP*_*c*_, *Ric*8_*c*_ and *RBD*_*c*_. The evolution can be described by a system of diffusion-translocation equations

$$\begin{array}{lll} \frac{\partial G_{\alpha \beta \gamma },c}{\partial t} & = & \nabla \cdot (D_{G_{\alpha \beta \gamma },c}\nabla G_{\alpha \beta \gamma })\\ \frac{\partial {\text{G}}_{\beta \gamma ,\text{c}}}{\partial t} & = & \nabla \cdot (D_{{\text{G}}_{\beta \gamma },c}\nabla {\text{G}}_{\beta \gamma })\\ \frac{\partial RasGEF_{c}}{\partial t} & = & \nabla \cdot (D_{RasGEF_{c}}\nabla RasGEF_{c})\\ \frac{\partial RasGAP_{c}}{\partial t} & = & \nabla \cdot (D_{RasGAP_{c}}\nabla RasGAP_{c})\\ \frac{\partial Ric8_{c}}{\partial t} & = & \nabla \cdot (D_{Ric8_{c}}\nabla Ric8_{c})\\ \frac{\partial RBD_{c}}{\partial t} & = & \nabla \cdot (D_{RBD_{c}}\nabla RBD_{c}) \end{array}$$

with the following conditions on ∂Ω,

$$\begin{array}{lll} D_{G_{\alpha \beta \gamma },c}\frac{\partial G_{\alpha \beta \gamma },c}{\partial n} & = & {\text{j}}_{1}\\ D_{{\text{G}}_{\beta \gamma ,\text{c}}}\frac{\partial {\text{G}}_{\beta \gamma ,\text{c}}}{\partial n} & = & {\text{j}}_{2}\\ D_{RasGEF_{c}}\frac{\partial RasGEF_{c}}{\partial n} & = & {\text{j}}_{5}-{\text{j}}_{6}\\ D_{RasGAP_{c}}\frac{\partial RasGAP_{c}}{\partial n} & = & {\text{j}}_{7}\\ D_{Ric8_{c}}\frac{\partial Ric8_{c}}{\partial n} & = & {\text{j}}_{3}-{\text{j}}_{4}\\ D_{RBD_{c}}\frac{\partial RBD_{c}}{\partial n} & = & {\text{j}}_{8}-{\text{j}}_{9} \end{array}$$

The species that evolve on the membrane are: R*, *G*_*αβγ*, *m*_, *G*_*βγ*, *m*_, $G_{\alpha }^{*}$, *G*_*α*_, *Ric*8_*m*_, *Ric*8*, *RasGEF*_*m*_, *RasGAP*_*m*_, RasGEF*, RasGAP*, *Ras*, Ras* and *RBD*_*m*_. The evolution equations for these are given by

$$\begin{array}{lll} \frac{\partial R*}{\partial t} & = & {\text{r}}_{1}\\ \frac{\partial {\text{G}}_{\alpha \beta \gamma \text{,}m}}{\partial t} & = & -\kappa {\text{j}}_{1}-{\text{r}}_{2}+{\text{r}}_{7}\\ \frac{\partial {\text{G}}_{\beta \gamma \text{,}m}}{\partial t} & = & -\kappa {\text{j}}_{2}+{\text{r}}_{2}-{\text{r}}_{7}\\ \frac{\partial G_{\alpha }^{*}}{\partial t} & = & {\text{r}}_{2}-{\text{r}}_{3}+{\text{r}}_{5}\\ \frac{\partial G_{\alpha }}{\partial t} & = & {\text{r}}_{3}-{\text{r}}_{5}-{\text{r}}_{7}\\ \frac{\partial Ric8_{m}}{\partial t} & = & -\kappa {\text{j}}_{3}+\kappa {\text{j}}_{4}-{\text{r}}_{4}+{\text{r}}_{6}\\ \frac{\partial Ric8*}{\partial t} & = & {\text{r}}_{4}-{\text{r}}_{6}\\ \frac{\partial RasGEF_{m}}{\partial t} & = & -\kappa {\text{j}}_{5}+\kappa {\text{j}}_{6}-{\text{r}}_{8}+{\text{r}}_{9}\\ \frac{\partial RasGAP_{m}}{\partial t} & = & -\kappa {\text{j}}_{7}-{\text{r}}_{10}+{\text{r}}_{11}\\ \frac{\partial RasGEF*}{\partial t} & = & {\text{r}}_{8}-{\text{r}}_{9}\\ \frac{\partial RasGAP*}{\partial t} & = & {\text{r}}_{10}-{\text{r}}_{11}\\ \frac{\partial Ras*}{\partial t} & = & {\text{r}}_{12}-{\text{r}}_{13}+{\text{r}}_{14}-{\text{r}}_{15}\\ \frac{\partial Ras}{\partial t} & = & -{\text{r}}_{12}+{\text{r}}_{13}-{\text{r}}_{14}-{\text{r}}_{15}\\ \frac{\partial RBD_{m}}{\partial t} & = & -\kappa {\text{j}}_{8}+\kappa {\text{j}}_{9} \end{array}$$

The following conservation laws are also imposed:

$$\int_{\partial \Omega }\left(R+R^{*}\right)ds=R^{t},$$ (7)

where *R*^*t*^ is the total amount of receptors.

$$\int_{\Omega }\left(G_{\alpha ,c}+{\text{G}}_{\beta \gamma ,c}+G_{\alpha \beta \gamma },c+G_{\alpha }^{*}\right)dx+\int_{\partial \Omega }\left(G_{\alpha }+{\text{G}}_{\beta \gamma ,m}+G_{\alpha \beta \gamma ,m}\right)ds=G_{\alpha \beta \gamma }^{t},$$ (8)

where $G_{\alpha \beta \gamma }^{t}$ is the total amount of heterotrimetric G protein, indicating the cell does not produce additional heterotrimetric G protein.

$$\int_{\Omega }RasGEF_{c}dx+\int_{\partial \Omega }\left(RasGEF_{m}+RasGEF^{*}\right)ds=RasGEF^{t}.$$ (9)

Similarly, for RasGAP

$$\int_{\Omega }RasGAP_{c}dx+\int_{\partial \Omega }\left(RasGAP_{m}+RasGAP^{*}\right)ds=RasGAP^{t}.$$ (10)

For Ras, we have

$$\int_{\partial \Omega }\left(Ras+Ras^{*}\right)ds=Ras^{t}.$$ (11)

### Parameters

The parameters involved in the Receptor module are taken from the literature. We estimated the parameters in the heterotrimeric G protein module from steady state analysis (SSA) of the spatially lumped model averaged from the spatially distributed model. The parameters in the Ras module are also estimated from SSA and time dynamics of Ras activation. The detailed estimation scheme is described in the supporting information (see section Parameter estimation in S1 text). We summarize the parameters in Table 3.

**Table 3:** Parameter values used in the model of Ras activation pathway.

Parameter	Value	Description	References
*r*	5 *μm*	Cell radius	[46]
*δ*	10 *nm*	Effective length for membrane reactions	[79]
*RasGEF*^*t*^	80000 #/cell	Total RasGEF molecules	[80, 81]
*RasGAP*^*t*^	80000 #/cell	Total RasGAP molecules	[80]
*Ras*^*t*^	300000 #/cell	Total Ras molecules on the membrane	[80]
$G_{\alpha \beta \gamma }^{t}$	300000 #/cell	Total heterotrimeric G protein molecules	[79, 80]
*R*^*t*^	80000#/cell	Total receptors on the membrane	[33, 82]
$D_{RasGEF}{}_{{}_{c}}$	30 *μm*^2^/*s*	Diffusion constant of RasGEF	[83]
$D_{RasGAP}{}_{{}_{c}}$	30 *μm*^2^/*s*	Diffusion constant of RasGAP	[83]
$D_{G_{\alpha \beta \gamma },}{}_{c}$	30 *μm*^2^/*s*	Diffusion constant of G_*αβγ*_	[83]
*D*_*βγ,c*_	30 *μm*^2^/*s*	Diffusion constant of G_*βγ*_	[83]
$D_{RBD}{}_{{}_{c}}$	30 *μm*^2^/*s*	Diffusion constant of *RBD*_*c*_	[83]
$D_{Ric8}{}_{{}_{c}}$	30 *μm*^2^/*s*	Diffusion constant of *Ric*8_*c*_	[83]
${\text{k}}_{1}^{+}$	5.6 (*μM*)^−1^ *s*^−1^	Average binding rate of cAMP to GPCR	[35, 43]
${\text{k}}_{1}^{-}$	1 *s*^−1^	Average unbind rate of cAMP-bound GPCR	[35, 82]
k_2_	0.02 (#/*μm*^2^)^−1^ *s*^−1^	G_*αβγ*_ dissociation rate by R*	Estimated from SSA and [43]
k_3_	1 *s*^−1^	$G_{\alpha }^{*}$ GTPase rate	[79]
k_4_	0.004 (#/*μm*^2^)^−1^ *s*^−1^	Ric8 activation rate on the membrane	
k_5_	0.2 (#/*μm*^2^)^−1^ *s*^−1^	*G*_*α*_ reactivation rate by *Ric*8*	
k_6_	1 *s*^−1^	*Ric*8* deactivation rate	
k_7_	0.0070 (#/*μm*^2^)^−1^ *s*^−1^	Reassociation rate of G_*α*_ and G_*βγ*, *m*_	Estimated from SSA and [43]
h_1_	1 *s*^−1^	Off rate of G_*αβγ*, *m*_	[45]
h_2_	3.9 × 10^2^ *s*^−1^	Translocation rate of G_*αβγ*_, *c*	Estimated from SSA
h_3_	1 *s*^−1^	Off rate of G_*βγ*, *m*_	Set the same as *G*_*αβγ*_
h_4_	3.9 × 10^2^ *s*^−1^	Translocation rate of G_*βγ*, *c*_	Estimated from SSA
h_5_	1 *s*^−1^	Off rate of *Ric*8_*m*_	Set the same as *G*_*αβγ*_
h_6_	1.6667*s*^−1^	Translocation rate of *Ric*8_*c*_	Estimated from SSA
h_7_	0.02(#/*μm*^2^)^−1^ *s*^−1^	Translocation rate of *Ric*8_*c*_ facilitated by $G_{\alpha }^{*}$	
k_8_	0.0004 (#/*μm*^2^)^−1^ *s*^−1^	RasGEF activation rate by G_*βγ*, *m*_	
k_9_	2*s*^−1^	RasGEF* deactivation rate	
k_10_	0.0001 (#/*μm*^2^)^−1^ *s*^−1^	RasGAP activation rate by G_*βγ*, *m*_	
k_11_	0.5 *s*^−1^	RasGAP* deactivation rate	
k_12_	0.11 (#/*μm*^2^)^−1^ *s*^−1^	Ras activation rate by *RasGEF*^*a*^	
k_13_	1 (#/*μm*^2^)^−1^ *s*^−1^	Ras* deactivation rate by *RasGAP*^*a*^	
k_14_	1.1 × 10^−7^ *s*^−1^	Spontaneous Ras activation rate	
k_15_	10^−6^ *s*^−1^	Spontaneous Ras* deactivation rate	
h_8_	1*s*^−1^	Off rate of *RasGEF*_*m*_	Set the same as PTEN [84]
h_9_	444.4*s*^−1^	Translocation rate of *RasGEF*_*c*_	Estimated from SSA
h_10_	2(#/*μm*^2^)^−1^ *s*^−1^	Translcation rate of *RasGEF*_*c*_ facilitated by $G_{\alpha }^{*}$	
h_11_	1*s*^−1^	Off rate of *RasGAP*_*m*_	Set the same as PTEN [84]
h_12_	444.4*s*^−1^	Translocation rate of *RasGAP*_*c*_	Estimated from SSA

## Supporting Information

S1 Text S1 Text provides extended analysis of the model and the parameter estimation schemes. (PDF)
