# Supplementary material for: A Model for Direction Sensing in Dictyostelium discoideum: Ras Activity and Symmetry Breaking Driven by a Gβγ-Mediated, Gα2-Ric8 -- Dependent Signal Transduction Network
Source: PLoS Comput Biol. 2016 May 6;12(5):e1004900. doi: 10.1371/journal.pcbi.1004900 (PMC4859573; doi:10.1371/journal.pcbi.1004900)
Supplement: S1 Text — (PDF) [file pcbi.1004900.s001.pdf]

Supporting Information

Other characteristics under uniform stimuli

Short vs Long saturating stimuli Our proposed network exhibits a maximal response to short saturating stimuli. RBD translocation is illustrated in Figure A when the cell is applied a short (2s) and a long (20s) saturating stimuli ($1\ \mu M$). Short and long stimuli lead to a response with the same rise time, the same peak and the same initial decline, although there is a additional slowly-declining phase for the longer stimulus. Similar experiments are reported in [1], in which it is suggested that this slowly declining phase could be related to the start of the secondary responses usually seen during continued stimulation [2, 3].

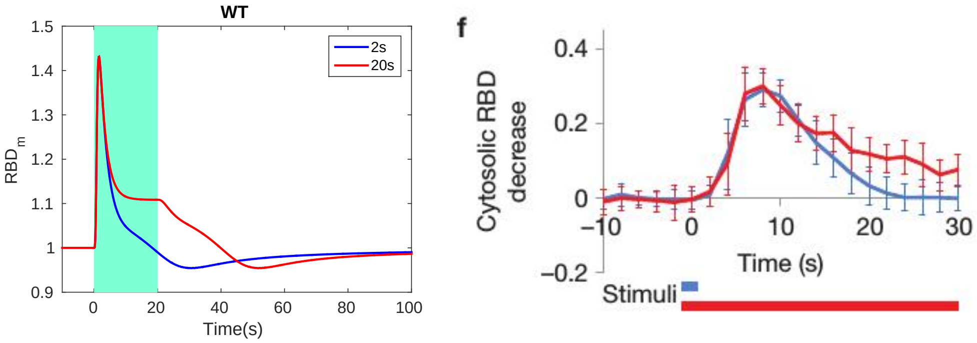

Fig A. Short stimulus vs long stimulus.
Left: Simulation; *Right*: Results reported in [1]. Blue indicates a 2s stimulus and red indicates a 20s stimulus.

In the model the ratio of $RasGEF^*$ and $RasGAP^*$ determines the Ras activation, and for saturating cAMP stimuli the ratio $RasGEF^*/RasGAP^*$ rises instantaneously and arrives at a maximum within 2 seconds, whereas it takes longer to achieve maximum RasGEF and RasGAP activation separately. Hence we observe a response with the same rise time and the same peak for short and long stimuli. In the latter case there is an additional slowly-declining phase due to the higher peak of $RasGEF^*$ and $RasGAP^*$. This indicates that subtle regulation of RasGEF and RasGAP activation at saturating cAMP is essential for the observed characteristics.

Cell responses under non-saturating cAMP stimuli We predict that the cell loses the ability to induce full responses by short stimuli and the existence of a refractory period greater than 12 seconds (which was the smallest time interval between stimuli used in [1]) at low cAMP level since temporal dynamics of RasGEF and RasGAP are much weaker, which is confirmed by simulation results shown in Fig. B where the cAMP level is reduced from $1\mu M$ to $1nM$. Indeed, longer stimulus induces higher peak of RBD_m and duration between stimuli no longer affects second response.

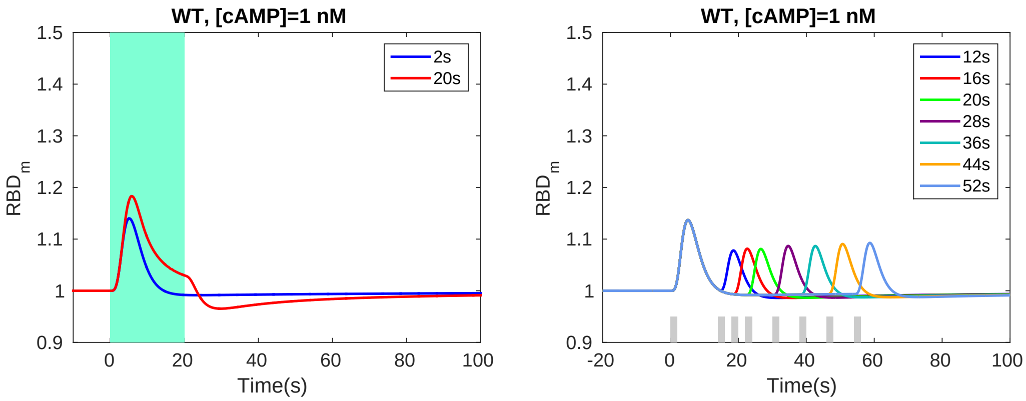

Fig B. The cell responses in a low non-saturating cAMP stimulus.
Left: time course of RBD_m when the cell is applied a 1 nM short stimulus and long stimulus; *Right*: time course of RBD_m to two 2s cAMP stimuli of 1 nM separated by increasing duration.

Rectification in $g\alpha_2$-null cells and ric8-null cells As seen from Figure C, the simulated g_{α_2}-null cells show a much larger response to termination of the stimulus, as shown in the middle panel. At 1 μM cAMP, RBD drops ∼15% below the prestimulus level, compared to < 5% in WT cells. Surprisingly, rectification is significantly reduced compared to that in WT cells, and even compared to g_{α_2}-null cells. To understand these behaviors, recall that $G_{\alpha_2\beta\gamma}$ re-association is increased in $ric8$-null cells because G_{α_2} activation is absent. As a result, the time dynamics of RasGEF and RasGAP are altered correspondingly (see Figure D), and consequently, Ras activation patterns are changed.

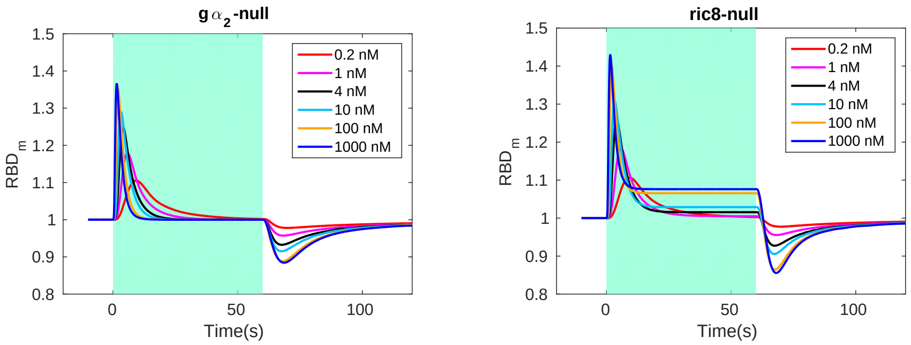

Fig C. Rectification in $g\alpha_2$-null cells(left) and ric8-null cells (right).

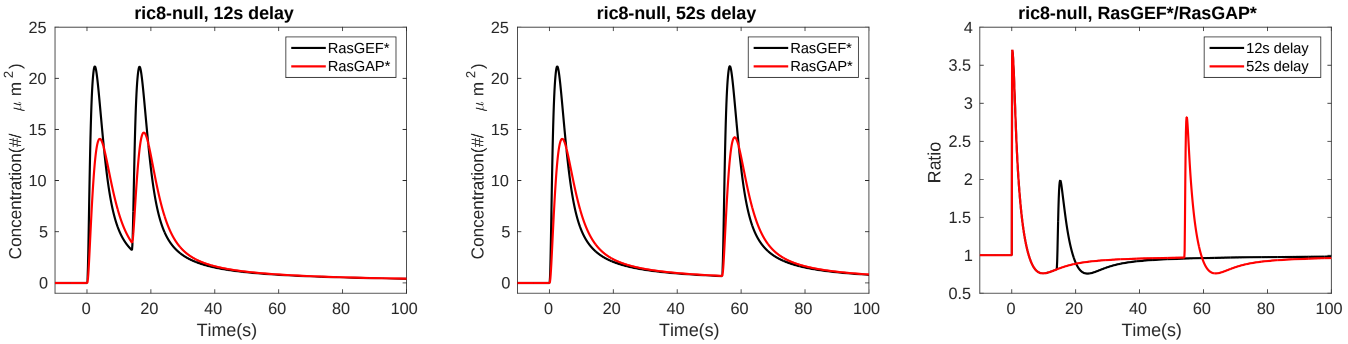

Fig D. The time course of $RasGEF^*$, $RasGAP^*$ and $RasGEF^*/RasGAP^*$ activities in $ric8$-null cells.

Other characteristics under a graded stimulus

Effects of diffusion

- Slow $G_{\alpha_2\beta\gamma}$ and Ric8 diffusion

The cell responds similarly when there is no apparent $G_{\alpha\beta\gamma}$ and Ric8 diffusion, as demonstrated in Fig. E and Fig. F. In both cases, Ras activation first occur at both ends, to reach a maximum, and then to decline to reach different steady-state levels in distinct parts of the cell. Only a shallow reactivation of Ras can be observed after it declines to a minimum, suggesting that symmetry breaking is strongly severed in the absence of $G_{\alpha\beta\gamma}$ and Ric8 diffusion.

These two simulations suggest that the two sources of signal amplification are equally important: The imbalanced sequestration of $G_{\alpha\beta\gamma}$ is sabotaged in the absence of $G_{\alpha\beta\gamma}$ diffusion and asymmetrical recruitments of Ric8 is destroyed in the absence of Ric8 diffusion. Therefore, the symmetry breaking phase collapses in either cases due to deficiency of signal amplification.

Our model reveals the importance of Ric8 in amplifying the signal at the level of Ras by regulating $G_{\alpha_2\beta\gamma}$ cycling: on one hand, it amplifies RasGEF activation at the front by reactivating $G\alpha 2$; on the other hand, it amplifies $G^*_{\alpha_2}$ activation by redistributing $G_{\alpha_2\beta\gamma}$ between the front and the rear of the cell.

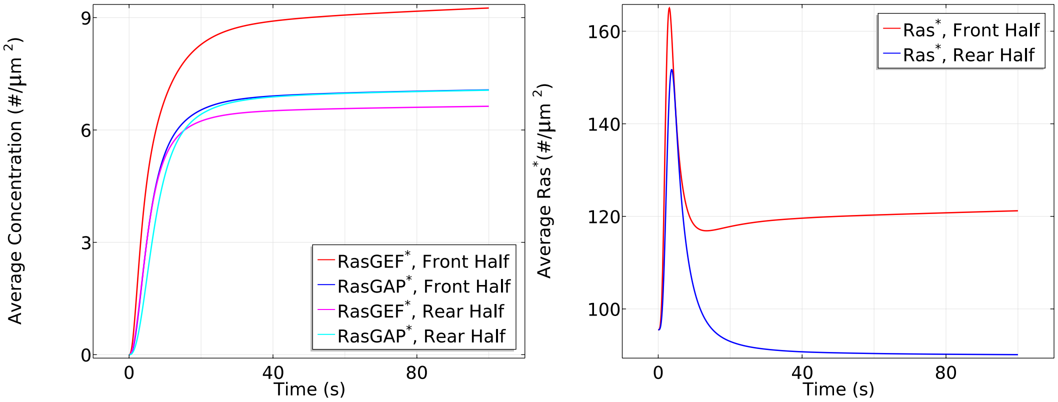

Fig E. The time course of average $RasGEF^*$ and $RasGAP^*$ (left), and Ras^* (right) in a cAMP gradient defined by $C_f = 10$ nM and C_r = 1nM in the absence of apparent $G_{\alpha\beta\gamma}$ diffusion.

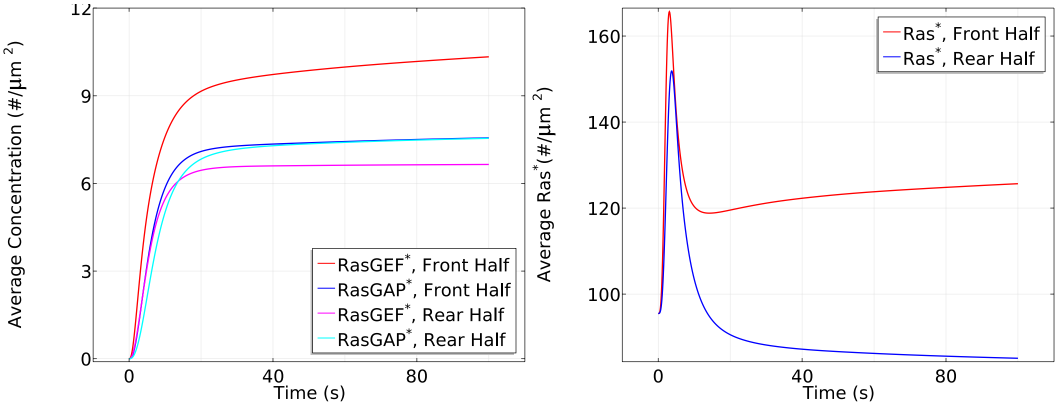

Fig F. The time course of average $RasGEF^*$ and $RasGAP^*$ (left), and Ras^* (right) in a cAMP gradient defined by $C_f = 10$ nM and C_r = 1nM in the absence of apparent Ric8 diffusion.

- Slow $G\beta\gamma$ and RasGEF diffusion

We also tested the cell response when both $G_{\beta\gamma}$ and RasGEF diffusion are absent (see Fig. G). Because there is no $G_{\beta\gamma}$ diffusion, the activity of $RasGAP^*$ is stronger at the front of the cell. Meanwhile, the supply of RasGEF and $G^*_{\alpha_2}$ facilitated asymmetrical recruitment of RasGEF is limited since RasGEF diffusion is absent. Therefore, we observe a higher Ras^* activity at the rear of the cell even though the cAMP gradient is opposite.

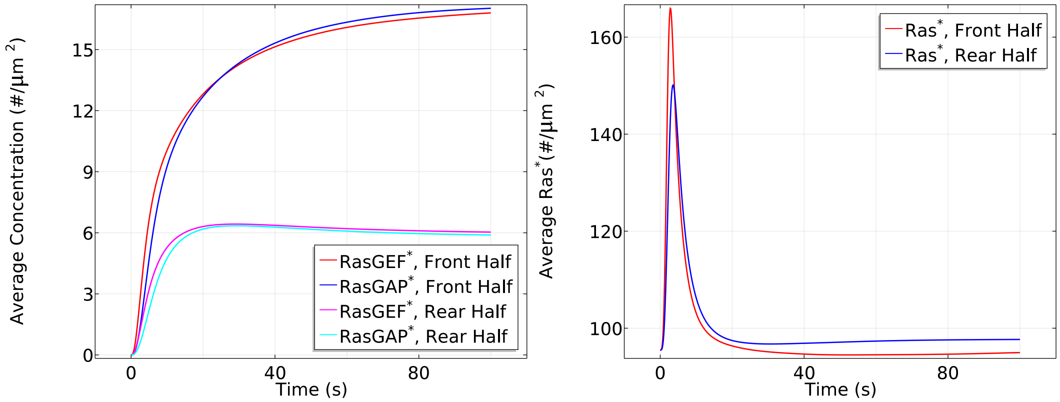

Fig G. The time course of average $RasGEF^*$ and $RasGAP^*$ (left), and Ras^* (right) in a cAMP gradient defined by $C_f = 10$ nM and C_r = 1nM in the absence of apparent $G_{\beta\gamma}$ and RasGEF diffusion.

Robustness of the $G\alpha_2$-$G\beta\gamma$-Ric8 triangle A schematic of the different modes is shown in Fig. H, which illustrates the importance of $G_{\beta\gamma}$ amd G_α.

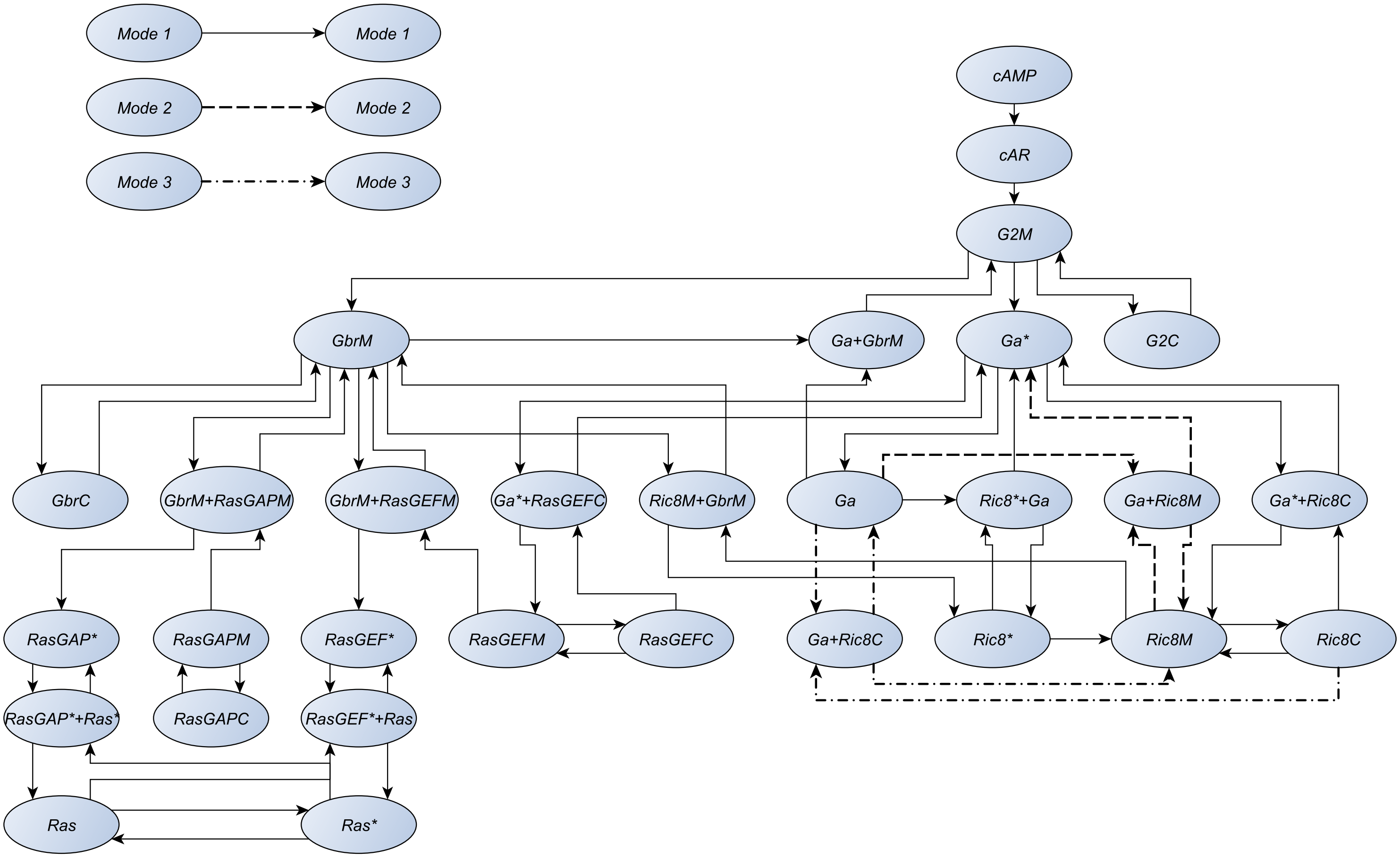

Fig H. A schematic of the detailed network topology.

Components end with M represents membrane species and components end with C represents cytosolic species. Gbr: $G_{\beta\gamma}$; Ga: G_{α}.

We tested three gradients in Mode 2 and the simulation results are illustrated in Fig. I and Fig. J.

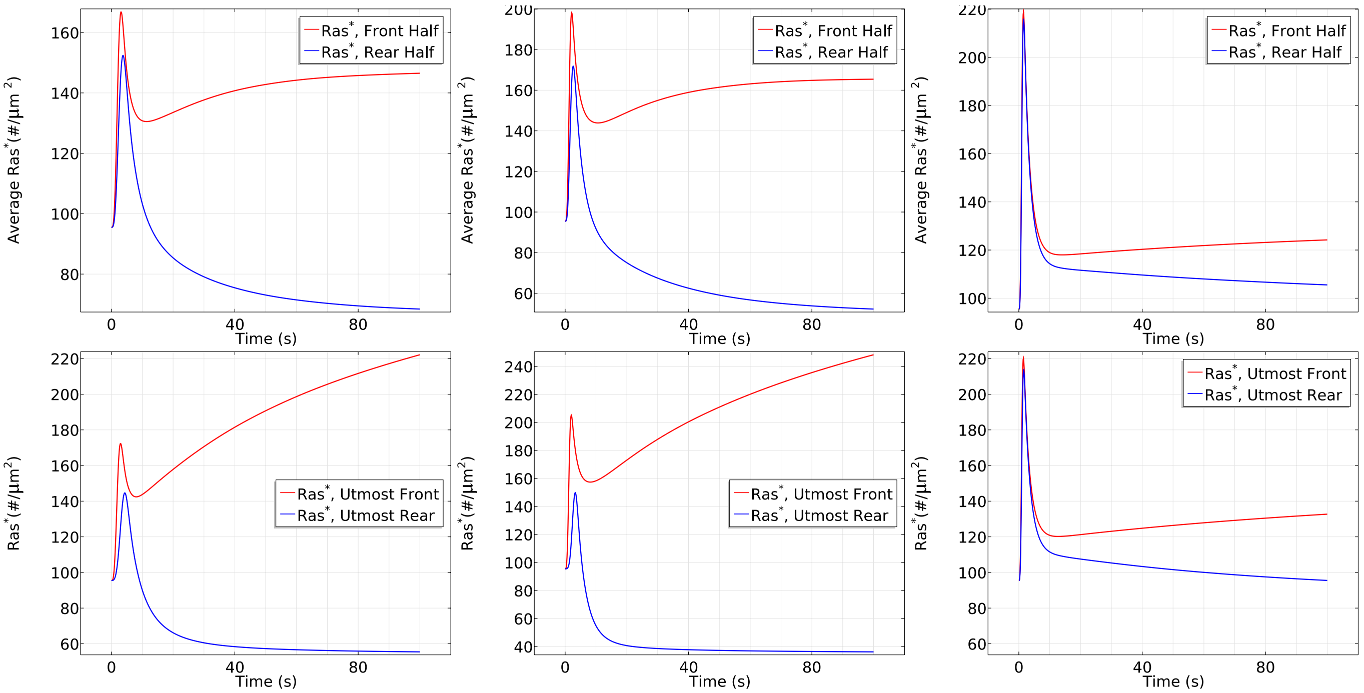

Fig I. The time course of Ras^* at the front and rear half (top) and at x_f and x_r (bottom) in various gradient in Mode 2.

Left: $C_f = 10$ nM and $C_r = 1$ nM. *Center*: $C_f = 50$ nM and $C_r = 0$ nM. *Right*: $C_f = 175$ nM and $C_r = 125$ nM.

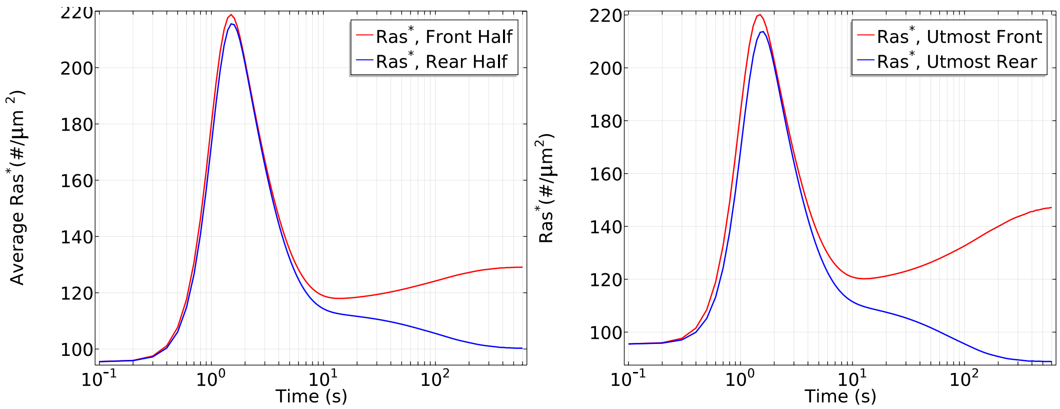

Fig J. The time course of Ras^* at the front and rear half (left) and at x_f and x_r (right) in a cAMP gradient in Mode 2 defined by $C_f = 175$ nM and $C_r = 125$ nM.

Fig. I and Fig. J demonstrate that Mode 2 still capture the basic characteristics of Ras activation, almost the same as Mode 1 except the magnitudes are slightly changed. This suggests that the robustness of the network and $G_{\beta\gamma}$ activation is not an essential step.

Next, we test the possibility that Membrane recruitment of Ric8 is promoted by $G\alpha$ (Mode 3). The results are illustrated in Fig. K and Fig. L. These plots suggest that the cell is still able to sense direction and exhibit biphasic responses under various cAMP gradients. They differ from the plots in Mode 1 and Mode 2 in the sense the point Ras activity equilibrates quicker and the magnitudes of the symmetry breaking are smaller.

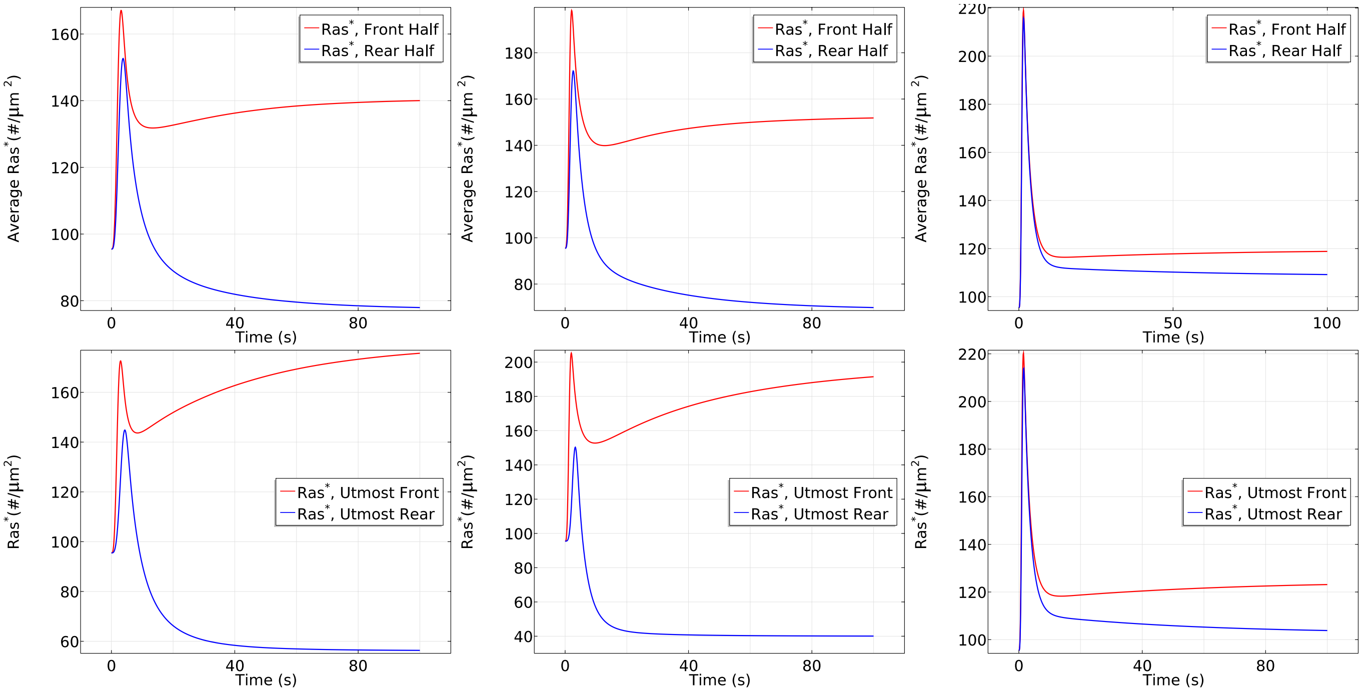

Fig K. The time course of Ras^* at the front and rear half (top) and at x_f and x_r (bottom) in various gradient in Mode 3.

Left: $C_f = 10$ nM and $C_r = 1$ nM. *Center*: $C_f = 50$ nM and $C_r = 0$ nM. *Right*: $C_f = 175$ nM and $C_r = 125$ nM.

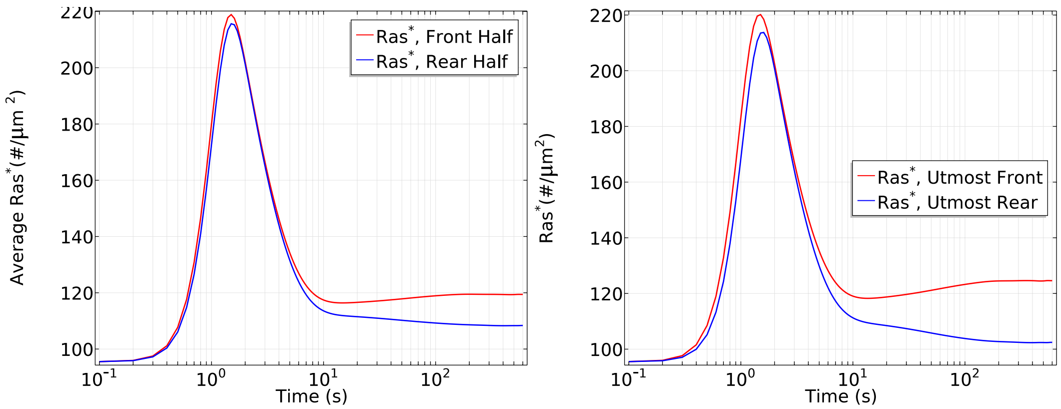

Fig L. The time course of Ras^* at the front and rear half (left) and at x_f and x_r (right) in a cAMP gradient in Mode 3 defined by $C_f = 175$ nM and $C_r = 125$ nM.

Spatial oscillations during gradient switch

To illustrate the spatio-temporal complexity in Ras redistribution during gradient switch, we plot the spatial profile of Ras^* for the intermediate gradient switch (from 0 - 100 nM to 75 -25 nM) at three great circles of the sphere, as shown in Fig. M. At $t = 180$ s (80 seconds after the gradient switch), we can clearly observe the spatial oscillations of Ras activation. Along x-axis from $x = -5\mu m$ to $x = 5\mu m$, we see a High-Low-High-Low profile. The first high profile is due to the formation of the new front after gradient switch, the next low and hight profiles correspond to the left-over of the old back and the old front respectively. Finally, the last low profile is a representation of the formation of the new back.

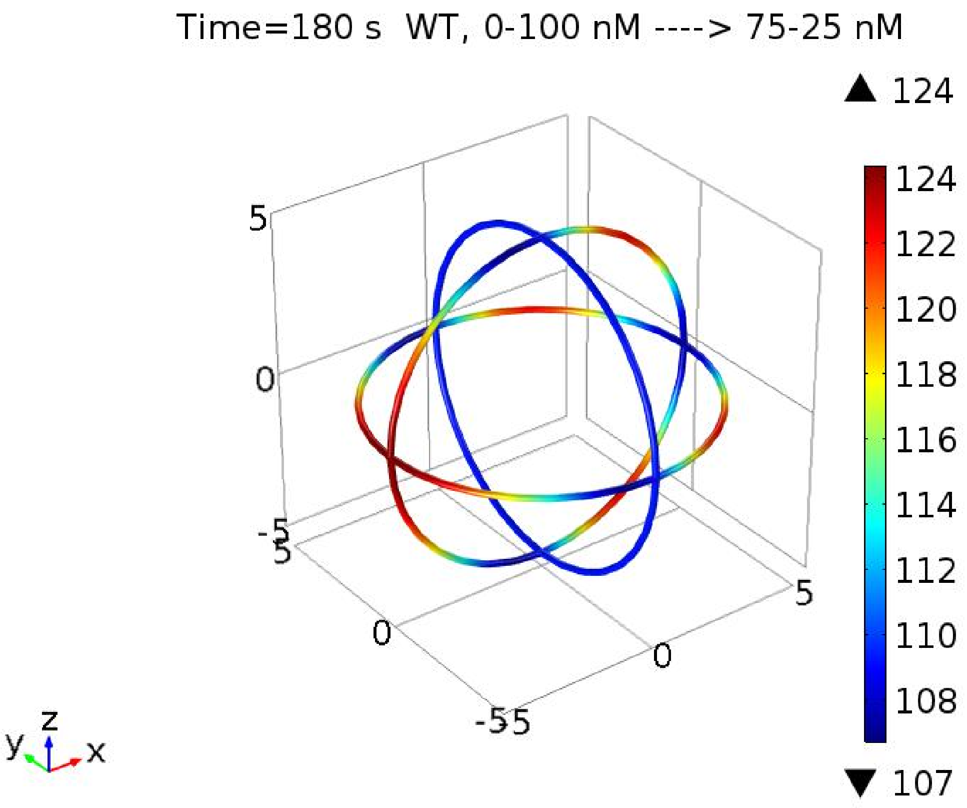

Fig M. The spatial profile of Ras^* at three great circles of the sphere at $t = 180$ s.

Reaction Rates

The reactions considered in signal transduction are mostly bimolecular reactions:

$$\mathcal{R} : A + B \to C,$$

and we model the reaction rate as

$$\mathrm{r} = kA \cdot B,$$

where k is the reaction rate and A, B represent the concentrations of A and B.

Reactions on the membrane and membrane-cytosol translocation are expressed in the form

$$A + B \to A + B^*. \tag{1}$$

To model this kind of reactions in a realistic way, we first write the full dynamics

$$A + B \underset{k_{-1}}{\overset{k_1}{\rightleftharpoons}} \overline{A \cdot B} \xrightarrow{k_2} \overline{A \cdot B^*} \xrightarrow{k_3} A + B^*,$$

then the full dynamics can be described by

$$\frac{dA}{dt} = -k_1 AB + k_{-1}\overline{A \cdot B} + k_3\overline{A \cdot B^*} \tag{2}$$

$$\frac{d\overline{A \cdot B}}{dt} = k_1 AB - k_{-1}\overline{A \cdot B} - k_2\overline{A \cdot B} \tag{3}$$

$$\frac{d\overline{A \cdot B^*}}{dt} = k_2\overline{A \cdot B} - k_3\overline{A \cdot B^*} \tag{4}$$

$$\frac{dB^*}{dt} = k_3\overline{A \cdot B^*}. \tag{5}$$

Now we assume that fast relaxation to a steady state for the intermediate enzyme-substrate complexes is achieved,

$$\frac{d\overline{A\cdot B}}{dt} = \frac{d\overline{A\cdot B^*}}{dt} = 0.$$

Hence

$$k_1 AB - k_{-1}\overline{A\cdot B} = k_2\overline{A\cdot B} = k_3\overline{A\cdot B^*}.$$

Therefore,

$$\overline{A\cdot B} = \frac{k_1}{k_{-1}+k_2}AB.$$

Then we have

$$\begin{aligned}\frac{dA}{dt} &= -k_1 AB + k_{-1}\overline{A\cdot B} + k_3\overline{A\cdot B^*}\\ &= -k_2\overline{A\cdot B} + k_3\overline{A\cdot B^*}\\ &= -k_3\overline{A\cdot B^*} + k_3\overline{A\cdot B^*}\\ &= 0,\end{aligned}$$

and

$$\frac{dB^*}{dt} = k_3\overline{A\cdot B^*} \tag{6}$$

$$= k_2\overline{A\cdot B} \tag{7}$$

$$= \frac{k_1 k_2}{k_{-1}+k_2}AB. \tag{8}$$

Denote

$$K = \frac{k_1 k_2}{k_{-1}+k_2},$$

we obtain the reaction rate of (1)

$$\frac{dB^*}{dt} = KAB. \tag{9}$$

Parameter estimation

We first show that a spatially lumped model can be derived by mean approximation of the generic reaction diffusion system. The spatially lumped model will be used to analyse the adaptation of Ras activity under uniform stimulation.

Define the mean concentration $\overline{C}$ of a given species A in the cytosol Ω to be

$$\overline{C}(t) = \frac{1}{|\Omega|}\int_\Omega C(t,x)dx,$$

where $|\Omega|$ is the volume of the cytosol, or the volume of the cell.

Integrating both sides of the reaction diffusion equation, we obtain by the divergence theorem,

$$\begin{aligned}\frac{d\overline{C}}{dt} &= \frac{1}{|\Omega|}\int_\Omega \frac{\partial C(t,x)}{\partial t}dx\\ &= \frac{1}{|\Omega|}\int_\Omega \nabla\cdot(D\nabla C)dx + \frac{1}{|\Omega|}\int_\Omega \sum_i s^i \mathrm{r}^i dx\\ &= \frac{1}{|\Omega|}\int_{\partial\Omega} D\nabla C\cdot n ds + \sum_i s^i \frac{1}{|\Omega|}\int_\Omega \mathrm{r}^i dx\\ &= \frac{1}{|\Omega|}\int_{\partial\Omega} D\frac{\partial C}{\partial n}ds + \sum_i s^i\overline{\mathrm{r}^i},\end{aligned} \tag{10}$$

in which the average reaction rates are defined as

$$\overline{\mathrm{r}^i} = \frac{1}{|\Omega|}\int_\Omega \mathrm{r}^i dx.$$

Substitute the boundary conditions into (10), we have

$$\frac{d\overline{C}}{dt} = \frac{1}{|\Omega|}\int_{\partial\Omega} \left(-j^+ + j^-\right) ds + \sum_i s^i \overline{\mathrm{r}^i}. \tag{11}$$

If we assume that j^+ and j^- are space invariant, (11) can be simplified as

$$\frac{d\overline{C}}{dt} = \frac{|\partial\Omega|}{|\Omega|}\left(-j^+ + j^-\right) + \sum_i s^i \overline{\mathrm{r}^i}, \tag{12}$$

where $|\partial\Omega|$ is the surface area of the cell membrane. If the cell has a spherical shape, then

$$\frac{|\partial\Omega|}{|\Omega|} = \frac{r}{3}.$$

Since the membrane diffusion is small compared to diffusion in the cytosol, we omit the membrane diffusion for simplicity, and similarly, define the mean concentration $\overline{C_m}$ of a given species A on the membrane $\partial\Omega$ to be

$$\overline{C_m}(t) = \frac{1}{|\partial\Omega|}\int_{\partial\Omega} C_m(t,x) ds,$$

and integrate both sides of the translocation reaction equation, we obtain

$$\begin{aligned}
\frac{d\overline{C_m}(t)}{dt} &= \frac{1}{|\partial\Omega|}\int_{\partial\Omega} \frac{\partial C_m(t,x)}{\partial t} ds \\
&= -\kappa \frac{1}{|\partial\Omega|}\int_{\partial\Omega} \left(j^+ - j^-\right) ds + \sum_i s_m^i \frac{1}{|\partial\Omega|}\int_{\partial\Omega} \mathrm{r}_m^i ds \\
&= -\kappa\left(j^+ - j^-\right) + \sum_i s_m^i \overline{\mathrm{r}_m}^i,
\end{aligned} \tag{13}$$

where

$$\overline{\mathrm{r}_m}^i = \frac{1}{|\partial\Omega|}\int_{\partial\Omega} \mathrm{r}_m^i ds.$$

If we assume the parameters and the concentrations on the membrane are spatially invariant, the parameters in the spatially distributed model and the spatially lumped model are identical in the sense that

$$\begin{aligned}
\overline{\mathrm{r}_m}^i &= \frac{1}{|\partial\Omega|}\int_{\partial\Omega} \mathrm{r}_m^i ds \\
&= \frac{1}{|\partial\Omega|}\int_{\partial\Omega} kABds \\
&= kAB.
\end{aligned}$$

Through mean approximation, we obtain a spatially lumped model consisting of equations in the form (11) and (13).

Now we explain how we the parameters are estimated using the spatially lumped model and steady state analysis

(SSA). The spatially lumped model for the G protein module is given by

$$\frac{dG_{\alpha\beta\gamma,m}}{dt} = \mathrm{h}_2 G_{\alpha\beta\gamma}, c - \mathrm{h}_1 G_{\alpha\beta\gamma,m} - \mathrm{k}_2 G_{\alpha\beta\gamma,m} R^* + \mathrm{k}_7 G_\alpha \cdot \mathrm{G}_{\beta\gamma,m} \tag{14}$$

$$\frac{dG_{\alpha\beta\gamma}, c}{dt} = \frac{3}{r}(-\mathrm{h}_2 G_{\alpha\beta\gamma}, c + \mathrm{h}_1 G_{\alpha\beta\gamma,m}) \tag{15}$$

$$\frac{dG^*_\alpha}{dt} = \mathrm{k}_2 G_{\alpha\beta\gamma,m} R^* - \mathrm{k}_3 G^*_\alpha + \mathrm{k}_5 Ric8^* G_\alpha \tag{16}$$

$$\frac{dG_\alpha}{dt} = \mathrm{k}_3 G^*_\alpha - \mathrm{k}_7 G_\alpha \cdot \mathrm{G}_{\beta\gamma,m} - \mathrm{k}_5 Ric8^* G_\alpha \tag{17}$$

$$\frac{d\mathrm{G}_{\beta\gamma,m}}{dt} = -\mathrm{h}_3 \mathrm{G}_{\beta\gamma,m} + \mathrm{h}_4 \mathrm{G}_{\beta\gamma,c} - \mathrm{k}_7 G_\alpha \cdot \mathrm{G}_{\beta\gamma,m} + \mathrm{k}_2 G_{\alpha\beta\gamma,m} R^* \tag{18}$$

$$\frac{d\mathrm{G}_{\beta\gamma,c}}{dt} = \frac{3}{r}(\mathrm{h}_3 \mathrm{G}_{\beta\gamma,m} - \mathrm{h}_4 \mathrm{G}_{\beta\gamma,c}) \tag{19}$$

$$\begin{aligned}\frac{dRic8_m}{dt} &= -\mathrm{h}_5 Ric8_m + \mathrm{h}_6 Ric8_c - \mathrm{k}_4 Ric8_m \cdot \mathrm{G}_{\beta\gamma,m} + \mathrm{k}_6 Ric8^* \\ &\quad + \mathrm{h}_7 \delta G^*_\alpha \cdot Ric8_c\end{aligned} \tag{20}$$

$$\frac{dRic8_c}{dt} = \frac{3}{r}(\mathrm{h}_5 Ric8_m - \mathrm{h}_6 Ric8_c - \mathrm{h}_7 \delta G^*_\alpha \cdot Ric8_c) \tag{21}$$

$$\frac{dRic8^*}{dt} = \mathrm{k}_4 Ric8_m \cdot \mathrm{G}_{\beta\gamma,m} - \mathrm{k}_6 Ric8^*. \tag{22}$$

At steady state, from (15), we have

$$\frac{\mathrm{h}_2}{\mathrm{h}_1} = \frac{G_{\alpha\beta\gamma,m}}{\delta G_{\alpha\beta\gamma}, c}.$$

It is reported in [4] that roughly 30% of the heterotrimeric G protein is in the cytosol, hence

$$\frac{G_{\alpha\beta\gamma,m} S}{G_{\alpha\beta\gamma}, cV} = \frac{7}{3}.$$

This is true at all stimulus level. When no stimulus presents, we have

$$G^t_{\alpha\beta\gamma,m} = 0.7 G^t_{\alpha\beta\gamma}/S, G_{\alpha\beta\gamma}\beta^t_c = 0.3 G^t_{\alpha\beta\gamma}/V.$$

Also,

$$\mathrm{h}_2 = \frac{7V}{3S\delta}\mathrm{h}_1, S = 4\pi r^2, V = 4/3\pi r^3.$$

[4] measures the recovery rate for the G proteins in fluorescence recovery after photobleaching (FRAP) was independent of the amount of bleached area with a half-time of approximately 5 seconds. Hence we estimate $\mathrm{h}_1 = 1s^{-1}$, which value is also assigned for h_3, h_5, h_9 and h_{11}.

It is reported in [5], half of the G protein dissociates at cAMP concentration $10nM$. Assume p represents the ratio of quantities of $\mathrm{G}_{\beta\gamma,c}$ and $\mathrm{G}_{\beta\gamma,m}$ at this cAMP concentration level, then by (19), we have

$$\frac{\mathrm{h}_4}{\mathrm{h}_3} = \frac{\mathrm{G}_{\beta\gamma,m}}{\mathrm{G}_{\beta\gamma,c}} = \frac{V}{p\delta S}.$$

We speculate that $\mathrm{G}_{\beta\gamma,m}$ dissociates from the membrane with the same rate of $\mathrm{G}\alpha_{\beta\gamma,m}$, $\mathrm{h}_3 = \mathrm{h}_1$, and then h_4 can be calculated. In the numerical simulations we assign $p = 3/7$.

From conservation of G protein, at $10nM$ cAMP concentration, we have

$$G_{\alpha\beta\gamma,m} S + G_{\alpha\beta\gamma}, cV + \mathrm{G}_{\beta\gamma,m} S + \mathrm{G}_{\beta\gamma,c} V = G^t_{\alpha\beta\gamma}, \tag{23}$$

and

$$G_{\alpha\beta\gamma,m} S + G_{\alpha\beta\gamma}, cV + G^*_\alpha S + G_\alpha S = G^t_{\alpha\beta\gamma}. \tag{24}$$

Note that

$$G_{\alpha\beta\gamma,m}S + G_{\alpha\beta\gamma}, cV = \frac{1}{2}G^t_{\alpha\beta\gamma},$$

we have

$$\mathrm{G}_{\beta\gamma,m}S + \mathrm{G}_{\beta\gamma,c}V = G^*_\alpha S + G_\alpha S = \frac{1}{2}G^t_{\alpha\beta\gamma}. \tag{25}$$

From (22) and (25), we obtain

$$\mathrm{G}_{\beta\gamma} = \frac{\frac{1}{2}G^t_{\alpha\beta\gamma}}{S + pV} = \frac{\mathrm{k}_6 Ric8^*}{\mathrm{k}_4 Ric8_m}, \tag{26}$$

which leads to

$$Ric8^* = \alpha Ric8_m, \alpha = \frac{\frac{1}{2}G^t_{\alpha\beta\gamma}}{S + pV} \cdot \frac{\mathrm{k}_4}{\mathrm{k}_6}. \tag{27}$$

Also, from (25),

$$G^*_\alpha + G_\alpha = \frac{\frac{1}{2}G^t_{\alpha\beta\gamma}}{S}. \tag{28}$$

Moreover, from (21), we have

$$G^*_\alpha = \frac{\mathrm{h}_5 Ric8_m - \mathrm{h}_6 \delta Ric8_c}{\mathrm{h}_7 \delta Ric8_c}. \tag{29}$$

From (29) and (16)

$$G_\alpha = \frac{\mathrm{k}_3 \frac{\mathrm{h}_5 Ric8_m - \mathrm{h}_6 \delta Ric8_c}{\mathrm{h}_7 \delta Ric8_c} - \mathrm{k}_2 G_{\alpha\beta\gamma,m} R^*}{\mathrm{k}_5 Ric8_a}. \tag{30}$$

Substitute (29) and (30) into (28), we obtain a equation consisting of $Ric8_c$, Ric_m and $Ric8^*$,

$$\frac{\mathrm{h}_5 Ric8_m - \mathrm{h}_6 \delta Ric8_c}{\mathrm{h}_7 \delta Ric8_c} + \frac{\mathrm{k}_3 \frac{\mathrm{h}_5 Ric8_m - \mathrm{h}_6 \delta Ric8_c}{\mathrm{h}_7 \delta Ric8_c} - \mathrm{k}_2 G_{\alpha\beta\gamma,m} R^*}{\mathrm{k}_5 Ric8_a} = \frac{G_{\alpha\beta\gamma_t}}{2S}. \tag{31}$$

By conservation of total Ric8 and (27), we have

$$Ric8_c V + (Ric8_m + \alpha Ric8_m)S = Ric8_t,$$

from which we have

$$Ric8_c = \beta + \gamma Ric8_m, \beta = \frac{Ric8_t}{V}, \gamma = -\frac{(1+\alpha)S}{V} < 0. \tag{32}$$

Substitute (32) and (27) into (31), we obtain a equation of $Ric8_m$, which can be simplified into a quadratic equation

$$a \cdot (Ric8_m)^2 + b \cdot Ric8_m + c = 0, \tag{33}$$

where

$$a = \alpha(\mathrm{k}_5\mathrm{h}_5 - \mathrm{k}_5\mathrm{h}_6\delta\gamma - C_2 C4\gamma),$$

$$b = -\alpha\mathrm{k}_5\mathrm{h}_6\delta\beta + \mathrm{k}_3\mathrm{h}_5 - C_3\gamma - C_2 C_4 \alpha\beta, c = -C_3\beta,$$

$$C_1 = \mathrm{k}_2 G_{\alpha\beta\gamma,m} R^*, C_2 = \frac{G_{\alpha\beta\gamma_t}}{2S},$$

and

$$C_3 = \mathrm{k}_3\mathrm{h}_6\delta + C_1\mathrm{h}_7\delta, C_4 = \mathrm{k}_5\mathrm{h}_7\delta.$$

Finally, by solving (33), we have

$$Ric8_m = \frac{-b + \sqrt{b^2 - 4ac}}{2a}.$$

Then we can calculate backward to get G_α, and estimate

$$\mathrm{k}_7 = \frac{C_1}{G_\alpha \mathrm{G}_{\beta\gamma,m}}.$$

Imperfect adaptation

The spatially lumped model for the RasGTPase module is given by

$$\frac{dRasGEF_c}{dt} = \frac{3}{r}(-\mathrm{h}_9\delta RasGEF_c + \mathrm{h}_8 RasGEF_m - \delta \mathrm{h}_{10} G_\alpha^* \cdot RasGEF_c) \tag{34}$$

$$\frac{dRasGAP_c}{dt} = \frac{3}{r}(-\mathrm{h}_{12}\delta RasGAP_c + \mathrm{h}_{11} RasGAP_m) \tag{35}$$

$$\begin{aligned}\frac{dRasGEF_m}{dt} &= \mathrm{h}_9 RasGEF_c - \mathrm{h}_8 RasGEF_m \\ &+ \mathrm{h}_{10} G_\alpha^* \cdot RasGEF_c - \mathrm{k}_8 \mathrm{G}_{\beta\gamma,m} \cdot RasGEF_m + \mathrm{k}_9 RasGEF^*\end{aligned} \tag{36}$$

$$\begin{aligned}\frac{dRasGAP_m}{dt} &= \mathrm{h}_{12} RasGAP_c - \mathrm{h}_{11} RasGAP_m - \mathrm{k}_{10} \mathrm{G}_{\beta\gamma,m} \cdot RasGAP_m \\ &+ \mathrm{k}_{11} RasGAP*\end{aligned} \tag{37}$$

$$\frac{dRasGEF^*}{dt} = \mathrm{k}_8 \mathrm{G}_{\beta\gamma,m} \cdot RasGEF_m - \mathrm{k}_9 RasGEF^* \tag{38}$$

$$\frac{dRasGAP^*}{dt} = \mathrm{k}_{10} \mathrm{G}_{\beta\gamma,m} \cdot RasGAP_m - \mathrm{k}_{11} RasGAP^* \tag{39}$$

$$\frac{dRas^*}{dt} = \mathrm{k}_{12} RasGEF^* \cdot Ras - \mathrm{k}_{13} RasGAP^* \cdot Ras^* + \mathrm{k}_{14} Ras - \mathrm{k}_{15} Ras^*. \tag{40}$$

At steady states, we have

$$RasGEF^* = \frac{\mathrm{k}_8 \mathrm{G}_{\beta\gamma,m} \cdot RasGEF_m}{\mathrm{k}_9}, RasGEF_c = \frac{\mathrm{h}_8 RasGEF_m}{\mathrm{h}_9\delta + \mathrm{h}_{10} G_\alpha^*}.$$

From the conservation law for RasGEF,

$$RasGEF_c V + RasGEF_m S + RasGEF^* S = RasGEF^t,$$

we obtain

$$\begin{aligned}RasGEF_m = \frac{RasGEF^t}{S} \times \\ \frac{\mathrm{k}_9(\mathrm{h}_9\delta + \mathrm{h}_{10} G_\alpha^*)}{(r/3\mathrm{h}_8 + \mathrm{h}_9\delta)\mathrm{k}_9 + \mathrm{h}_{10}\mathrm{k}_9 G_\alpha^* + \mathrm{h}_9\delta \mathrm{k}_8 \mathrm{G}_{\beta\gamma,m} + \mathrm{h}_{10}\mathrm{k}_8 \mathrm{G}_{\beta\gamma,m} \cdot G_\alpha^*}.\end{aligned} \tag{41}$$

Similarly, from steady states and conservation of RasGAP, we have

$$RasGAP_m = \frac{RasGAP^t}{S} \frac{\mathrm{k}_{11}\mathrm{h}_{12}\delta}{\mathrm{k}_{11}(r/3\mathrm{h}_{11} + \mathrm{h}_{12}\delta) + \mathrm{k}_{10}\mathrm{h}_{12}\delta \mathrm{G}_{\beta\gamma,m}}.$$

Then

$$f = \frac{Ras^*}{Ras^*} = \frac{\mathrm{k}_{12} RasGEF^*}{\mathrm{k}_{13} RasGAP*} = \frac{\mathrm{k}_{12}}{\mathrm{k}_{13}} \frac{\mathrm{k}_8}{\mathrm{k}_9} \frac{\mathrm{k}_{11}}{\mathrm{k}_{10}} \frac{RasGEF_m}{RasGAP_m}.$$

We impose

$$\mathrm{h}_{12} = \mathrm{h}_9, \mathrm{h}_{11} = \mathrm{h}_8,$$

and

$$\frac{\mathrm{k}_9}{\mathrm{k}_{11}} = \frac{\mathrm{k}_8}{\mathrm{k}_{10}} = \theta,$$

then there exists perfect adaptation when $\mathrm{h}_{10} = 0$. But when $\mathrm{h}_{10} \neq 0$,

$$\frac{\partial f}{\partial \mathrm{G}_{\beta\gamma,m}} \neq 0, \frac{\partial f}{\partial G_\alpha^*} \neq 0,$$

which means adaptation can not be perfect.

To determine the values of $h_{11}(h_8)$ and $h_{12}(h_9)$, we use steady state of equation (35)

$$\frac{h_{12}}{h_{11}} = \frac{RasGAP_m}{\delta RasGAP_c}.$$

If a partition of $RasGAP_c$ and $RasGAP_m$ is determine, we can calculate $h_{12}(h_8)$ based on $h_{11}(h_9)$. In the numerical simulation, we choose the ratio as

$$\frac{RasGAP_c V}{RasGAP_m S} = \frac{3}{7},$$

the same as $G_{\alpha_2\beta\gamma}$ partition without further information. The simulation results do not change significantly by varying this ratio.

We assign $h_{11} = 1s^{-1}$ based on a measurement of PTEN dissociation rate in [6]. We speculate that PTEN and RasGEF and RasGAP share similar time constants, since no explicit values for RasGEF and RasGAP dissociation are available.

To ensure Ras is activated when $G_{\beta\gamma}$ both activates $RasGEF$ and $RasGAP$, we require

$$\theta > 1.$$

In the simulation, we choose $\theta = 4$. Varying this value would only change the peak value of Ras activation.

Note that when $h_{10} = 0$, perfect adaptation gives us

$$\frac{Ras^*}{Ras} = \frac{k_{12}}{k_{13}} \frac{RasGEF^t}{RasGAP^t}.$$

In simulation we assign the ratio of Ras^* and Ras as $1:9$, whose value does not alter the system behaviors.

References

1. Huang CH, Tang M, Shi C, Iglesias PA, Devreotes P. An excitable signal integrator couples to an idling cytoskeletal oscillator to drive cell migration. Nat Cell Biol. 2013;15:1307–1316.

2. Chen L, Janetopoulos C, Huang YE, Iijima M, Borleis JA, Devreotes P. Two phases of actin polymerization display different dependencies on PI(3,4,5)P3 accumulation and have unique roles during chemotaxis. Mol Biol Cell. 2003;14:5028–5037.

3. Postma M, Roelofs J, Goedhart J, Gadella TWJ, Visser AJWG, van Haastert PJM. Uniform cAMP stimulation of dictyostelium cells Induces localized patches of signal transduction and pseudopodia. Mol Biol Cell. 2003;14:5019–5027.

4. Elzie CA, Colby J, Sammons MA, Janetopoulos C. Dynamic localization of G proteins in Dictyostelium discoideum. J Cell Bio. 2009;122:2597–2603.

5. Janetopoulos C, Jin T, Devreotes P. Receptor-mediated activation of heterotrimeric G-proteins in living cells. Science. 2001;291:2408–2411.

6. Vazquez F, Masuoka S, Sellers WR, Yanagida T, Ueda M, Devreotes P. Tumor suppressor PTEN acts through dynamic interaction with the plasma membrane. Proc Natl Acad Sci. 2006;103:3633–3638.
